# Metal-free synthesis of biarenes via photoextrusion in di(tri)aryl phosphates

**DOI:** 10.3762/bjoc.16.250

**Published:** 2020-12-08

**Authors:** Hisham Qrareya, Lorenzo Meazza, Stefano Protti, Maurizio Fagnoni

**Affiliations:** 1 PhotoGreen Lab, Department of Chemistry, viale Taramelli 12, 27100 Pavia, Italy; 2 Industrial Chemistry Department, Arab American University, 240 Jenin 13, Zababdeh, Palestine

**Keywords:** aryl phosphates, biarenes, metal-free synthesis, photochemistry, photoextrusion

## Abstract

A metal-free route for the synthesis of biarenes has been developed. The approach is based on the photoextrusion of a phosphate moiety occurring upon irradiation of biaryl- and triaryl phosphates. The reaction involves an exciplex as the intermediate and it is especially suitable for the preparation of electron-rich biarenes.

## Introduction

**Scheme 1:**
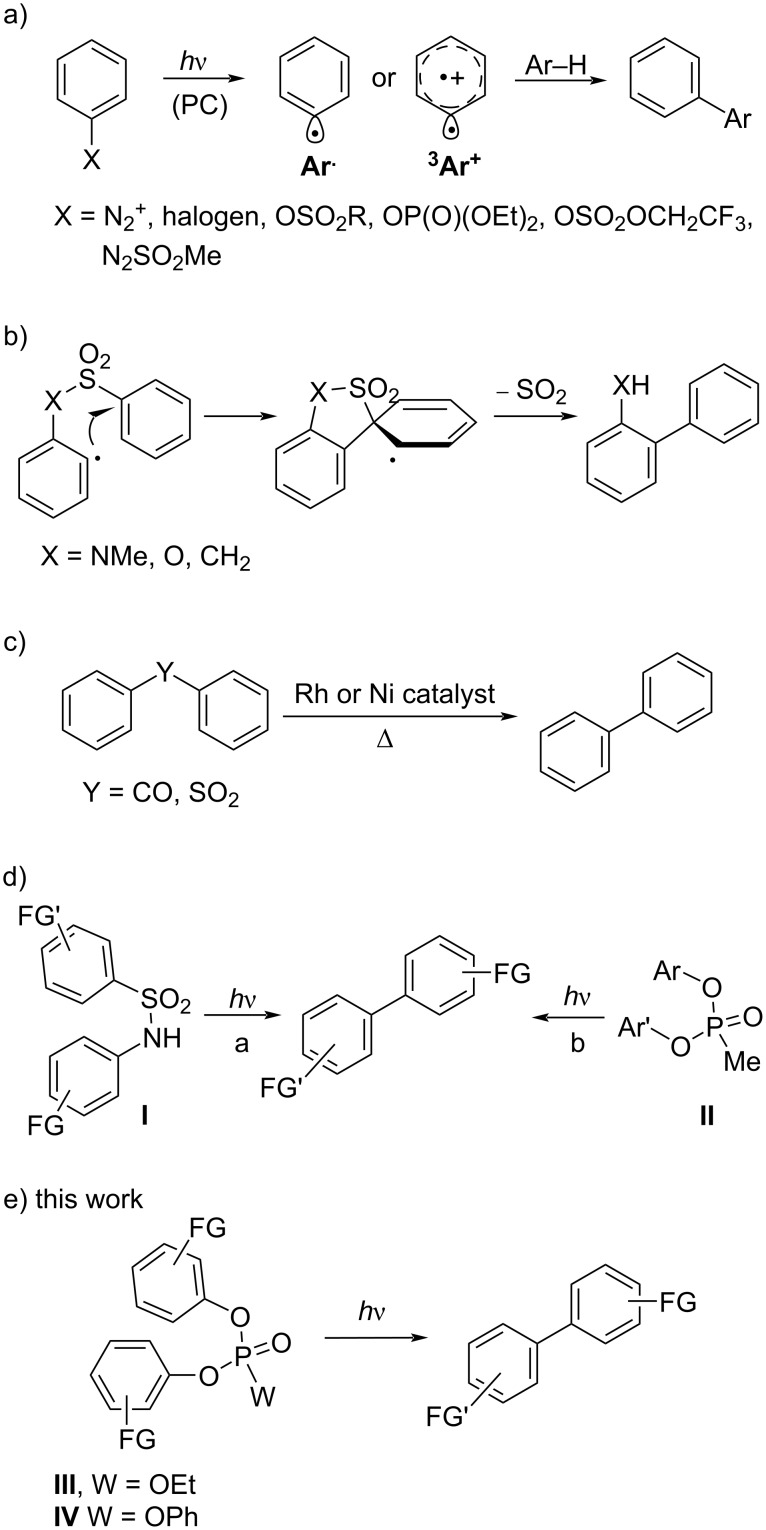
Synthesis of biarenes via a) photogenerated triplet aryl cations and aryl radicals (PC = photocatalyst), b) intramolecular free radical ipso substitution, c) thermally catalyzed extrusion of CO and SO_2_, d) photoinduced photoextrusion from benzene sulfonamides **I** and phosphonates **II**, and e) photoextrusion from diaryl- (**III**) and triaryl (**IV**) phosphates.

It is difficult to overestimate the importance of aromatics in drug development. Indeed, introducing an aromatic or a heteroaromatic ring, most often a (substituted) phenyl ring, into a biologically active compound is a common practice in medicinal chemistry [1–3]. In particular, the biaryl moiety is a privileged scaffold largely present in the skeleton of natural substances [4–7] and in useful chiral ligands [8–10]. The synthesis of biaryl derivatives remains, however, a considerable challenge [11–13]. Common methods, such as the Ullmann and Gomberg synthesis [14–16] have been nowadays supplanted by the much more versatile metal-catalyzed cross-coupling reactions [17–23]. This excellent approach still encounters some limitation in the scope and in the practical application, due to the use of labile and expensive reagents. Moreover, the elimination of metal trace residues and wastes is of some concern particularly for products destined to pharmaceutical applications as it is imperative operating under ‘green’ conditions. As for the last issue, there is nowadays a growing interest in the forging of Ar–Ar bonds under transition-metal-free conditions [24–25]. Apart the most common pathways, e.g., the Friedel–Crafts functionalization [26] or nucleophilic aromatic substitution [27], alternative approaches have emerged that make use of photogenerated intermediates (triplet aryl cations [28–29] or aryl radicals [30–31]). As for the former case, the intermolecular formation of a biaryl arose from the photoheterolysis of an Ar–N bond (in arene diazonium salts or their derivatives [32–33]), of an Ar–Cl bond [34–35], of an Ar–O bond (in aryl phosphates [36], aryl sulfonates [36], and in aryl trifluoroethyl sulfate [37], Scheme 1a) followed by the reaction of the thus formed aryl cation with an aromatic substrate. In an alternative approach, aryl radicals may be generated under photoredox catalysis conditions (mostly from arene diazonium salts or aryl iodides) [30–31] or by the direct photolysis of arylazo sulfones [38–40] and employed for the desired arylations. These reactions have the advantage of being applied to non-functionalized arenes but have the drawback to require a large excess of the nucleophilic reagent (the arene Ar–H) in up to 10–20-fold amount. Furthermore, the aryl radical/cation addition onto the aromatic reactant may lead to a mixture of regioisomers when using non-symmetrical Ar–H. A possible solution is having recourse to an intramolecular free radical ipso substitution reaction where an XSO_2_ tether is placed between two aromatic rings to direct the selective Ar–Ar bond formation (Scheme 1b) [41–43]). In this case, *N*-methyl sulfonamides were the elective substrates albeit a part of the tether is maintained in the final structure. This is common even for other related metal-free biaryl syntheses exploiting the Truce–Smiles rearrangement in aryl sulfonamides and aryl phenylsulfonates [44–46] or the [3,3]-sigmatropic rearrangement of sulfonium salts arising from the reaction of aryl sulfoxides and phenols [47]. To overcome this problem, the use of a metal catalyst (mainly Ni) was mandatory as reported for the real extrusion of CO in diaryl ketones [48–49] or of SO_2_ in diaryl sulfones (Scheme 1c) [50]. Nevertheless, a recent publication demonstrated that a metal-free photoextrusion was feasible when starting from benzene sulfonamides **I** (Scheme 1d, path a) [51]. Following the same approach, sparse reports described that in some cases biaryls may be obtained in variable yields starting from biaryl phosphates [52], biaryl phosphonates **II** [53–56], and triaryl phosphates [57–61] (Scheme 1d, path b). In search for alternative ways for the preparation of biaryls under photoinduced metal-free eco-sustainable conditions we reinvestigated the photochemistry of di- and triaryl phosphates **III** and **IV** (Scheme 1e), compounds that can be easily achieved from the corresponding phenols [62–63].

## Results and Discussion

At the onset of our investigation, we tested a triaryl phosphate such as 4-chlorophenyl diphenyl phosphate (**1a**), as the model compound in different solvents by irradiation in a multilamp reactor (wavelength centered at 310 nm, see Supporting Information File 1 for details). The obtained results are depicted in Table 1. Compound **1a** (0.02 M) was quite photostable in dichloromethane, acetonitrile, and acetone (Table 1, entries 1–3), whereas 4-chlorobiphenyl (**2a**) was observed in traces as the only product in neat methanol (30% of **1a** consumption, Table 1, entry 4). Interestingly, the addition of water (a methanol/water 2:1 mixture) increased the overall yield of the product **2a** (up to 16%) along with negligible amounts of biphenyl (**2b**). Decreasing the concentration of **1a** (to 10^−2^ M) in the examined conditions was found noxious for the reaction course (Table 1, entry 6), but shifting the wavelength to 254 nm led to significant amounts of the desired biaryl (Table 1, entry 7). The yields started to be satisfactory, however, when performing the reaction at 310 nm using 2,2,2-trifluoroethanol (TFE) as the solvent (45% yield, entry 8 in Table 1). We thus decided to replace part of the rather expensive and toxic solvent TFE with acetone (Table 1, entries 9–12) and the best results were obtained when using a TFE/acetone 4:1 mixture (Table 1, entry 10) with an isolated yield of **2a** of 67% along with **2b** (4% yield) as the byproduct. A further increase in the concentration of the substrate (Table 1, entries 13 and 14) resulted in a lowering of the selectivity (the undesired product **2b** was detected in up to 12% yield). Finally, no reaction took place when the solution was covered by an aluminum foil and stored in the photochemical apparatus for 24 h (Table 1, entry 15).

**Table 1:** Optimization of the reaction conditions.

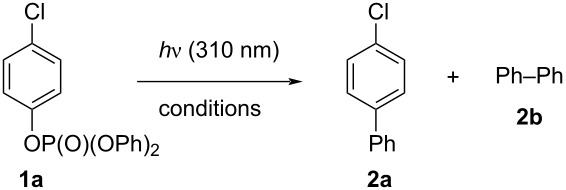			

entry	reaction conditions	λ_irr_ (nm)	products, yield (%)

1	**1a** (0.02 M), CH_2_Cl_2_	310	–^a^
2	**1a** (0.02 M), CH_3_CN	310	–^a^
3	**1a** (0.02 M), acetone	310	–^a^
4	**1a** (0.02 M), CH_3_OH	310	**2a**, 3^b^
5	**1a** (0.02 M), CH_3_OH/H_2_O 2:1	310	**2a**, 16; **2b**, 1
6	**1a** (0.01 M), CH_3_OH/H_2_O 2:1	310	**2a**, 6
7	**1a** (0.01 M), CH_3_OH/H_2_O 2:1	254	**2a**, 44
8	**1a** (0.02 M), CF_3_CH_2_OH	310	**2a**, 45; **2b**, 2
9	**1a** (0.02 M), CF_3_CH_2_OH/acetone 9:1	310	**2a**, 38; **2b**, 2
10	**1a (0.02 M), CF_3_CH_2_OH/acetone 4:1**	**310**	**2a, 67; 2b, 4**
11	**1a** (0.02 M), CF_3_CH_2_OH/acetone 7:3	310	**2a**, 48; **2b**, 3
12	**1a** (0.02 M), CF_3_CH_2_OH/acetone 1:1	310	**2a**, 14; **2b**, 2
13	**1a** (0.04 M), CF_3_CH_2_OH/acetone 4:1	310	**2a**, 57; **2b**, 2
14	**1a** (0.06 M), CF_3_CH_2_OH/acetone 4:1	310	**2a**, 67; **2b**, 12
15	**1a** (0.02 M), CF_3_CH_2_OH/acetone 4:1^c^		–^a^

^a^No consumption of **1a** observed; ^b^30% consumption of **1a** measured; ^c^the reaction mixture was stored in the dark for 24 h.

**Scheme 2:**
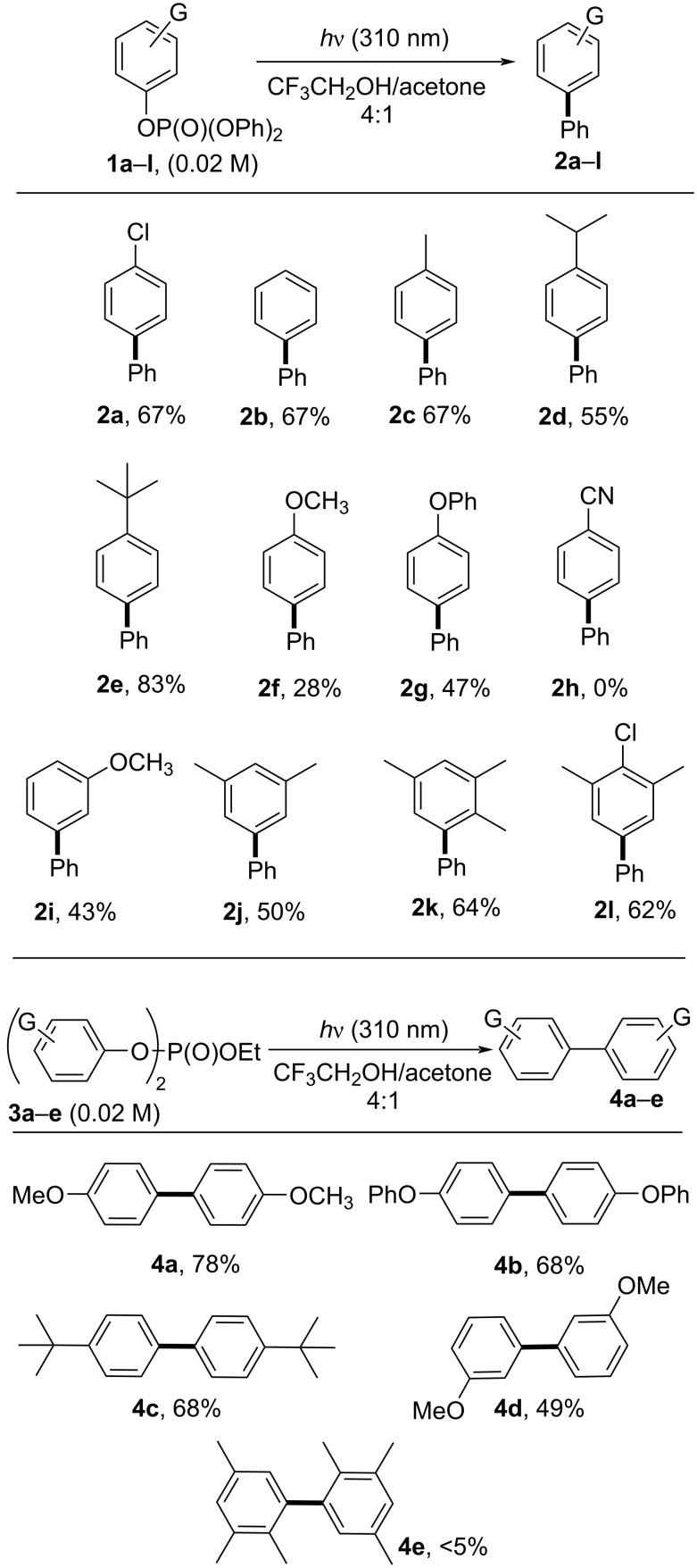
Metal-free photochemical synthesis of biaryls **2** and **4**.

Encouraged by the results collected in Table 1, in particular with the fact that the byproduct **2b** was formed in such small amounts, we used the conditions described in entry 10 (Table 1) to explore the scope of the process by investigating other *n*-substituted phenyl diphenyl phosphates (**1a–l**, see Scheme 2). Thus, the irradiation of triphenyl phosphate (**1b**) gave the corresponding biphenyl (**2b**) in 67% yield. Similar results were obtained with 4-alkylphenyl diphenyl phosphates, that afforded the 4-substituted biaryls **2c–e** in up to 83% yield. However, when examining substrates bearing a strong electron-donating substituent (G = 4-OMe, 4-OPh, 3-OMe), the efficiency of the process decreased (see the yields of **2f, g**, and **2i** in Scheme 2). On the other hand, the presence of an electron-withdrawing group (e.g., 4-CN, compound **1h**) completely inhibited the reaction and **1h** was recovered unaltered after the irradiation. Better results have been, however, obtained with polysubstituted derivatives **1j–l**. In these cases, the expected phenylated arenes **2j–l** were isolated in the 50–64% range.

We were then intrigued to extend the scope of the reaction by focusing on a few ethyl diaryl phosphates **3a–e**. Gratifyingly, the formation of the symmetric biaryls **4** took place efficiently with the substrates bearing strong electron-donating substituents, especially when present in the *para*-position (see the case of **4a–c**). Unfortunately, the unsymmetric biaryl **4e** was detected in a very poor amount.

To investigate the reaction mechanism, some photophysical parameters of compounds **1** and **3** were determined. All the phosphates examined were barely fluorescent in methanol, with an emission quantum yield (Φ_em_) in the 0.005–0.06 range (see Table 2 and Supporting Information File 1 for further details).

**Table 2:** Emission data of selected diaryl- and triaryl phosphates **1** and **3**.

compound	λ_em_ (nm)	Φ_em_^a^

**1a**	300	0.005
**1b**	315	0.017
**1e**	319	0.025
**1f**	312	0.059
**1h**	335	0.023
**3a**	307, 360	0.030
**3c**	294	0.062

^a^Measured by comparison with 4-chloroanisole (Φ_F_ = 0.019 in MeOH) [64].

**Figure 1:**
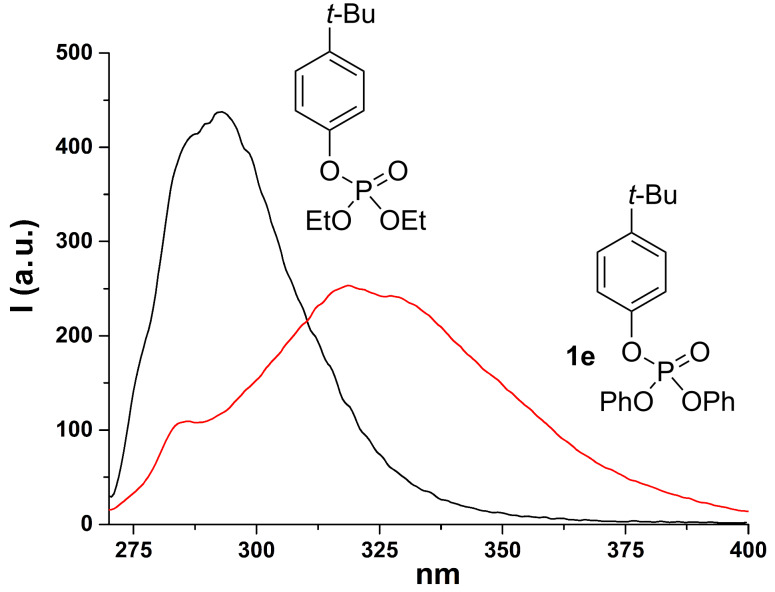
Emission spectrum of compound **1e** (red) and of diethyl *p*-*tert*-butylphenyl phosphate (black) in methanol.

**Figure 2:**
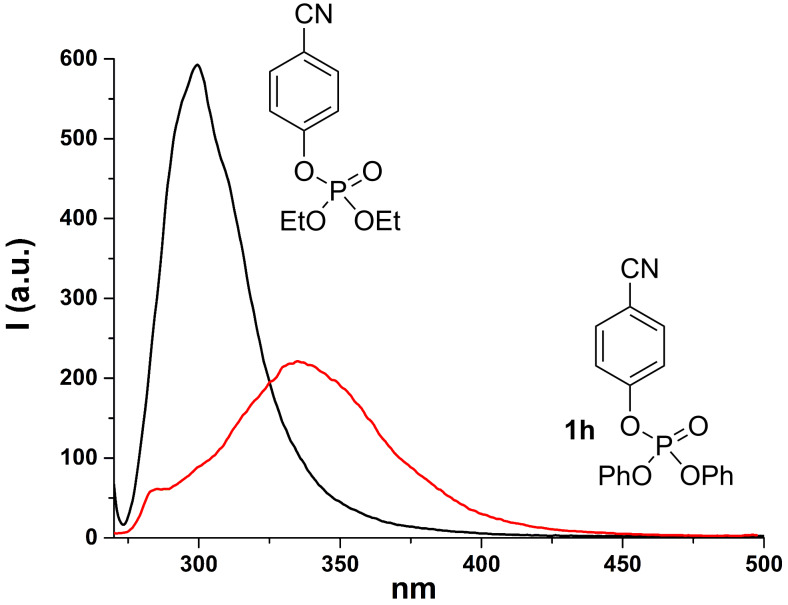
Emission spectrum of compound **1h** (red) and of diethyl *p*-cyanophenyl phosphate (black) in methanol.

**Figure 3:**
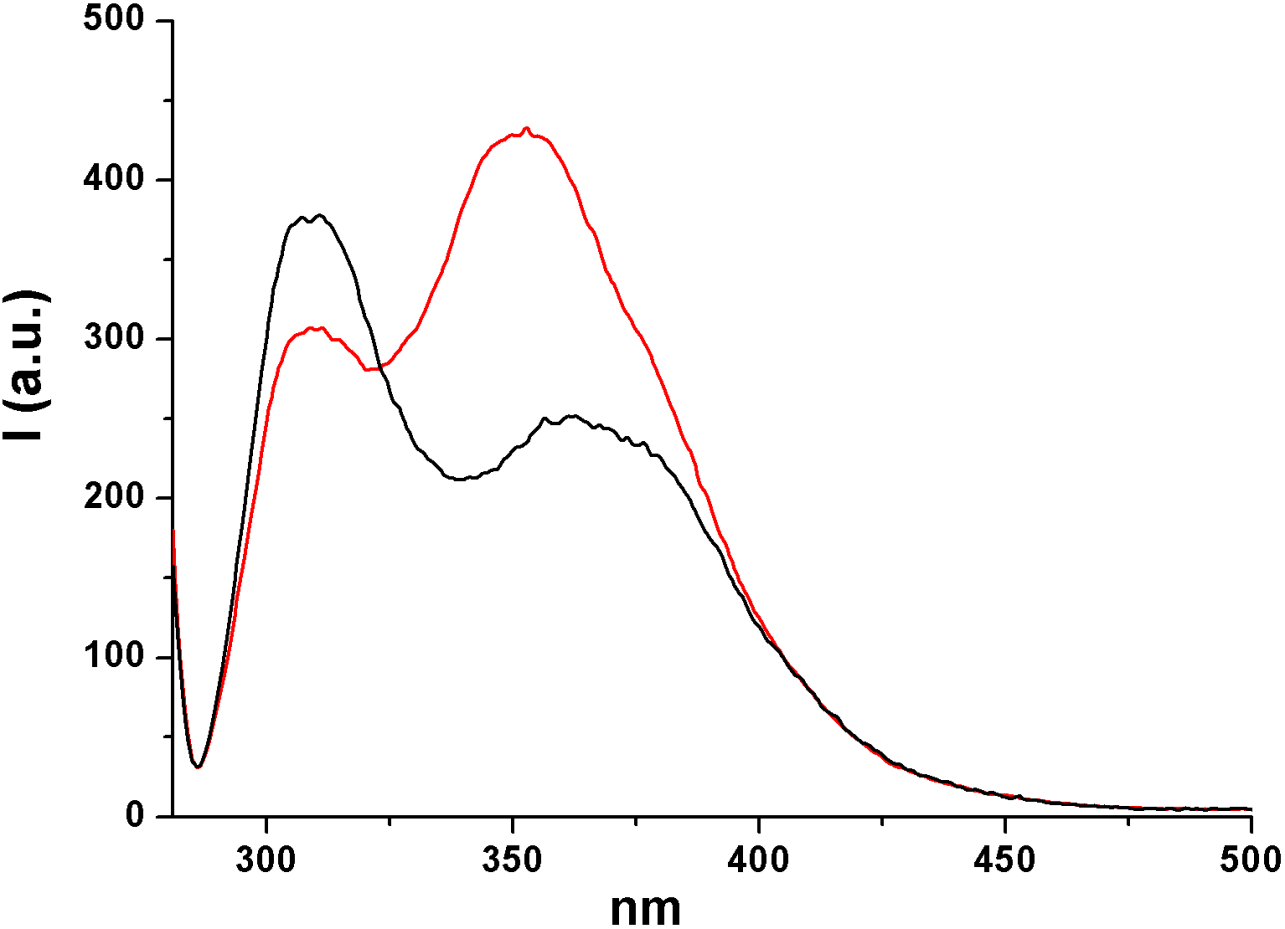
Emission spectrum of compound **3a** in methanol (black) and in a methanol/TFE 4:1 mixture (red).

**Figure 4:**
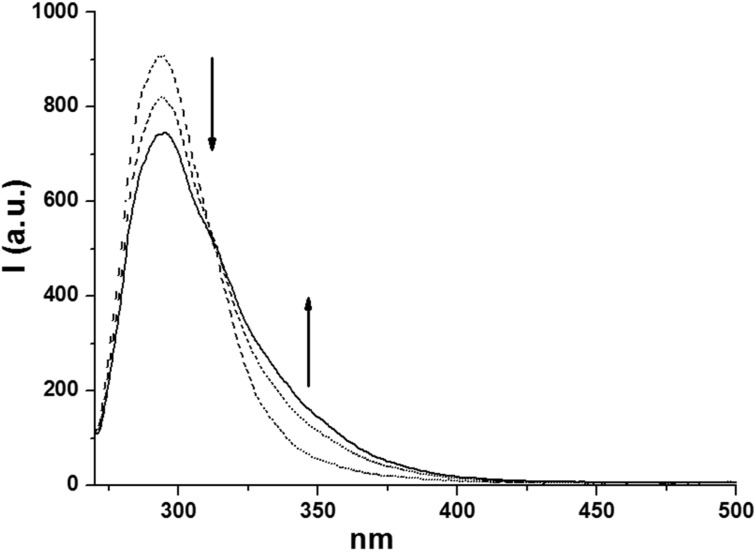
Emission spectrum of **3c** in MeOH (dotted line) and in the presence of increasing amounts of TFE (up to 20% v/v, continuous line).

We thus focused on compounds **1e**, **1h**, **3a** and **3c** as the model substrates. In the case of compounds **1e** and **1h**, we observed that the fluorescence is significantly red shifted (about 30 nm) with respect to that of the corresponding diethyl aryl phosphates (see Figure 1 and Figure 2). On the other hand, when focusing on compound **3a**, we noticed the presence of two emission bands located at 307 and 360 nm, respectively (see Figure 3) and their relative intensity was solvent dependent. Indeed, the band at 360 nm is favored and slightly blue shifted when increasing the proticity of the medium (see the comparison of the fluorescence spectra obtained in methanol and in a methanol/TFE 4:1 mixture, Figure 3). A similar behavior was observed with compound **3c**, where a single emission band located at ca. 290 nm is observable in neat methanol, whereas the presence of TFE causes a lowering of that emission, in favor of a second band in the 330–350 nm region (Figure 4). These two emissions have been assigned, on the basis of our results and of the reported literature [58,61], to the singlet monomeric excited state and to the exciplex, respectively.

While aryl phosphates have been only sparsely used as substrates in thermal cross-coupling reactions [65–67], their photochemical behavior has been the subject of various investigations in the last decades [28–29,64,68]. Simple (electron-rich) monoaryl phosphates are known to undergo the photoheterolysis of the Ar–O bond to form aryl cations [28,36]. The presence of an electron-withdrawing group (e.g., NO_2_) may, however, divert the reactivity since a photoinduced solvolysis occurred as demonstrated by Havinga more than 70 years ago [69]. In alternative, the irradiation of monoaryl phosphates in the presence of a strong nucleophile (e.g., a tin anion) led to an ipso substitution reaction via an ArS_RN_1 process [70].

**Scheme 3:**
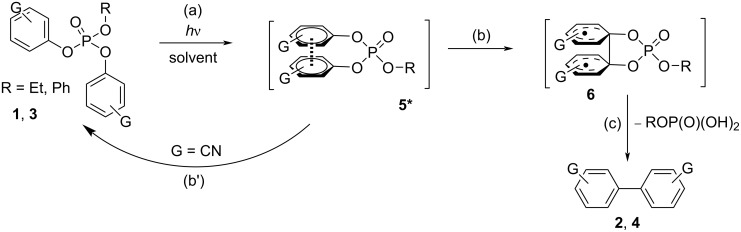
Photoreactivity of aryl phosphates **1** and **3** in protic media.

The situation dramatically changes when a further aryl group is present in the phosphates since none of the above-mentioned processes took place. In fact, our investigations, in according with early works [52,71], suggested that both diaryl and triaryl phosphates are prone to generate an intramolecular exciplex 5* under irradiation (Scheme 3, path (a)), on the route to the extrusion of the phosphate moiety. This is demonstrated by the formation of a new emission band when more than one aryl group is present in the aryl phosphate (see Figure 1). In our investigation, we likewise stated that the formation of 5* (from **1** and **3**) is highly favored in highly protic solvents such as TFE, as well evidenced, among the others, in the cases of **1e** and **4c**, and as already reported in the formation of other intramolecular aromatic exciplexes [72].

The so generated exciplex apparently plays a key role in the formation of the biaryls **2** and **4** probably via the formation of the biradical intermediate **6** [55] preceding the loss of ROP(O)(OH)_2_ (paths (b), (c), Scheme 3). The long irradiation time required to achieve a complete consumption of the substrates **1** and **3** is in accordance with the low quantum yield values reported for this process [58,61]. Furthermore, a dependence on the nature of the aromatic substituents G was apparent, since arylation took place (in variable yields) with electron-rich aromatic substituents, while it was completely inhibited in the presence of electron-withdrawing groups (path (b’), in Scheme 3). In the case of the triaryl phosphates, biphenyl (**1b**) is formed as the byproduct in <5% yield.

## Conclusion

We demonstrated that biaryls can be smoothly prepared via the photoextrusion of diaryl- and triaryl phosphates in protic media, with the concomitant release of a molecule of phosphoric acid monoester. The reaction takes place in moderate yields but under very mild conditions with no need of any (photo)catalyst or additive, despite the scope of the process is in part limited since the presence of at least one electron-withdrawing group on an aromatic ring completely suppressed the reaction.

## Supporting Information

File 1 Experimental section, fluorescence, and ^1^H and ^13^C NMR spectra.
