# Supplementary material for: Metal-free synthesis of biarenes via photoextrusion in di(tri)aryl phosphates
Source: Beilstein J Org Chem. 2020 Dec 8;16:3008–14. doi: 10.3762/bjoc.16.250 (PMC7736689; doi:10.3762/bjoc.16.250)

BEILSTEIN JOURNAL OF ORGANIC CHEMISTRY

Supporting Information

for

Metal-free synthesis of biarenes via photoextrusion in di(tri)aryl phosphates

Hisham Qrareya, Lorenzo Meazza, Stefano Protti and Maurizio Fagnoni

Beilstein J. Org. Chem. **2020,** *16,* 3008–3014. doi:10.3762/bjoc.16.250

Experimental section, fluorescence, and ^{1}H and ^{13}C NMR spectra

License and Terms: This is a supporting information file under the terms of the Creative Commons Attribution License (https://creativecommons.org/licenses/by/4.0). Please note that the reuse, redistribution and reproduction in particular requires that the author(s) and source are credited and that individual graphics may be subject to special legal provisions.
The license is subject to the *Beilstein Journal of Organic Chemistry* terms and conditions: (https://www.beilstein-journals.org/bjoc/terms)

Table of contents

1. Experimental details S1

2. Fluorescence spectra of triaryl- and ethyl diaryl phosphates examined in the present work S7

3. References S12

4. Copies ^{1}H and ^{13}C NMR of compounds **1a**–**l**, **2a**, **2c**–**l**, **3a**–**e**, **4b**–**d** S14

1. Experimental details

General

The starting materials, solvents (HPLC grade with the only exception of 2,2,2-trifluoroethanol (TFE), that was available in >99% purity), and other chemicals used in the experiments were commercially available and used without further purification. ^{1}H and ^{13}C NMR spectra were recorded on a 300 MHz spectrometer, chemical shifts were reported in ppm downfield from TMS, and the attributions were made on the basis of ^{1}H and ^{13}C signals, as well as DEPT-135 experiments; chemical shifts are reported in ppm downfield from TMS. The reaction course was followed by means of TLC and GC analyses.

Synthesis of triaryl phosphates 1a–l. Compounds **1a–l** have been prepared by following a known procedure.[1] A suspension of the chosen phenol (4.03 mol) in toluene (30 mL) was vigorously mixed with NaOH (20% aq, 1 mL) and benzyltriethylammonium chloride (0.13 mmol). The so obtained suspension was cooled to 0 °C and then diphenyl chlorophosphate (0.84 mL, 4.03 mmol, 1 equiv) was added dropwise. The reaction mixture was refluxed under stirring overnight and, after cooling to room temperature, washed with water (3 × 20 mL). The organic phase was dried over Na_2SO_4, evaporated in vacuo, and the crude residue purified by column chromatography (eluent: petroleum ether/ethyl acetate mixture).

4-Chlorophenyl diphenyl phosphate (1a). Deliquescent colorless solid, 87% yield. Spectroscopical data for compound **1a** were in accordance with the literature data.[2]

Triphenyl phosphate (1b). Colorless solid, mp = 43.4-45.5 °C, 88% yield. Spectroscopical data for compound **1b** were in accordance with the literature data.[2]

4-Methylphenyl diphenyl phosphate (1c). Oil, 84% yield. Spectroscopical data for compound **1c** were in accordance with the literature data.[2]

4-Isopropylphenyl diphenyl phosphate (1d). Oil, 87% yield. ^{1}H NMR (300 MHz, $CDCl_3$, δ): 1.23-1.26 (d, 6H, J = 7 Hz), 2.87-2.94 (qui, 1H, J = 7Hz), 7.18-7.29 (m, 11H), 7.35-7.40 (m, 3H). ^{13}C NMR (75 MHz, $CDCl_3$, δ): 23.9 (CH_3), 33.4 (CH), 119.7 (CH), 119.8 (CH), 125.2, 125.4 (CH), 127.6 (CH), 129.6, 129.7 (CH), 146.1, 150.3, 150.4. Anal. Calcd for $C_{21}H_{21}O_4P$: C, 68.47; H, 5.75. Found: C 68.6, H, 5.8.

4-*tert*-Butylphenyl diphenyl phosphate (1e). Oil, 85% yield ^{1}H NMR (300 MHz, $CDCl_3$, δ): 1.32 (s, 9H), 7.16-7.29 (m, 6H), 7.35-7.40 (m, 5H). ^{13}C NMR (75 MHz, $CDCl_3$, δ): 31.3 (CH_3), 34.3, 119.3 (CH), 120.1 (CH), 125.4 (CH), 126.6 (CH), 129.7 (CH), 148.0, 148.4, 150.3, 150.5. Anal. Calcd for $C_{22}H_{23}O_4P$: C, 69.10; H, 6.06. Found: C 69.2, H, 5.9.

4-Methoxyphenyl diphenyl phosphate (1f). Oil, 86% yield. Spectroscopical data for compound **1c** were in accordance with the literature data.[2]

4-Phenoxyphenyl diphenyl phosphate (1g). Oil, 45% yield. ^{1}H NMR (300 MHz, $CDCl_3$, δ): 6.99-7.02 (m, 4H), 7.13-7.15 (m, 1H), 7.21-7.29 (m, 8H), 7.34-7.41 (m, 6H). ^{13}C NMR (75 MHz, $CDCl_3$, δ): 118.6 (CH), 119.9 (CH), 120.4 (CH), 121.2 (CH), 121.3 (CH), 123.4 (CH), 125.5 (CH), 129.8 (CH), 145.7, 150.3, 150.4, 154.5, 157.0. Anal. Calcd for $C_{24}H_{19}O_5P$: C, 68.90; H, 4.58. Found: C 69.0, H, 4.6.

4-Cyanophenyl diphenyl phosphate (**1h**). Colorless solid, 45% yield. mp = 59.3-60.5 °C ^{1}H NMR ($CDCl_3$, δ): 7.24-7.28 (m, 6H), 7.40-7.42 (m, 6H), 7.67-7.70 (d, 2H, J = 8.5 Hz). ^{13}C NMR ($CDCl_3$, δ): 109.5, 117.9, 119.8 (CH), 119.9 (CH), 121.0 (CH), 121.1 (CH), 125.9 (CH), 129.9 (CH). 134.1 (CH), 150.0, 150.1, 153.4. Anal. Calcd for $C_{19}H_{14}NO_4P$: C, 64.96; H, 4.02; N, 3.99. Found: C 65.0, H, 4.2; N, 3.8,

3-Methoxyphenyl diphenyl phosphate (**1i**). Oil, 64% yield. Spectroscopical data for compound **1i** were in accordance with the literature data.[3]

3,5-Dimethylphenyl diphenyl phosphate (1j). Oil, 61% yield. ^{1}H NMR (300 MHz, $CDCl_3$, δ): 2.31 (s, 6H), 6.87 (s, 2H), 7.17-7.33 (m, 6H), 7.35-7.40 (m, 5H). ^{13}C NMR ($CDCl_3$, δ): 21.1 (CH_3), 117.5 (CH), 120.0 (CH), 120.1 (CH), 125.4 (CH), 127.1, 129.7 (CH), 139.6, 150.1, 150.4. Anal. Calcd for $C_{20}H_{19}O_4P$: C, 67.79; H, 5.40. Found: C 67.6, H, 5.2.

2,3,5-Trimethylphenyl diphenyl phosphate (1k). Oil, 57% yield. ^{1}H NMR (300 MHz, $CDCl_3$, δ): 2.08 (s, 3H), 2.25 (s, 3H), 2.28 (s, 3H), 6.86 (s, 1H), 7.04 (s, 1H), 7.18-7.23 (m, 6H), 7.25-7.40 (m, 4H). ^{13}C NMR ($CDCl_3$, δ): 11.8 (CH_3), 19.9 (CH_3), 20.8 (CH_3), 117.8 (CH), 120.1 (CH), 124.5, 125.4 (CH), 127.7 (CH), 129.7 (CH), 136.0, 138.4, 148.6, 150.6. Anal. Calcd for $C_{21}H_{21}O_4P$: C, 68.47; H, 5.75. Found: C 68.6, H, 5.7.

4-Chloro-3,5-dimethylphenyl diphenyl phosphate (1l). Oil, 78% yield. 2.37 (s, 6H), 6.99 (s, 2H), 7.25-7.28 (m, 6H), 7.36-7.41 (m, 4H). ^{13}C NMR ($CDCl_3$, δ): 20.7 (CH_3), 119.7 (CH), 120.0 (CH), 125.6 (CH), 129.8 (CH), 137.3, 137.8, 147.8, 150.3. Anal. Calcd for $C_{20}H_{18}ClO_4P$: C, 61.79; H, 4.67. Found: C 61.6, H, 4.5.

Synthesis of diaryl ethyl phosphates 3a–e. The synthesis of compounds **3a–e** has been carried out by following a reported procedure.[4] The chosen phenol (8 mmol) was added in small portions to a stirred suspension of NaH (60%, 8 mmol) in dry THF. The resulting mixture was stirred, then ethyl dichlorophosphate (4 mmol) was added dropwise. The resulting mixture was stirred until the complete consumption of the starting phenol. Then, the solvent was evaporated in vacuo, the

residue was dissolved in ether (20 mL), and washed with NaOH (5% aq, 20 mL). The organic phase was washed with brine (2 × 20 mL), water (2 × 20 mL), dried over Na_2SO_4, and evaporated. The crude product was finally purified by column chromatography (eluent: petroleum ether/ethyl acetate mixture).

Ethyl bis(4-methoxyphenyl) phosphate (3a). Oil, 77% yield. Spectroscopical data for compound **3a** were in accordance with the literature data.[5]

Ethyl bis(4-phenoxyphenyl) phosphate (3b). Oil, 51% yield. ^{1}H NMR (300 MHz, $CDCl_3$, δ): 1.39-1.44 (t, 3H, J = 7 Hz), 4.32-4.37 (qui, 2H, J = 7 Hz), 6.99-7.03 (m, 8H), 7.20-7.25 (m, 6H), 7.33-7.38 (m, 4H). ^{13}C NMR ($CDCl_3$, δ): 16.0 (CH_3), 65.5 (CH_2), 118.6 (CH), 119.9 (CH), 121.1 (CH), 123.3 (CH), 129.7 (CH), 145.9, 154.3, 157.1. Anal. Calcd for $C_{26}H_{23}O_6P$: C, 67.53; H, 5.01. Found: C 67.6, H, 4.8.

Ethyl bis(4-*tert*-butylphenyl) phosphate (3c). Deliquescent solid, 84% yield. Spectroscopical data for compound **3b** were in accordance with the literature data.[6]

Ethyl bis(3-methoxyphenyl) phosphate (3d). Oil, 75% yield. ^{1}H NMR (300 MHz, $CDCl_3$, δ): 1.37-1.45 (t, 3H, J = 7Hz), 3.81 (s, 6H), 4.30-4.35 (qui, 2H, J = 7 Hz), 6.74-6.80 (m, 3H), 6.90-6.95 (m, 2H), 7.22-7.28 (t, 3H, J = 8 Hz). ^{13}C NMR ($CDCl_3$, δ): 16.0 (CH_3), 55.3 (CH_3), 65.4 (CH2), 106.0 (CH), 111.0 (CH), 112.1 (CH), 130.0 (CH), 151.3, 156.6. Anal. Calcd for $C_{16}H_{19}O_6P$: C, 56.81; H, 5.66. Found: C 57.0, H, 5.5.

Ethyl bis(2,3,5-trimethylphenyl) phosphate (3e). Oil, 48% yield. ^{1}H NMR (300 MHz, $CDCl_3$, δ): 1.34-1.37 (t, 3H, J = 7 Hz), 2.12 (s, 3H), 2.25 (s, 3H), 2.27 (s, 3H), 4.31-4.35 (qui, 2H, J = 7 Hz), 6.83 (s, 1H), 7.01 (s, 1H). ^{13}C NMR ($CDCl_3$, δ): 11.8 (CH_3), 16.0 (CH_3), 19.9 (CH_3), 20.7 (CH_3), 65.1 (CH_2), 117.9 (CH), 124.5, 127.3 (CH), 135.8, 138.2, 148.9. Anal. Calcd for $C_{20}H_{27}O_4P$: C, 66.28; H, 7.51. Found: C 66.2, H, 7.4.

General procedure for the preparation of biaryls 2 and 4. A solution of the chosen triaryl phosphate **1** or of the ethyl (diaryl) phosphate **3** (0.02 M) in a CF_3CH_2OH/acetone 4:1 mixture (10 mL) was argon saturated, then irradiated by means of a multilamp reactor (a Rayonet-like reactor purchased by Helios Italquartz, Italy) equipped with 10 phosphor-coated Hg lamps (15 W, λ_{em} = 310 nm)[7] for 24 hours. The photolyzed solution was then evaporated and the residue purified via column chromatography (eluent: petroleum ether/ethyl acetate mixture).

4-Chlorobiphenyl (2a). From 72 mg (0.2 mmol) of **1a** in 10 mL of CF_3CH_2OH/Acetone 4:1. Purification by column chromatography (eluant: petroleum ether-ethyl acetate 95:5) afforded **2a**

(29 mg, colorless solid, 67% yield). Spectroscopical data of **2a** were in accordance with the literature.[8]

Biphenyl (2b). From 65 mg (0.2 mmol) of **1b** in 10 mL of CF_3CH_2OH-Acetone 4-1 mixture. Purification by column chromatography (eluant: petroleum ether) gave **2a** (21 mg, colorless solid, 67% yield). Spectroscopical data of **2b** were in accordance with the literature data[8] and with the NMR spectra of a commercially available sample.

4-Methylbiphenyl (2c). From 68 mg (0.2 mmol) of **1c** in 10 mL of CF_3CH_2OH/Acetone 4:1 mixture. Purification by column chromatography (eluant: petroleum ether-ethyl acetate 95:5) afforded **2c** (23 mg, colorless solid, 67% yield). Spectroscopical data of **2c** were in accordance with the literature.[9]

4-Isopropylbiphenyl (2d). From 73.6 mg (0.2 mmol) of **1d** in 10 mL of CF_3CH_2OH/Acetone 4:1 mixture. Purification by column chromatography (eluant: petroleum ether) afforded **2d** (21.5 mg, oil, 55% yield). Spectroscopical data of **2c** were in accordance with the literature data.[10]

4-*tert*-Butylbiphenyl (2e). From 76.5 mg (0.2 mmol) of **1e** in 10 mL of CF_3CH_2OH/Acetone 4:1 mixture. Purification by column chromatography (eluant: petroleum ether) gave **2e** (35 mg, colorless solid, 83% yield). Spectroscopical data of **2e** were in accordance with the literature.[11]

4-Methoxybiphenyl (2f). From 71 mg (0.2 mmol) of **1f** in 10 mL of CF_3CH_2OH/Acetone 4:1 mixture. Purification by column chromatography (eluant: petroleum ether:ethyl acetate 9:1) yielded **2f** (10.5 mg, colorless solid, 28% yield). Spectroscopical data of **2f** were in accordance with the literature.[11]

4-Phenoxybiphenyl (2g). From 83.7 mg (0.2 mmol) of **1g** in 10 mL of CF_3CH_2OH/Acetone 4:1 mixture. Purification by column chromatography (eluant: petroleum ether: ethyl acetate 9:1) afforded **2g** (23 mg, colorless solid, 47 % yield). Spectroscopical data of **2g** were in accordance with the literature.[12]

3-Methoxybiphenyl (2i). From 71 mg (0.2 mmol) of **1i** in 10 mL of CF_3CH_2OH/Acetone 4:1 mixture. Purification by column chromatography (eluant: petroleum ether:ethyl acetate 9:1) gave **2f** (19 mg, colorless solid, 43% yield). Spectroscopical data of **2i** were in accordance with the literature.[13]

3,5-Dimethylbiphenyl (2j). From 71 mg (0.2 mmol) of **1j** in 10 mL of CF_3CH_2OH/Acetone 4:1 mixture. Purification by column chromatography (eluant: petroleum ether:ethyl acetate 9:1) afforded **2j** (10 mg, colorless solid, 50% yield). Spectroscopical data of **2j** were in accordance with the literature.[13]

2,3,5-Trimethylbiphenyl (2k). From 73.7 mg (0.2 mmol) of **1k** in 10 mL of CF_3CH_2OH/Acetone 4:1 mixture. Purification by column chromatography (eluant: petroleum ether:ethyl acetate 9:1)

gave **2k** (25 mg, colorless solid, 64% yield). Spectroscopical data of **2k** were in accordance with the literature. Spectroscopical data for compound **2k** were in accordance with the literature data.[14]

3,5-Dimethyl-4-chlorobiphenyl (2l). From 78 mg (0.2 mmol) of **1l** in 10 mL of CF_3CH_2OH/Acetone 4:1 mixture. Purification by column chromatography (eluant: petroleum ether:ethyl acetate 9:1) afforded **2l** (27 mg, colorless oil, 62% yield). Spectroscopical data of **2l** were in accordance with the literature.[15]

4,4'-dimethoxybiphenyl (4a). From 67.7 mg (0.2 mmol) of **3a** in 10 mL of CF_3CH_2OH/Acetone 4:1 mixture. Purification by column chromatography (eluant: petroleum ether:ethyl acetate 9:1) gave **4a** (33.4 mg, colorless solid, 78% yield). Spectroscopical data of **4a** were in accordance with the literature[16] and with the NMR spectra of a commercially available sample.

4,4'-Phenoxybiphenyl (4b). From 92.5 mg (0.2 mmol) of **3b** in 10 mL of CF_3CH_2OH/Acetone 4:1 mixture. Purification by column chromatography (eluant: petroleum ether:ethyl acetate 9:1) afforded **4b** (46 mg, colorless solid, 68% yield). Spectroscopical data of **4b** were in accordance with the literature.[17]

4,4'-Di-*tert*-butylbiphenyl (4c). From 78.1 mg (0.2 mmol) of **3c** in 10 mL of CF_3CH_2OH/Acetone 4:1 mixture. Purification by column chromatography (eluant: petroleum ether:ethyl acetate 9:1) yielded **4c** (36.2 mg, colorless solid, 68% yield). Spectroscopical data of **4c** were in accordance with the literature.[18]

3,3'-Phenoxybiphenyl (4d). From 67.7 mg (0.2 mmol) of **3d** in 10 mL of CF_3CH_2OH/Acetone 4:1 mixture. Purification by column chromatography (eluant: petroleum ether:ethyl acetate 9:1) afforded **4d** (21 mg, colorless solid, 49% yield). Spectroscopical data of **4d** were in accordance with the literature.[19]

2. Fluorescence spectra of selected triaryl- and ethyl diaryl phosphates examined in the present work.

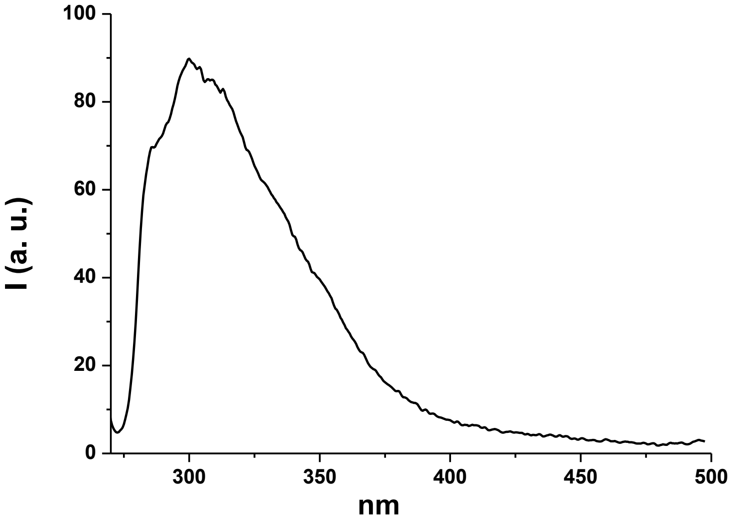

Figure S1. Emission spectrum of **1a** in MeOH.

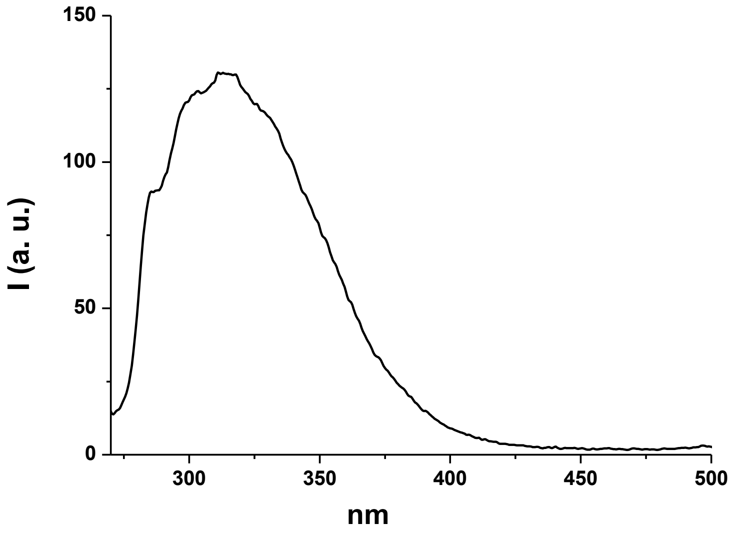

Figure S2. Emission spectrum of **1b** in MeOH.

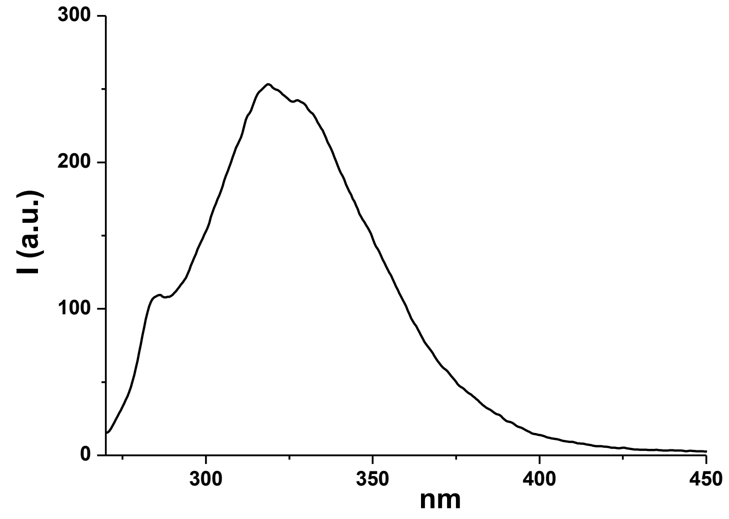

Figure S3. Emission spectrum of **1e** in MeOH.

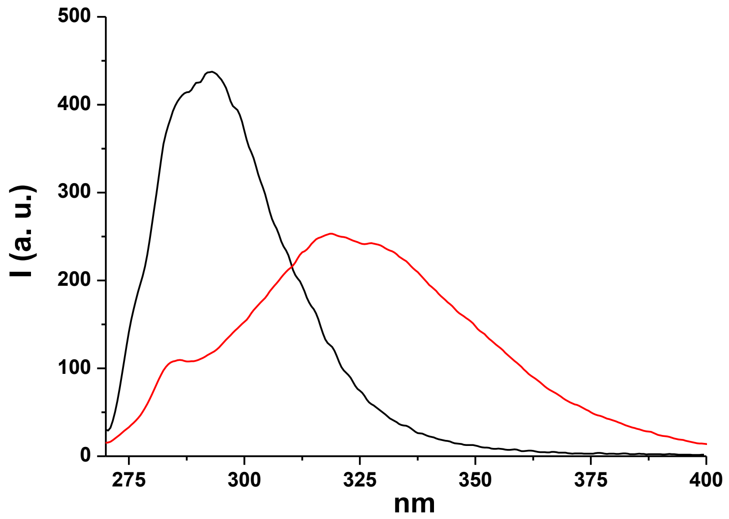

Figure S4. Emission spectrum of **1e** (black) and diethyl 4-*tert*-butylphenyl phosphate (red) in MeOH.

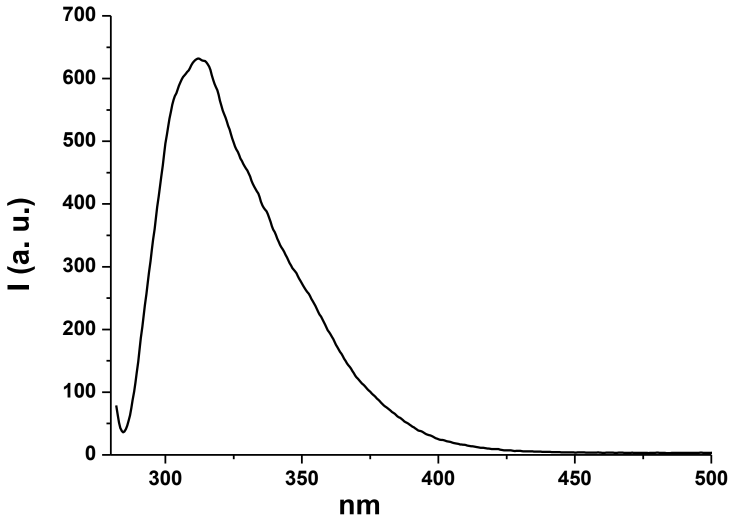

Figure S5. Emission spectrum of **1f** in MeOH.

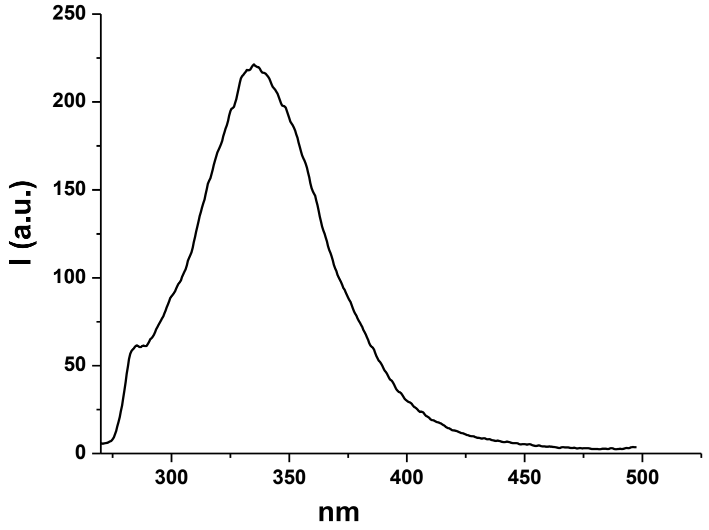

Figure S6. Emission spectrum of **1h** in MeOH.

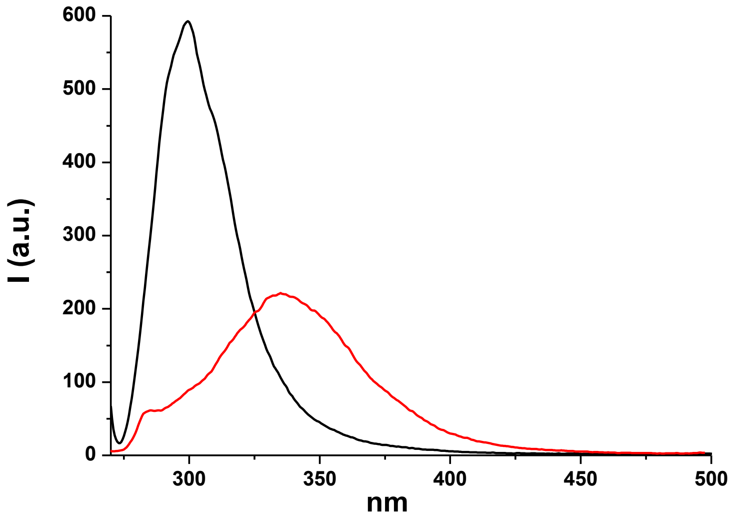

Figure S7. Emission spectrum of **1h** (red) and of diethyl *p*-cyanophenyl phosphate (black) in MeOH.

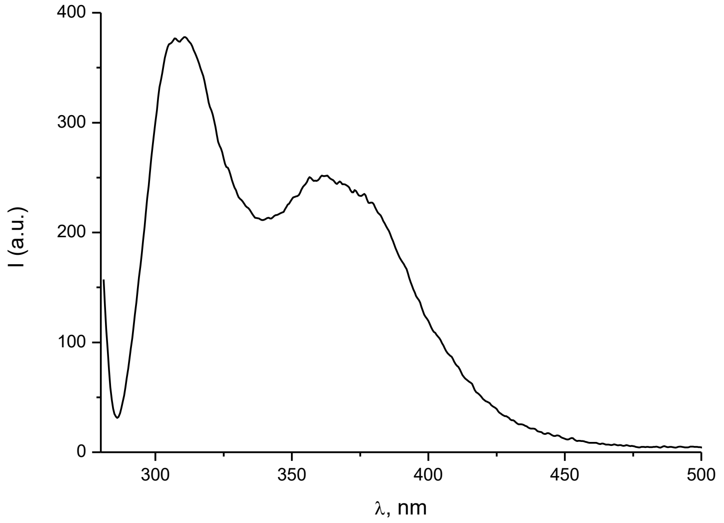

Figure S8. Emission spectrum of **4a** in MeOH

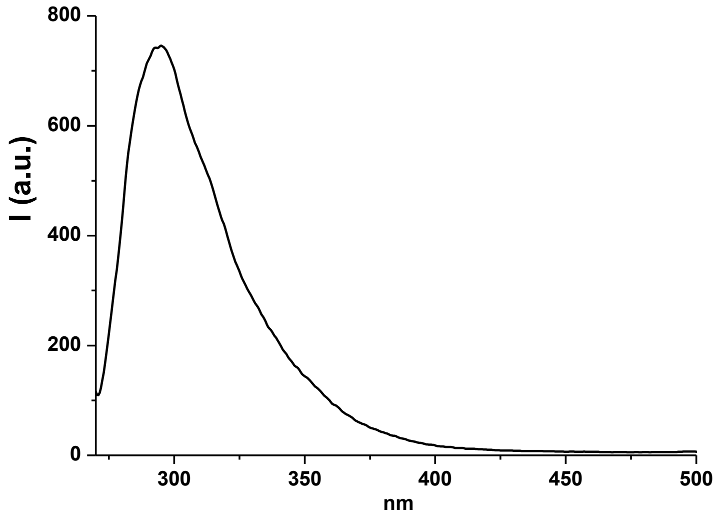

Figure S9. Emission spectrum of **4c** in MeOH

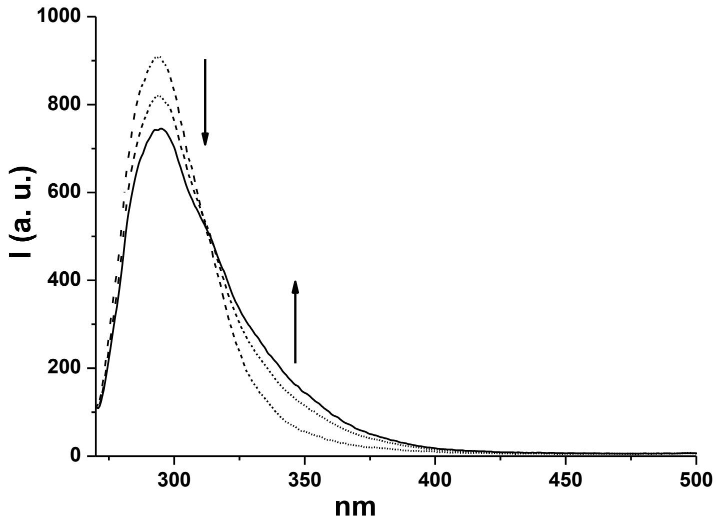

Figure S10. Emission spectrum of **4c** in MeOH (dotted line) and in the presence of increasing amounts of 2,2,2-trifluoroethanol (up to 20% v/v, continuous line)

3. References.

1. Genkina, G. K.; Shipov, A. E.; Mastryukova, T. A.; Kabachnik, M. I. *Russian J. Gen. Chem.*, **1996**, *66*, 1742–1744.
2. Ying, J.; Gao, Q.; Wu, X.-F. *Chem Asian J.* **2020**, *15*, 1540–1543. DOI: 10.1002/asia.202000154
3. Jones, S.; Selitsianos, D.; Thompson, K. J.; Toms, S. M. *J. Org. Chem.* **2003**, *68*, 5211–5216. DOI: 10.1021/jo034331g
4. Bruice, T. C.; Blaskd, A.; Petyak, M. E. *J. Am. Chem. Soc.* **1995**, *117*, 12064–12069. DOI: 10.1021/ja00154a005.
5. Dhawan, B.; Redmore, D. *J. Org. Chem.* **1986**, *51*, 179–183. DOI: 10.1021/jo00352a010.
6. Szabó, T.; Hirsch, E.; Tóth, T.; Müller, J.; Riethmüller, E.; Balogh, G. T.; Huszthy, P. *Tetrahedron Asymm*. **2015**, *26*, 650–656. DOI: 10.1016/j.tetasy.2015.04.015
7. The emission spectrum of the lamp used is available at https://www.ushio.com/files/specifications/low-pressure-mercury-arc-blacklight.pdf
8. Baia, L.; Wang, J.-X. *Adv. Synth. Catal.* **2008**, *350*, 315–320. DOI:10.1002/adsc.200700361.
9. Bandari, R.; Höche, T.; Prager, A.; Dirnberger, K.; Buchmeiser, M. R. *Chem. Eur. J.* **2010**, *16*, 4650–4658. DOI: 10.1002/chem.200902654.
10. Lohre, C.; Dröge, T.; Wang, C.; Glorius, F. *Chem. Eur. J.* **2011,** *17*, 6052–6055. DOI: 10.1002/chem.201100909.
11. Qrareya, H.; Raviola, C.; Protti,S.; Fagnoni, M.; Albini, A. *J. Org. Chem*. **2013**, *78*, 6016–6024. DOI: 10.1021/jo4007046.
12. McKinley, N. F.; O'Shea, D. F. *J. Org. Chem.* **2004**, *69*, 5087–5092. DOI: 10.1021/jo4007046.
13. Chen, Q.; Wu, S.; Yan, S.; Li, C.; Abduhulam, H.; Shi, Y.; Dang, Y.; Cao, C. *ACS Catal.* **2020**, *10*, 8168−8176. DOI: 10.1021/acscatal.0c01462.
14. Kuninobu, Y.; Nishi, M.; Kawata, A.; Takata, H.; Hanatani, Y.; Yudha, S.; Iwai, A.; Takai, K. *J. Org. Chem.* **2010**, *75*, 334–341. DOI: 10.1021/jo902072q.
15. Zhang, Y.; Zhang, R.; Ni, C.; Zhang, X.; Li, Y.; Lu, Q.; Zhao, Y.; Han, F.; Zeng, Y.; Liu, G. *Tetrahedron Lett.* **2020**, *61*, 151541. DOI: 10.1016/j.tetlet.2019.151541.
16. Cepanec, I.; Litvić, M.; Udiković, J.; Pogorelić, I.; Lovric, M. *Tetrahedron* **2007**, *63*, 5614–5621. DOI: 10.1016/j.tet.2007.04.016
17. Qu, X.; Li, T.; Zhu, Y.; Sun, P.; Yang, H.; Mao, J. *Org. Biomol. Chem*. **2011**, *9*, 5043–5046, DOI: 10.1039/C1OB05155E.

18. Cheng, J.; Tang, L.; Xu, J. *Adv. Synth. Catal.* **2010**, *352*, 3275–3286. DOI: 10.1002/adsc.201000475.
19. Cahiez, G.; Chaboche, C.; Mahuteau-Betzer, F.; Ahr, M. *Org. Lett.* **2005**, *7*, 1943-1946. DOI: 10.1021/ol050340v.

4. Copy of the ^{1}H and ^{13}C NMR of compounds 1a–l, 2a, 2c–l, 3a–e, 4b–d

4-Chlorophenyl diphenyl phosphate (1a). (^{1}H NMR 300 MHz, CD_3COCD_3)

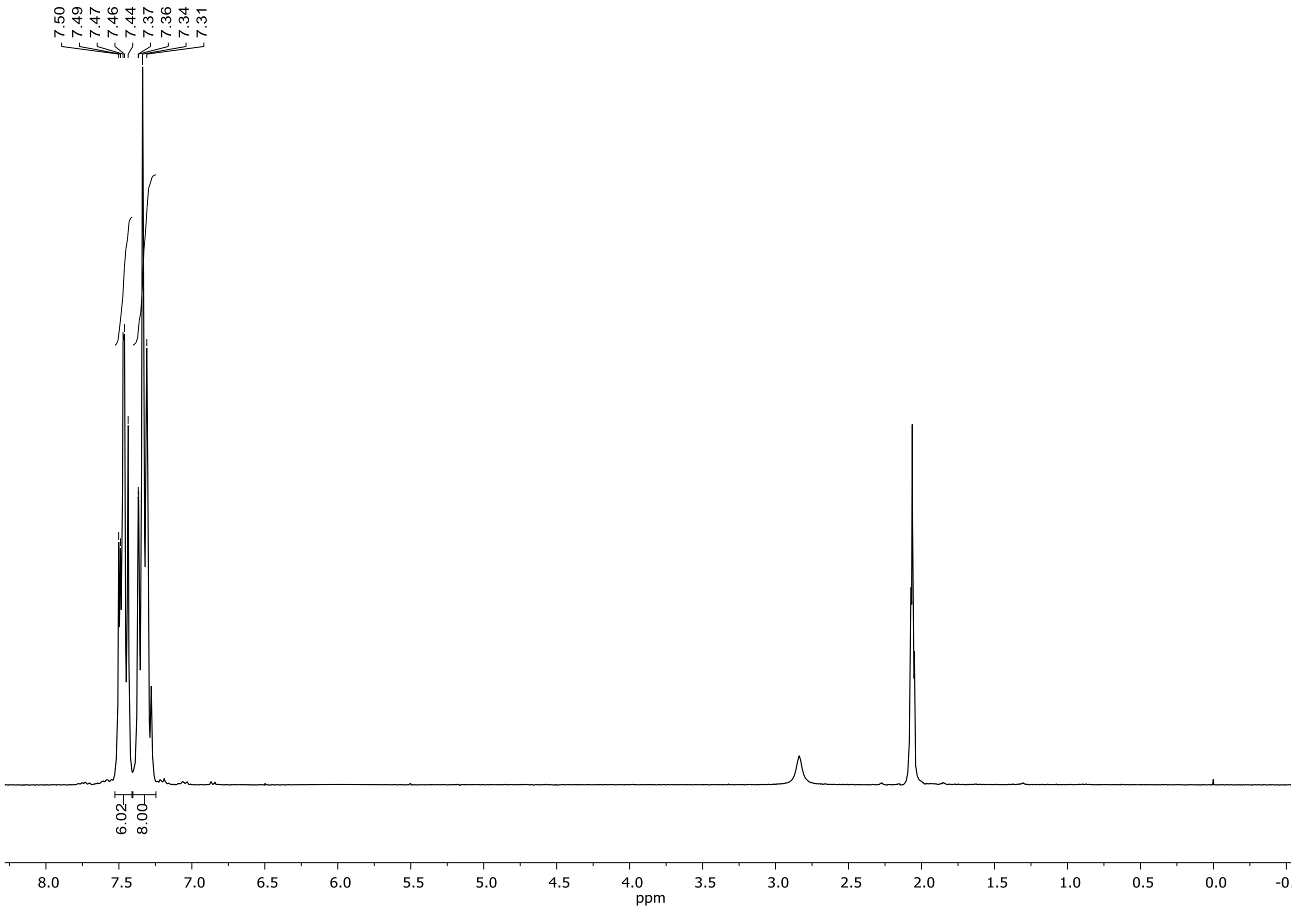

4-Chlorophenyl diphenyl phosphate (1a). (^{13}C NMR 75 MHz, CD_3COCD_3)

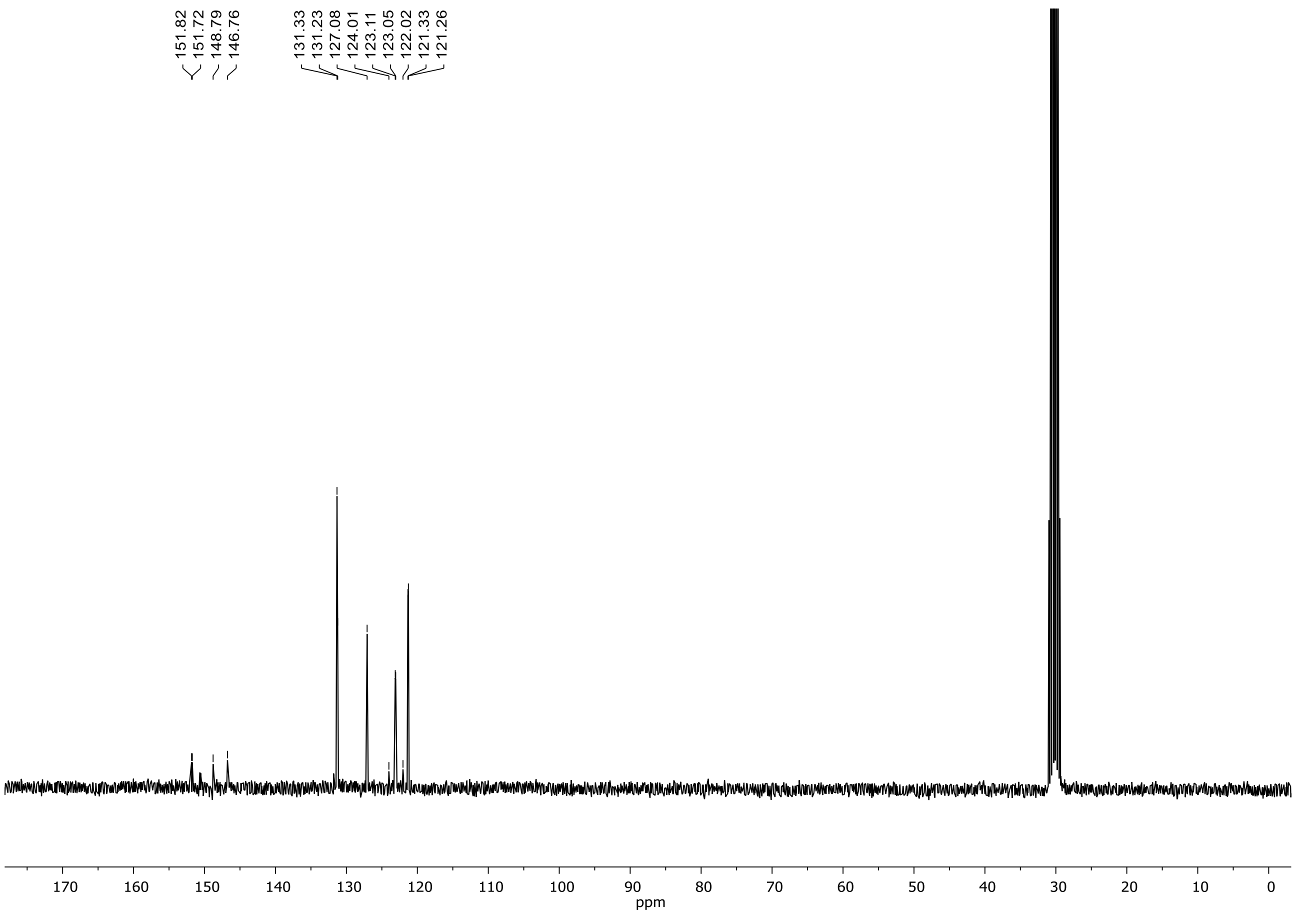

Triphenyl phosphate (1b). (^{1}H NMR 300 MHz, CD_3COCD_3)

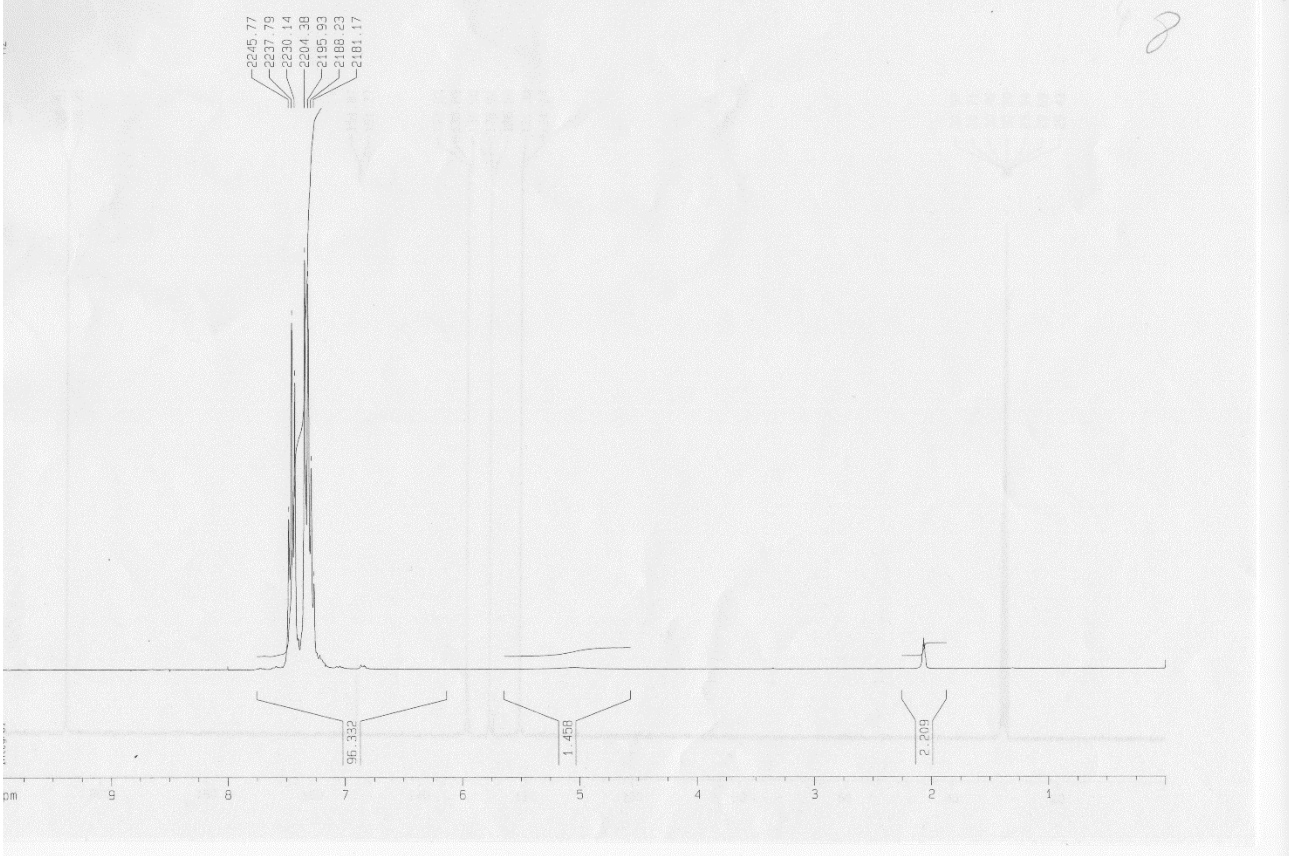

Triphenyl phosphate (1b). (^{13}C NMR 75 MHz, CD_3COCD_3)

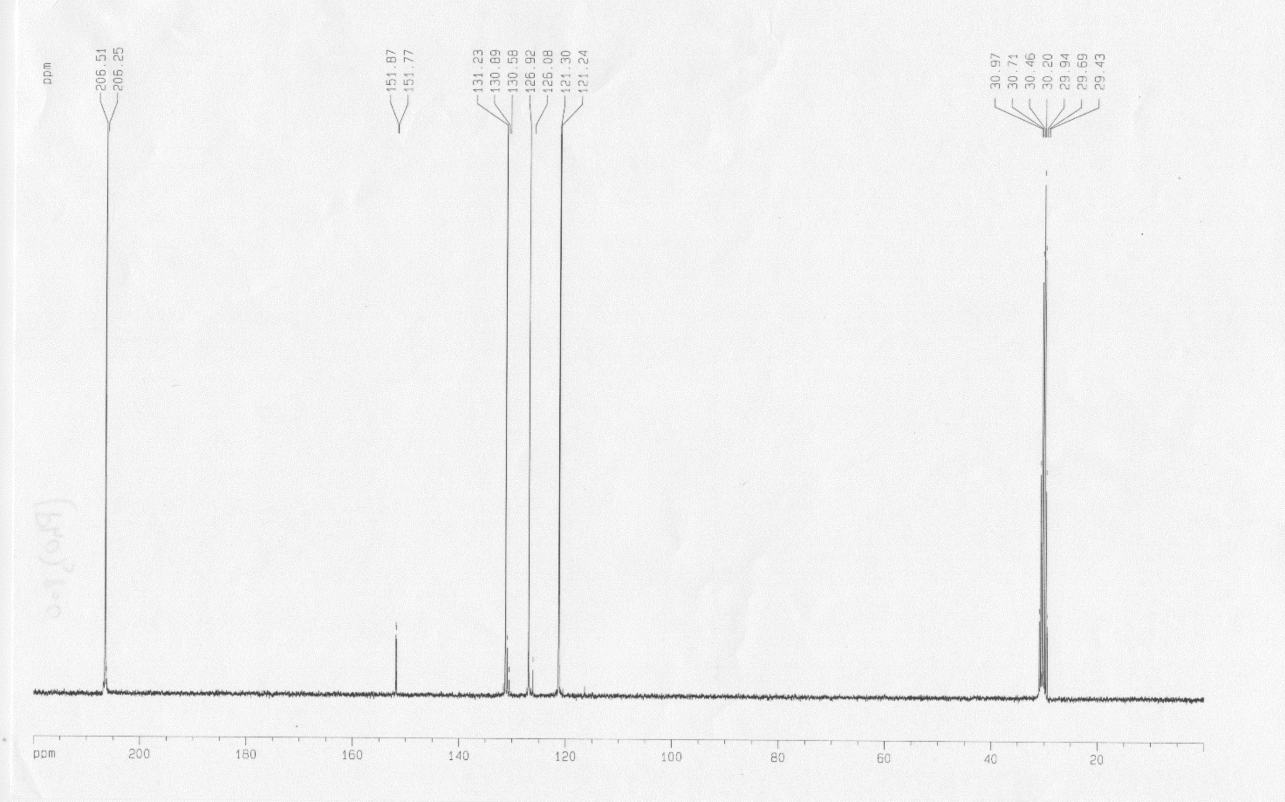

4-Methylphenyl diphenyl phosphate (1c). (^{1}H NMR 300 MHz, CD_3COCD_3)

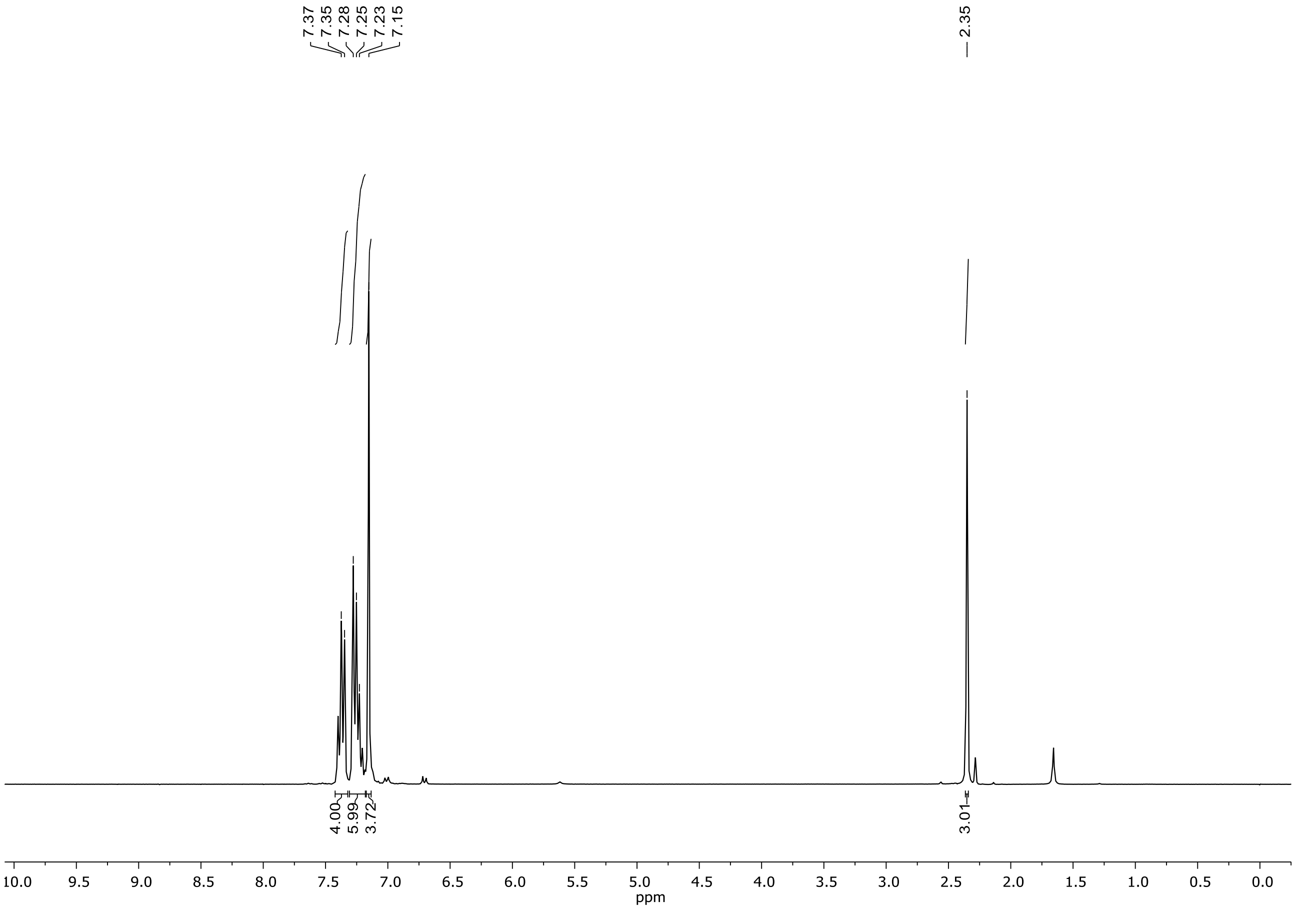

4-Methylphenyl diphenyl phosphate (1c). (^{13}C NMR 75 MHz, CD_3COCD_3)

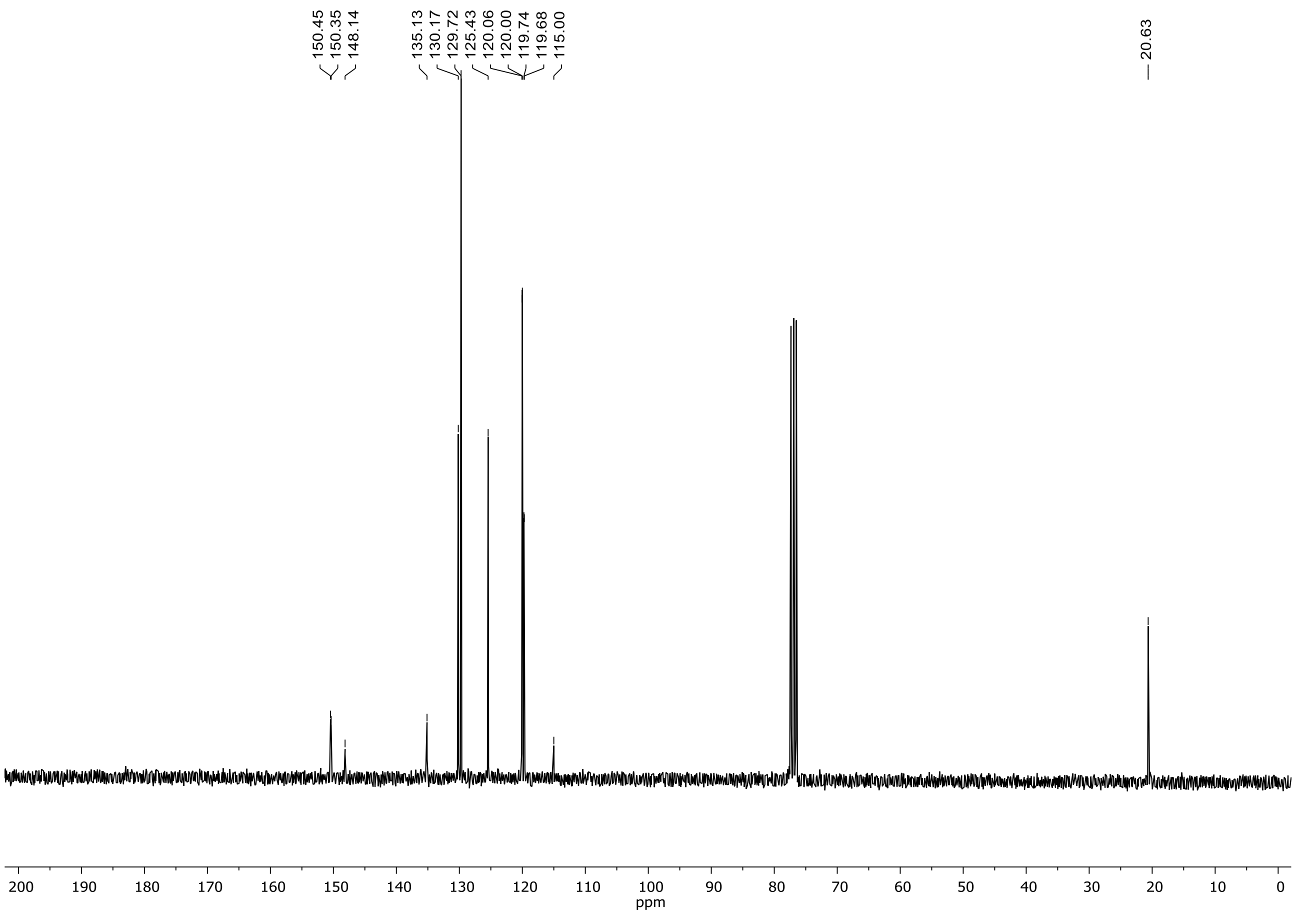

4-Isopropylphenyl diphenyl phosphate (1d). (^{1}H NMR 300 MHz, $CDCl_3$)

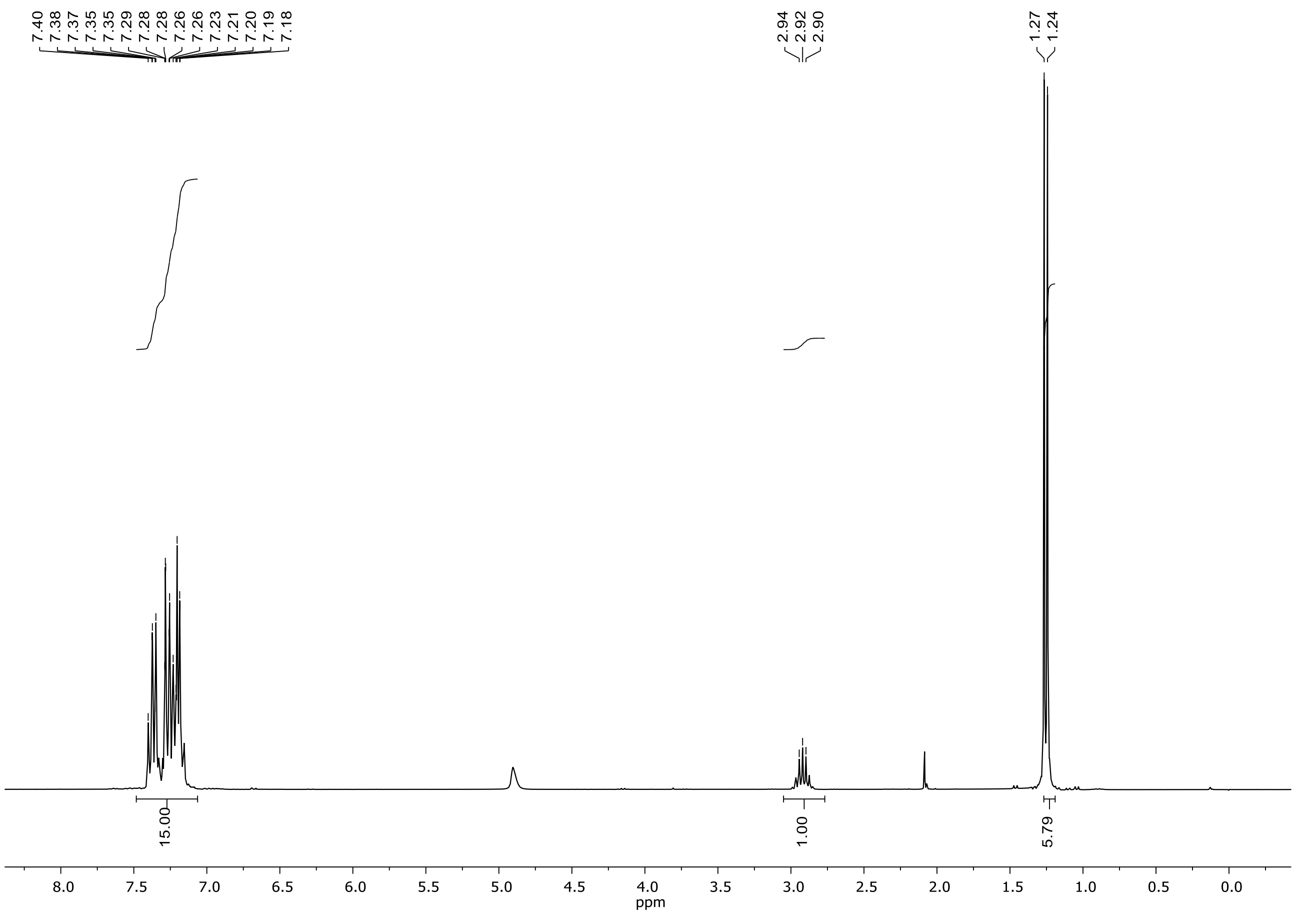

4-Isopropylphenyl diphenyl phosphate (1d). (^{13}C NMR 75 MHz, $CDCl_3$)

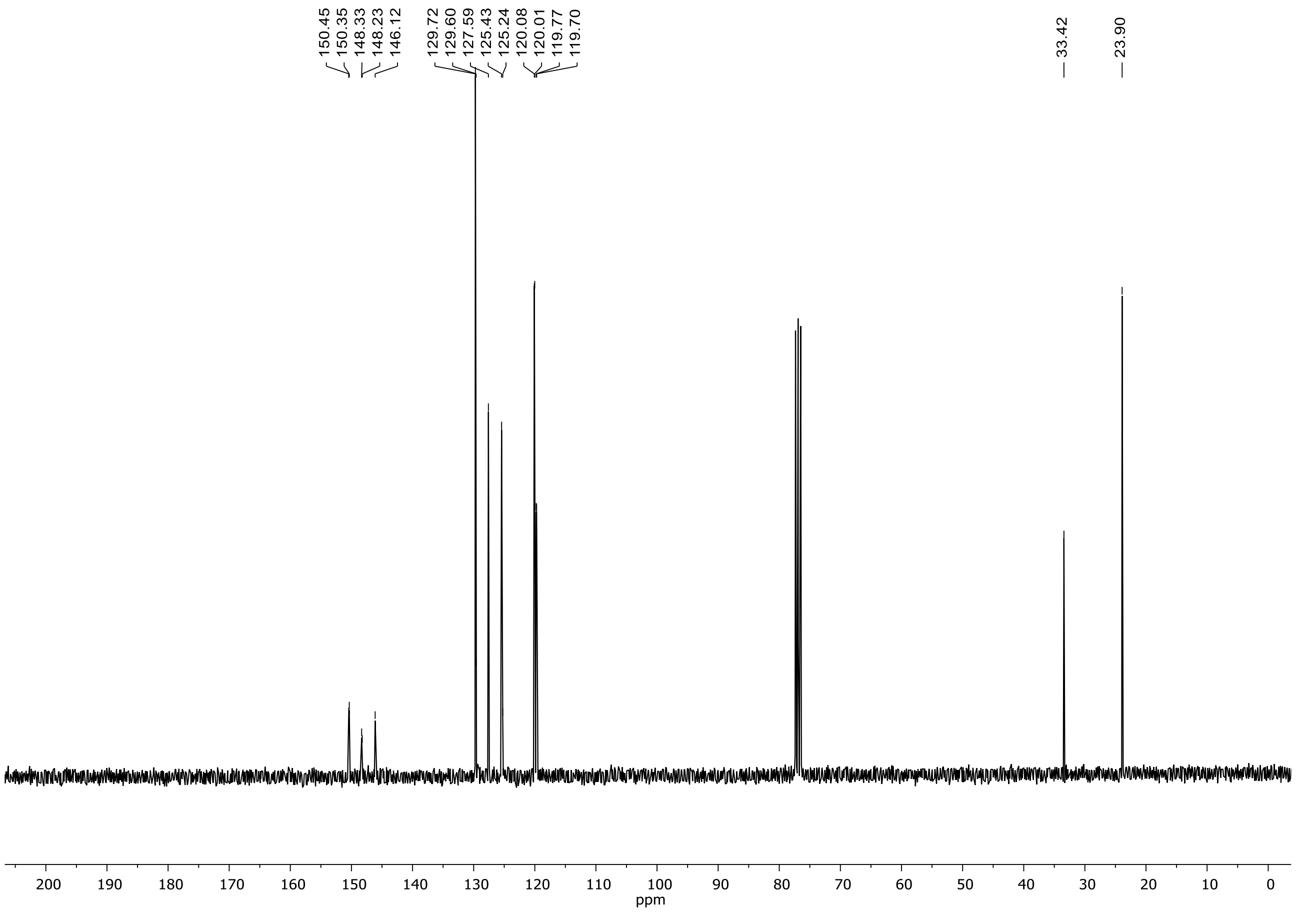

4-*tert*-Butyl-phenyl diphenyl phosphate (1e). (^{1}H NMR 300 MHz, $CDCl_3$)

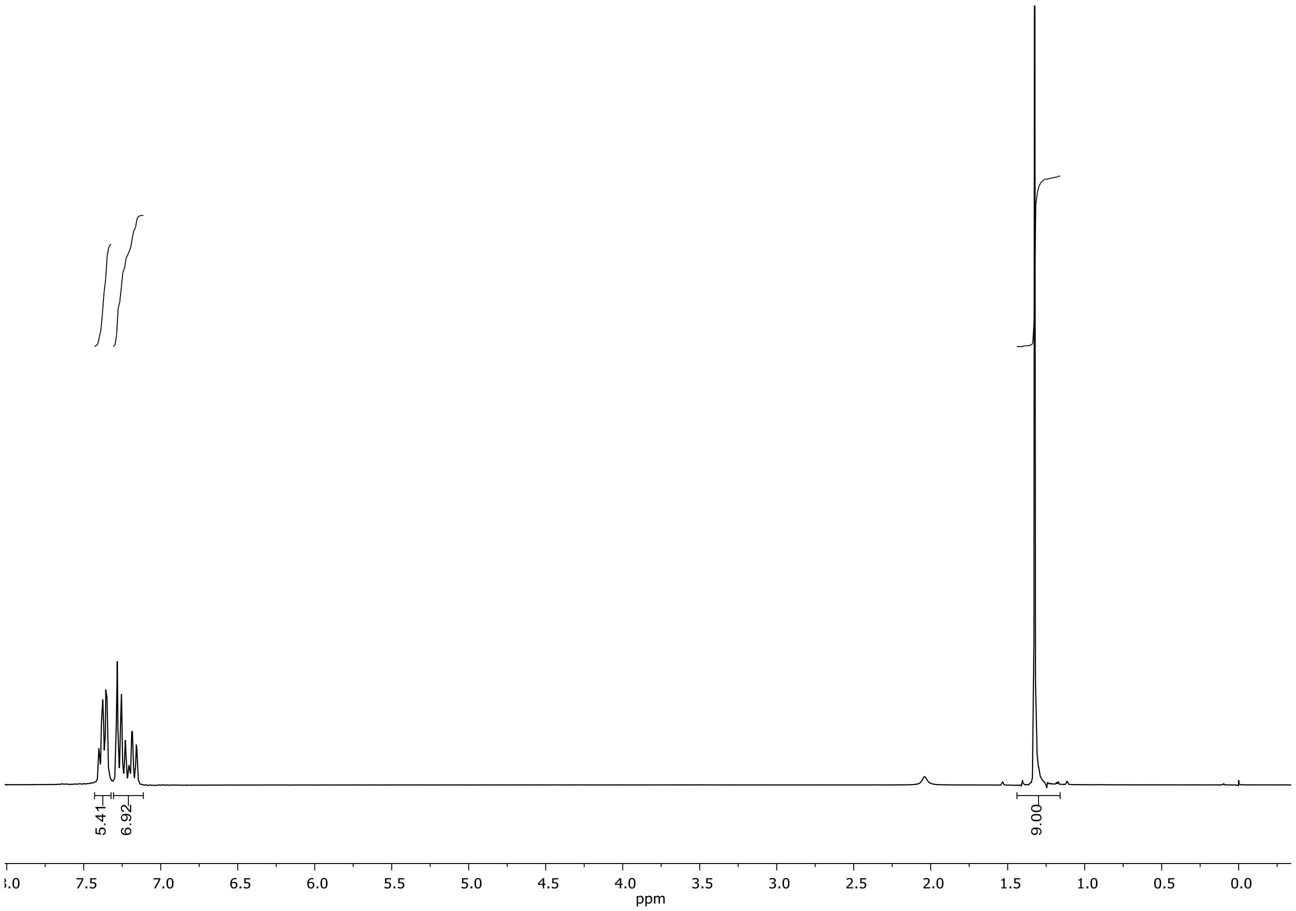

4-*tert*-Butyl-phenyl diphenyl phosphate (1e). (^{13}C NMR 75 MHz, $CDCl_3$).

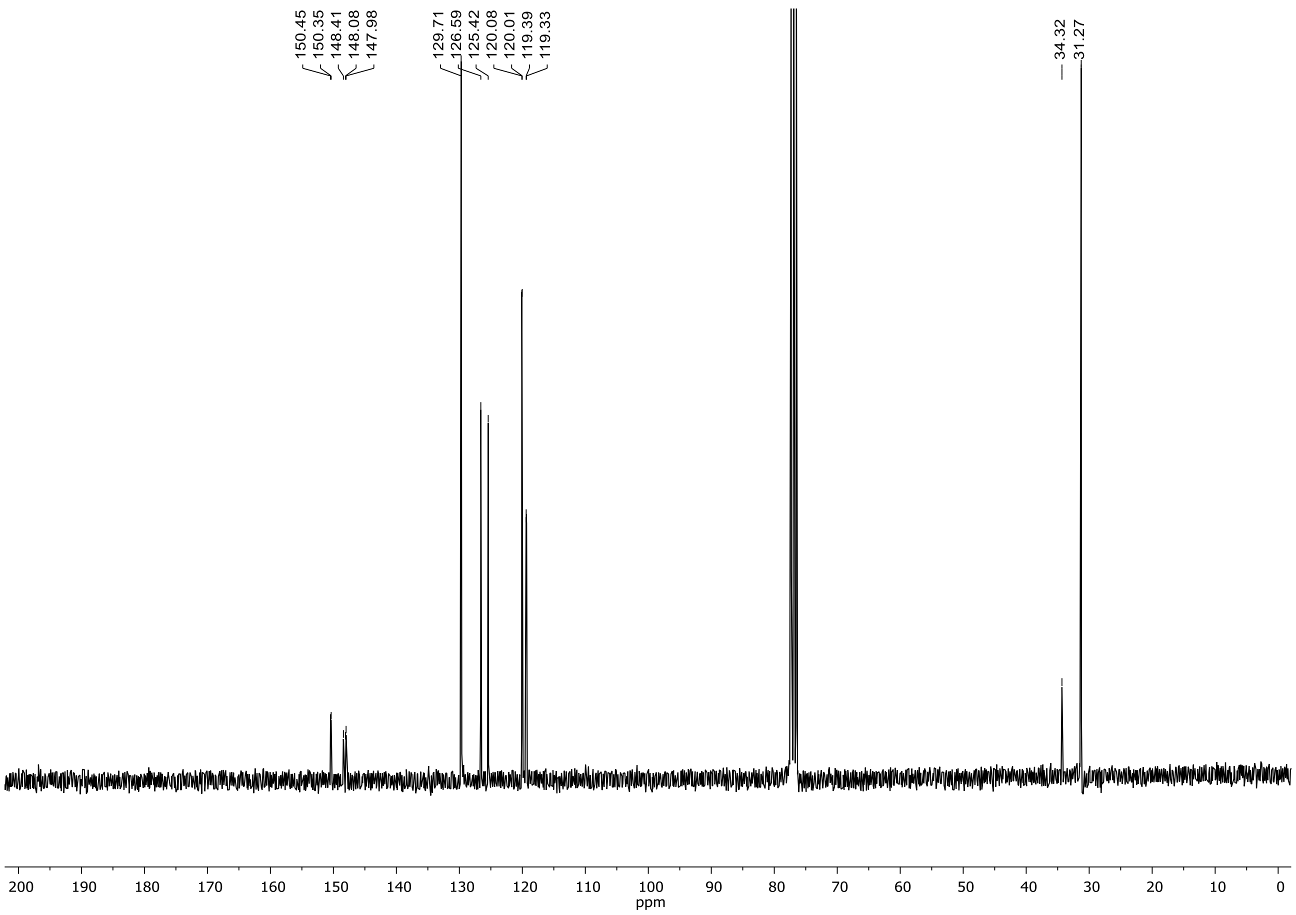

4-Methoxyphenyl diphenyl phosphate (1f). (^{1}H NMR 300 MHz, $CDCl_3$)

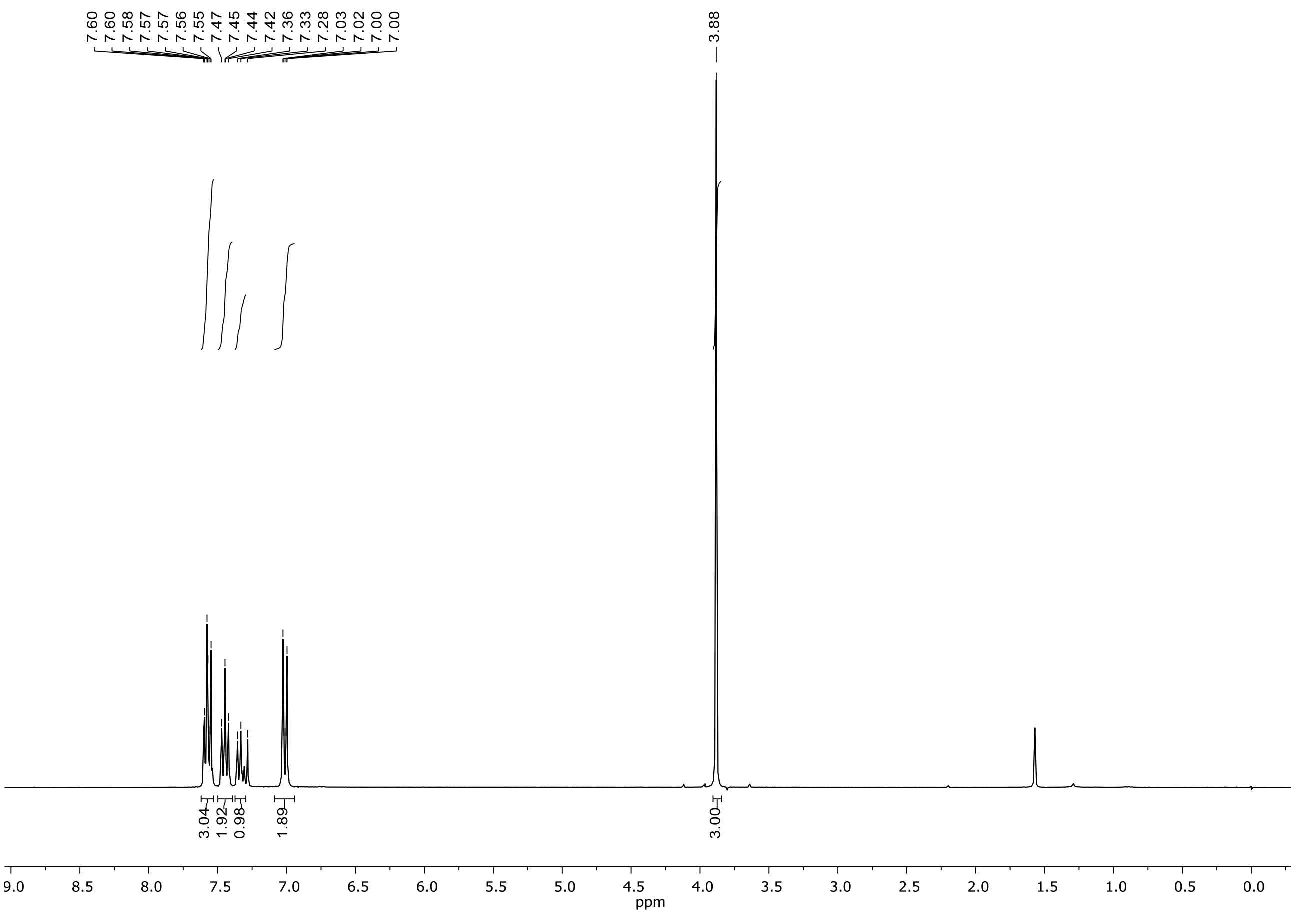

4-Methoxyphenyl diphenyl phosphate (1f). (^{13}C NMR 75 MHz, $CDCl_3$)

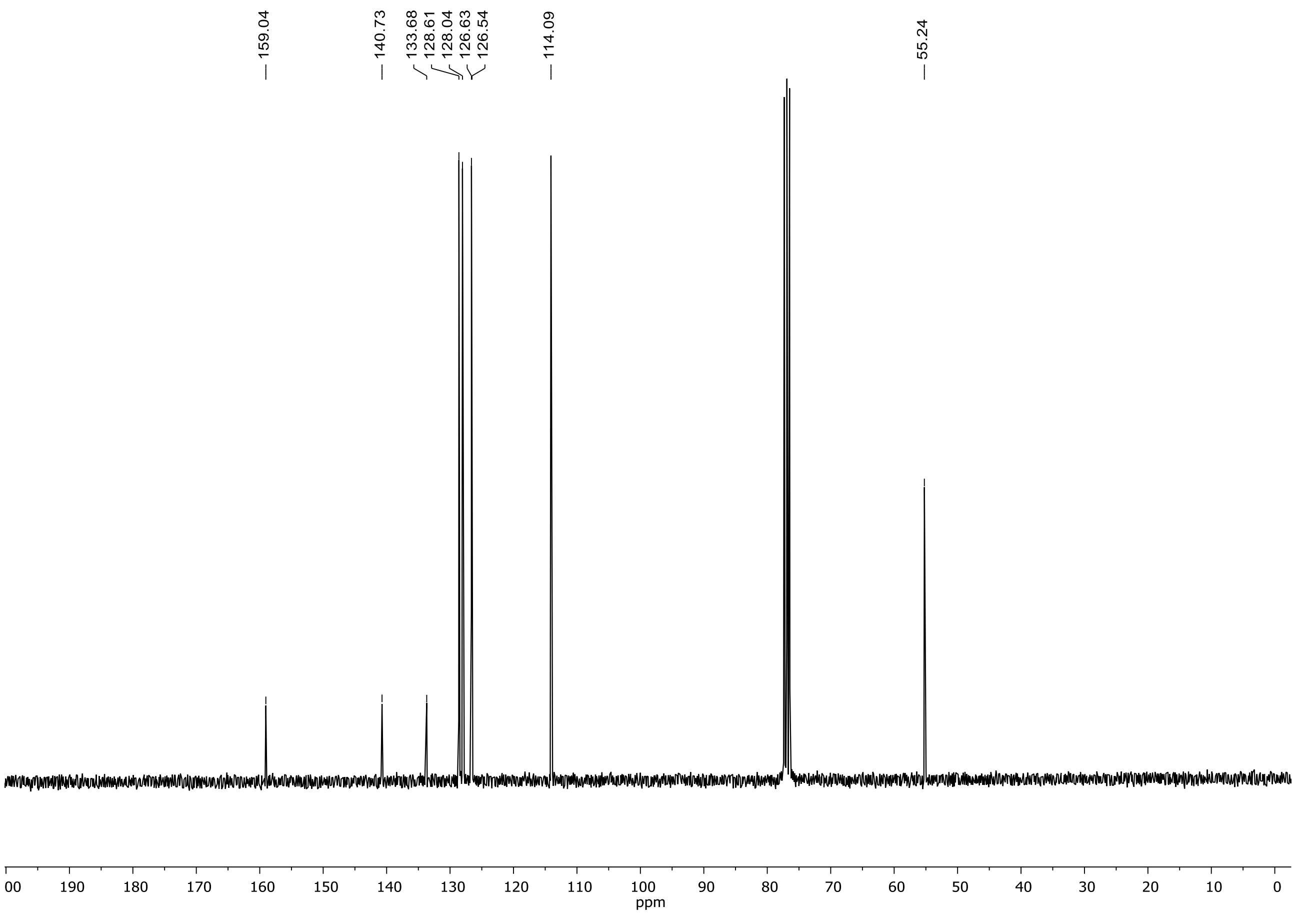

4-Phenoxy-phenyl diphenyl phosphate (1g). (^{1}H NMR 300 MHz, $CDCl_3$)

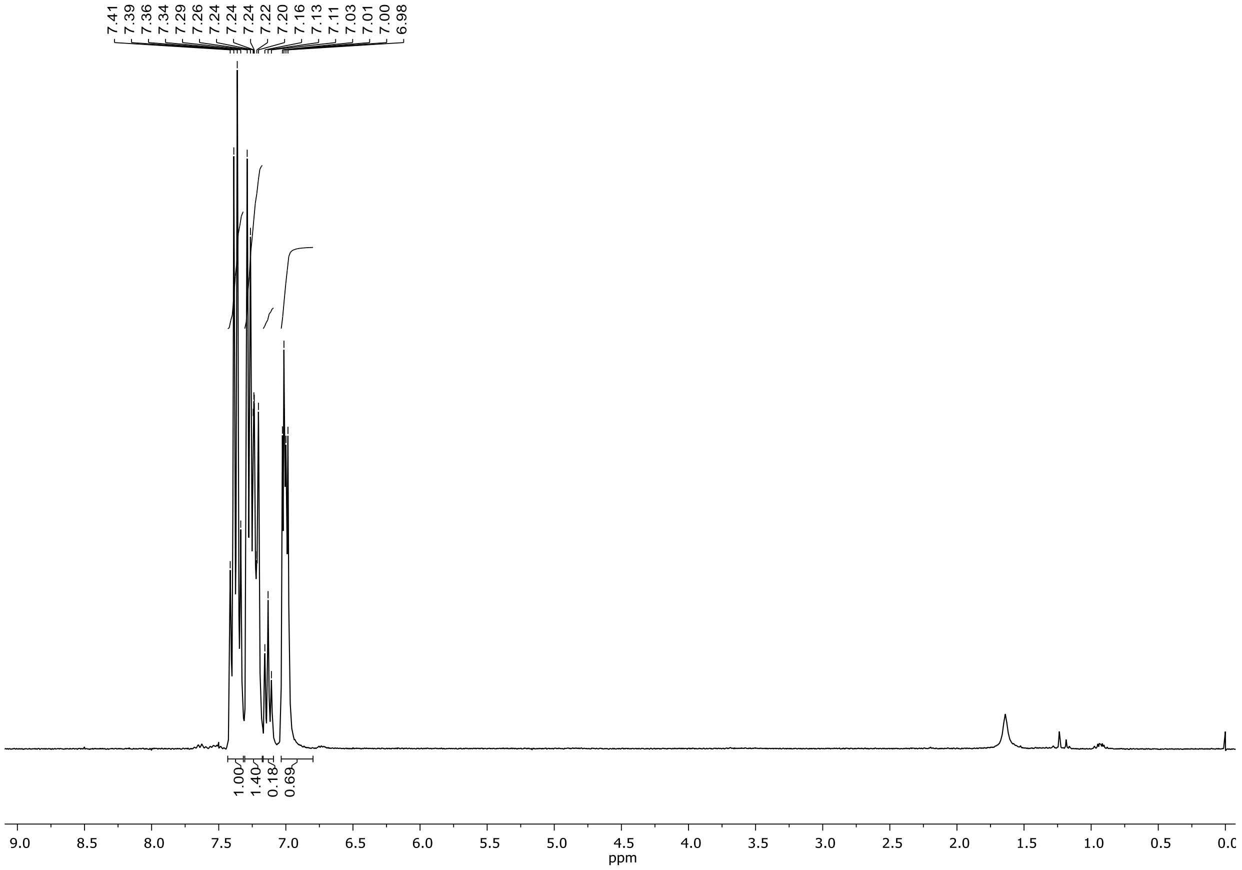

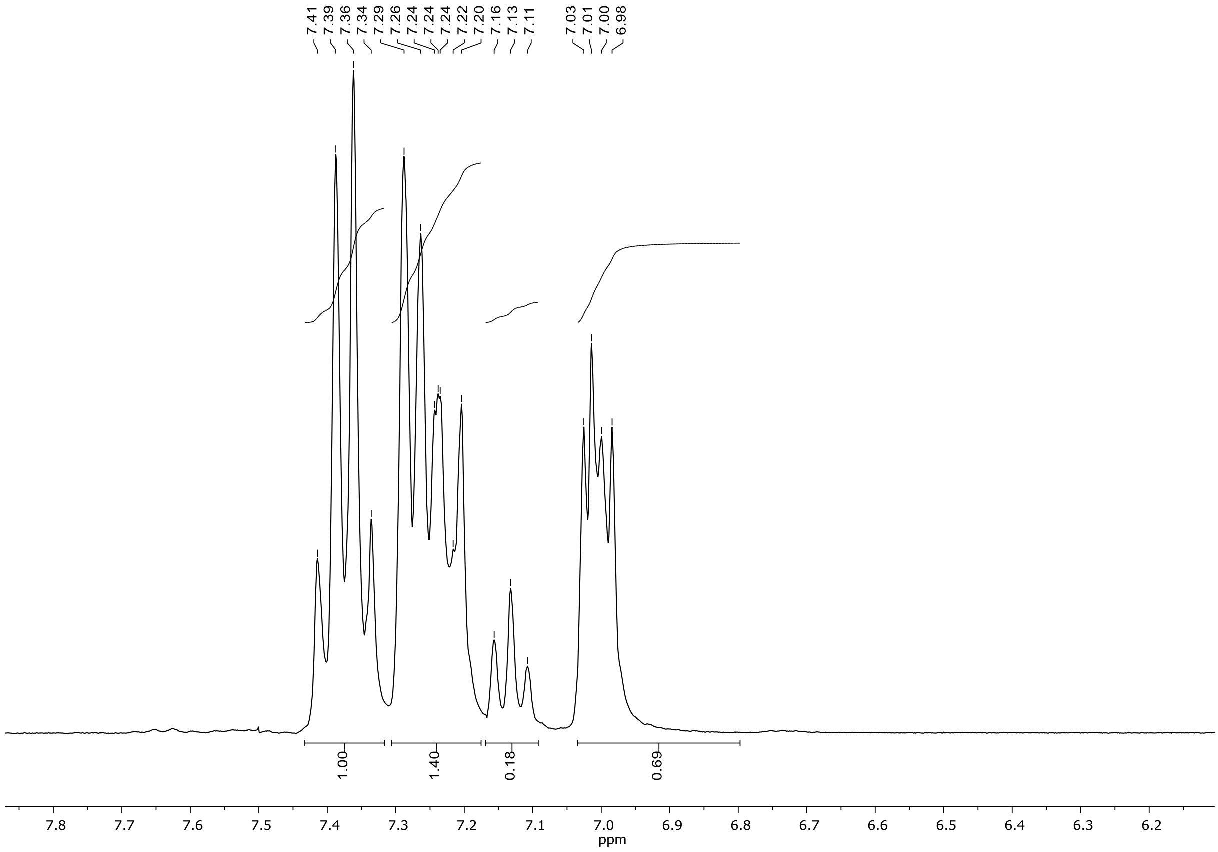

4-Phenoxy-phenyl diphenyl phosphate (1g). (^{13}C NMR 75 MHz, $CDCl_3$)

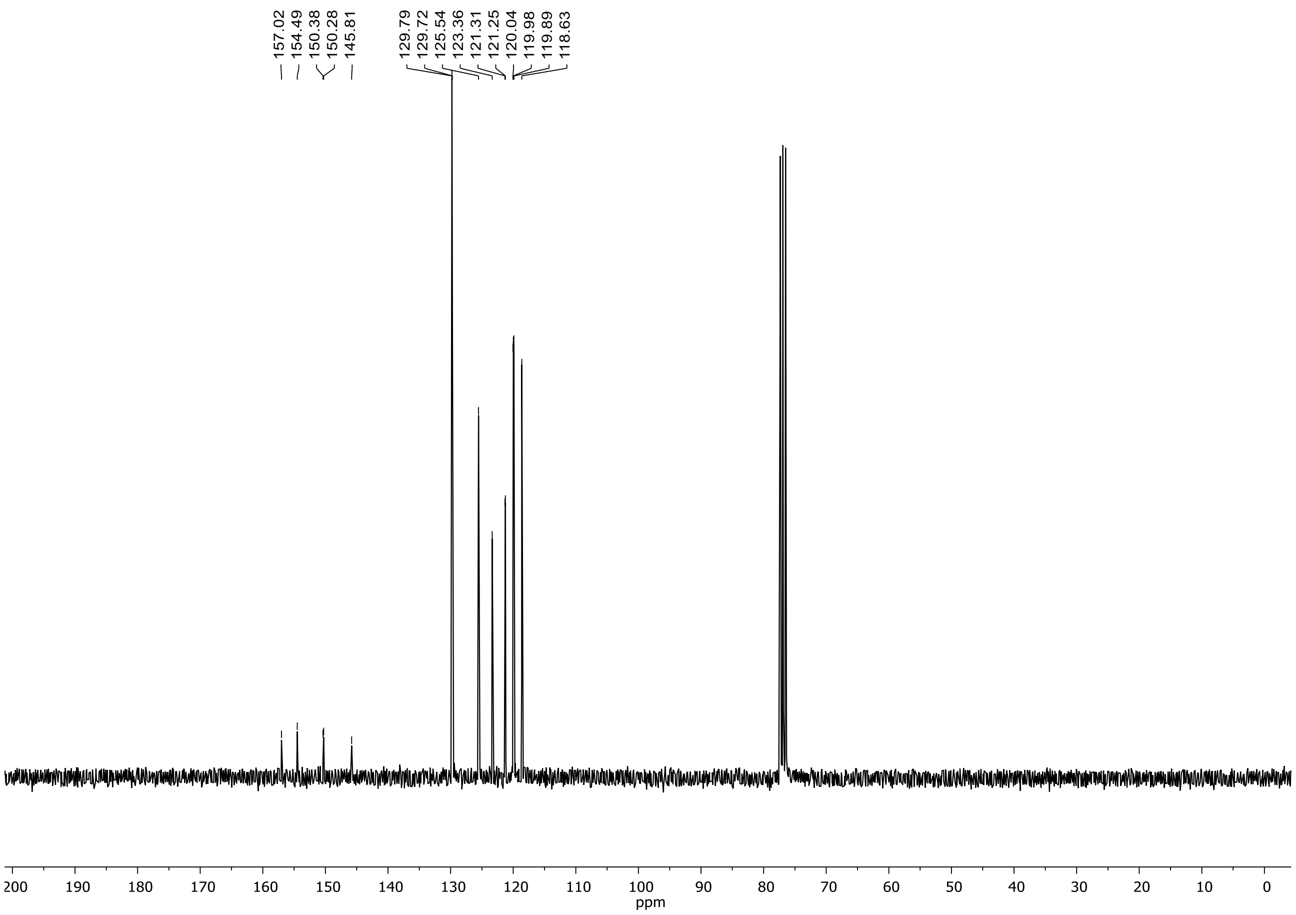

4-Cyanophenyl diphenyl phosphate (1h). (^{1}H NMR 300 MHz, $CDCl_3$)

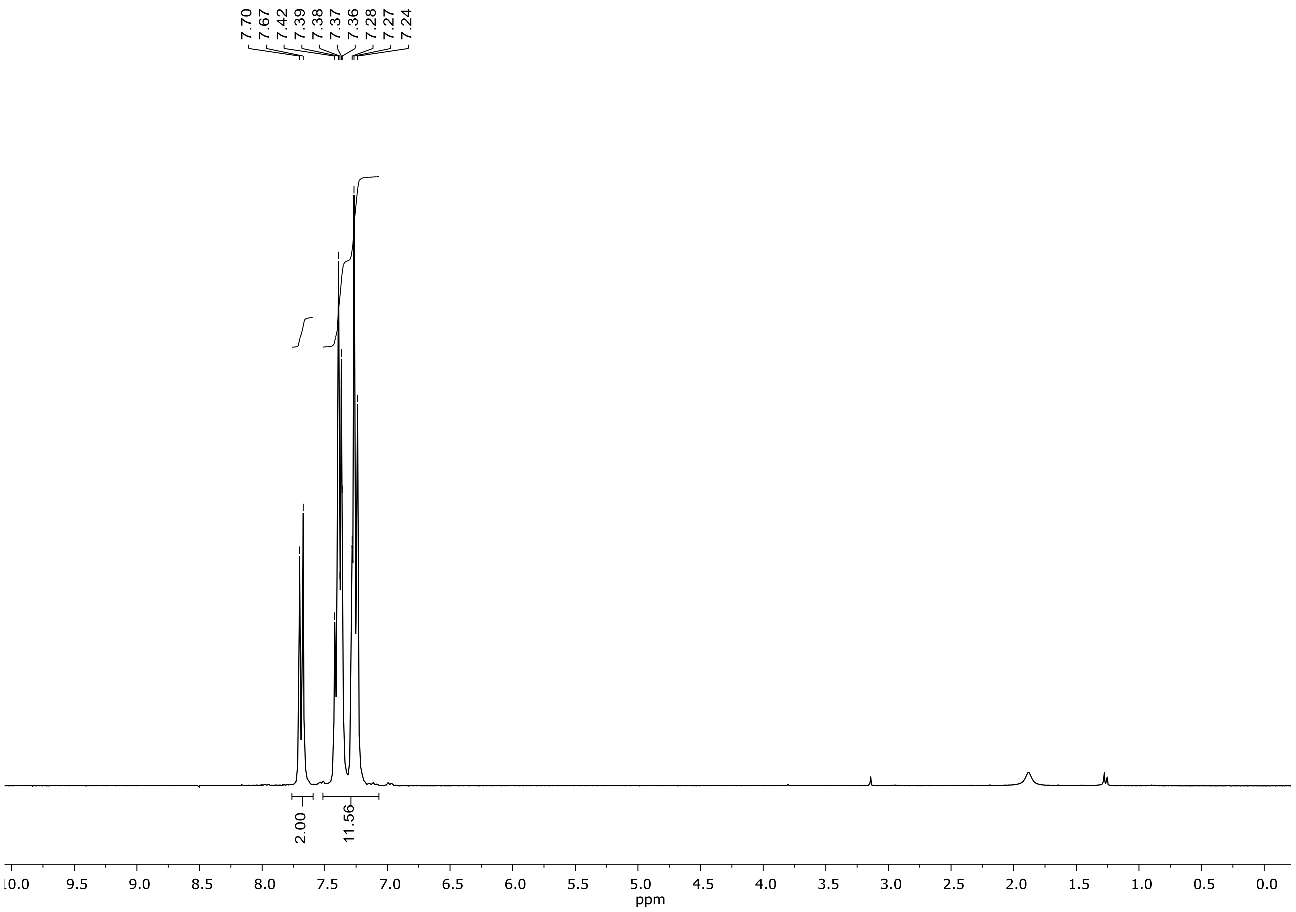

4-Cyanophenyl diphenyl phosphate (1h). (^{13}C NMR 75 MHz, $CDCl_3$)

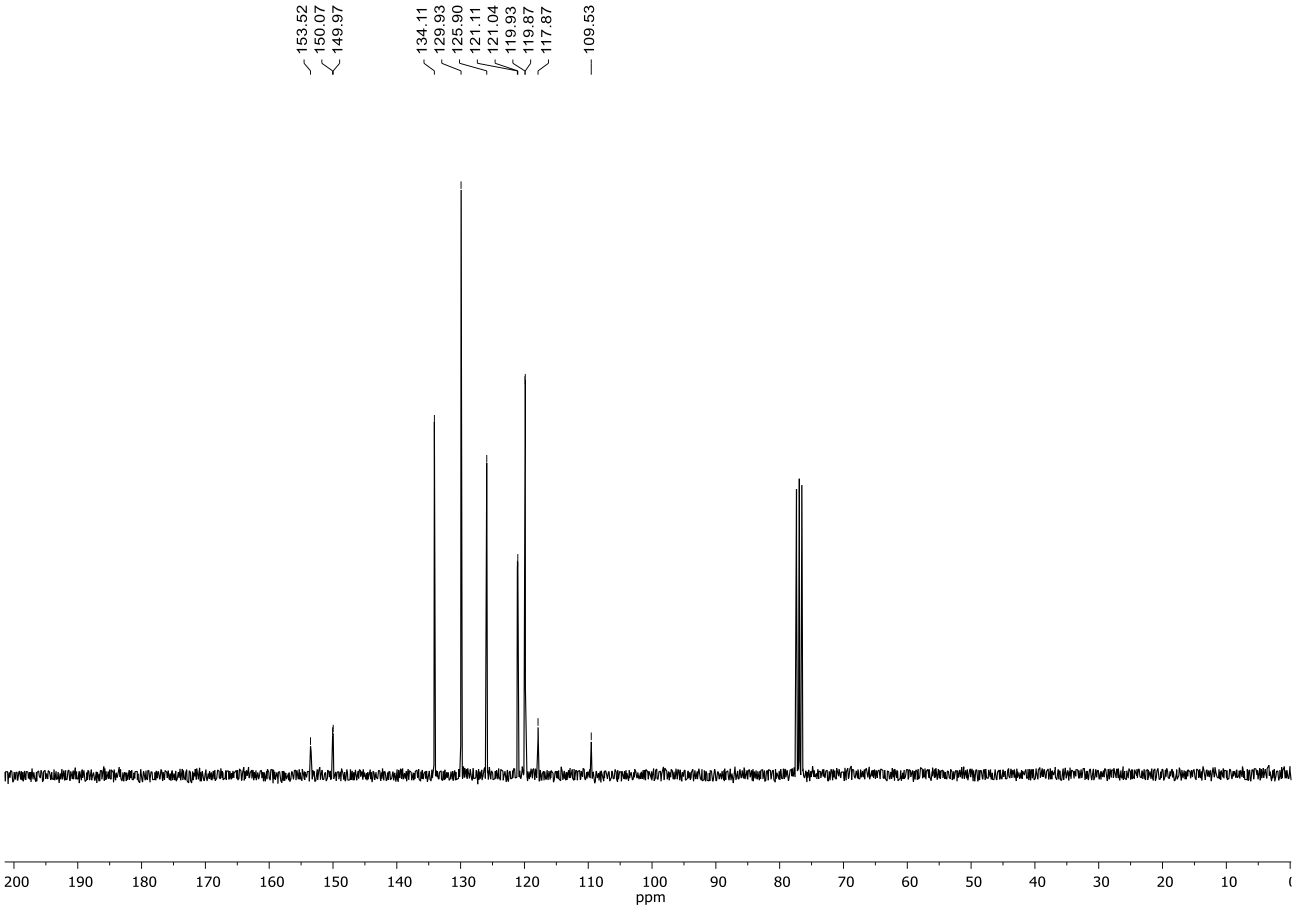

3-Methoxyphenyl diphenyl phosphate (1i). (^{1}H NMR 300 MHz, $CDCl_3$)

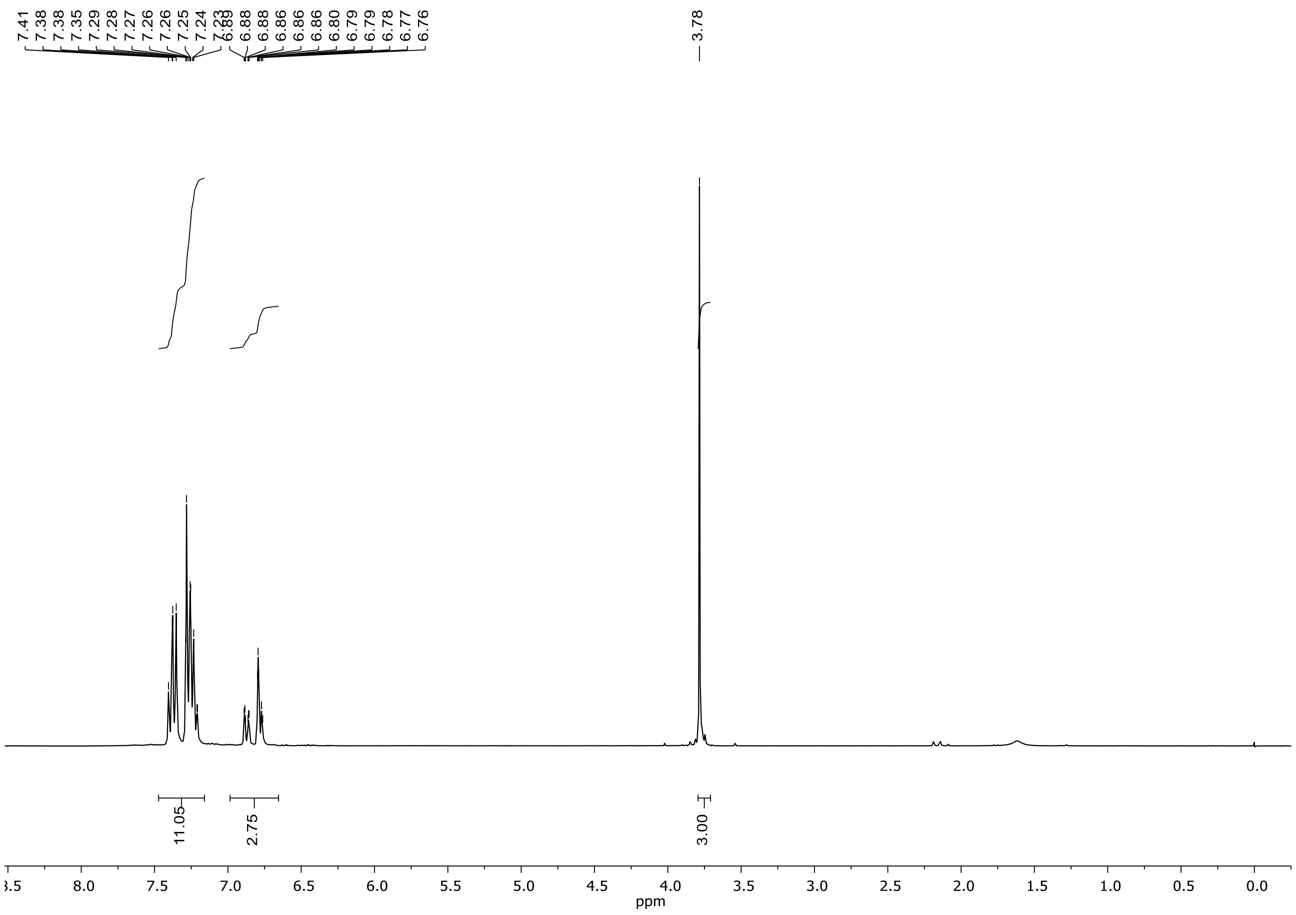

3-Methoxyphenyl diphenyl phosphate (1i). (^{13}C NMR 75 MHz, $CDCl_3$)

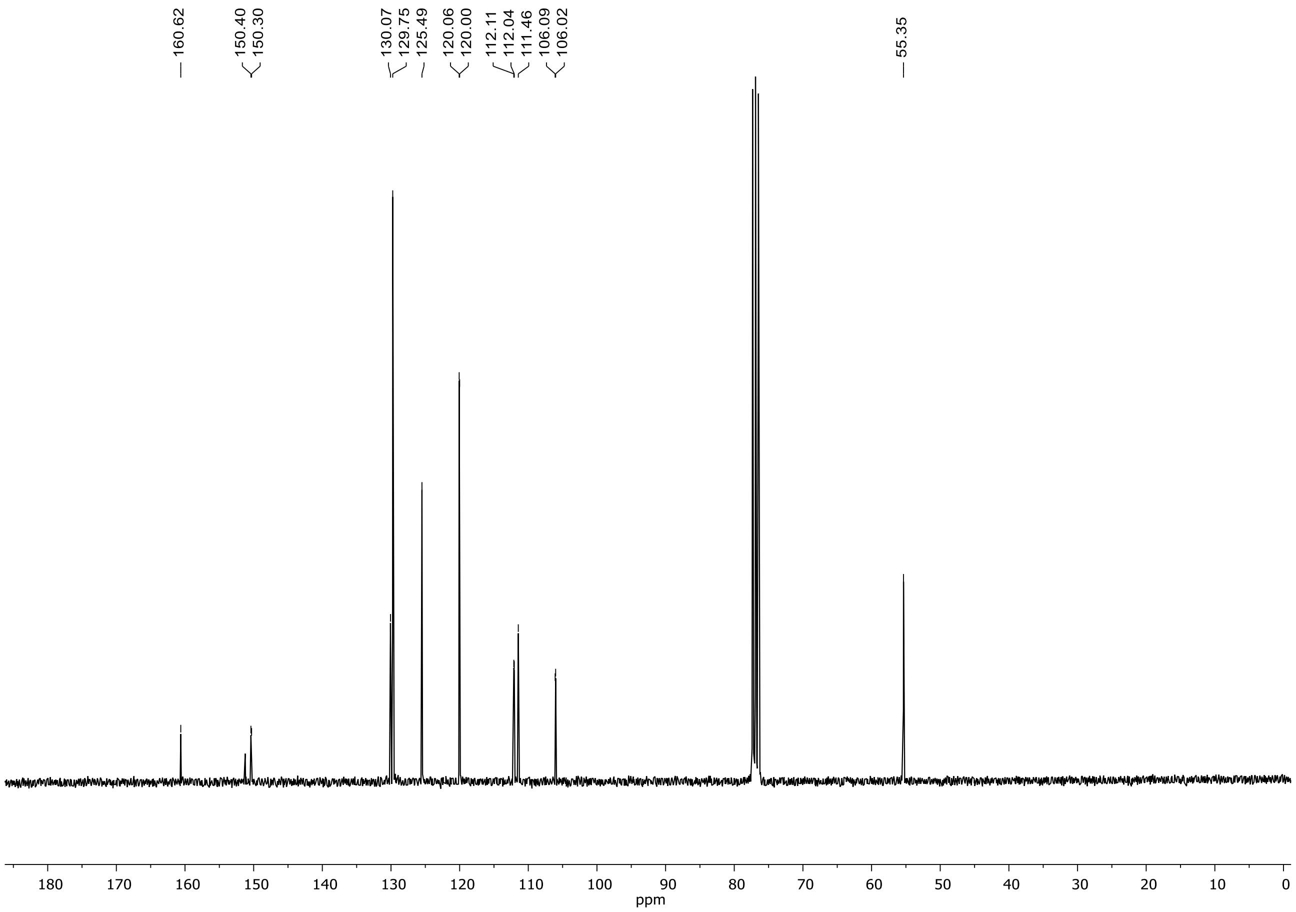

3,5-Dimethylphenyl diphenyl phosphate (1j). (^{1}H NMR 300 MHz, $CDCl_3$)

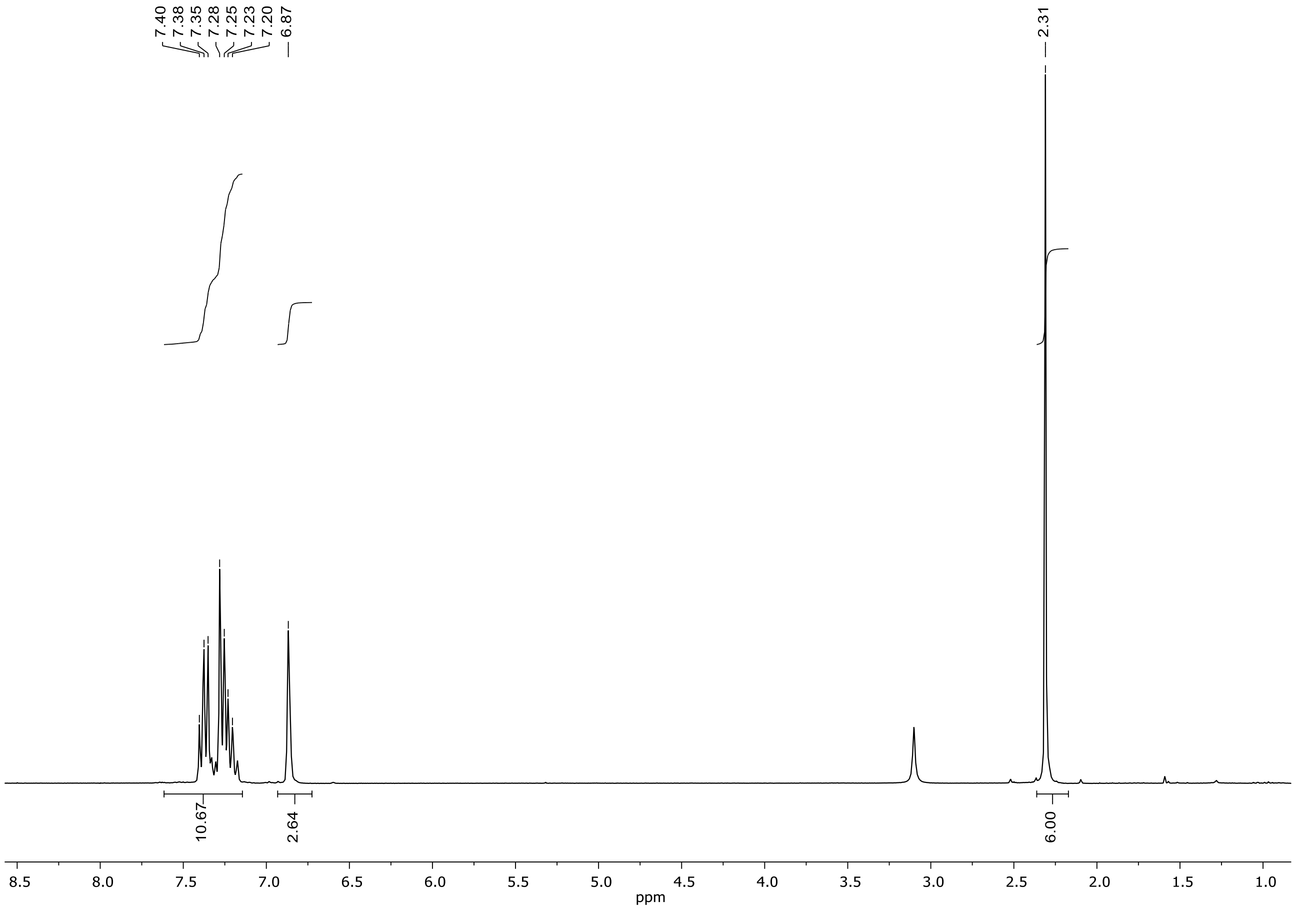

3,5-Dimethylphenyl diphenyl phosphate (1j). (^{13}C NMR 75 MHz, $CDCl_3$)

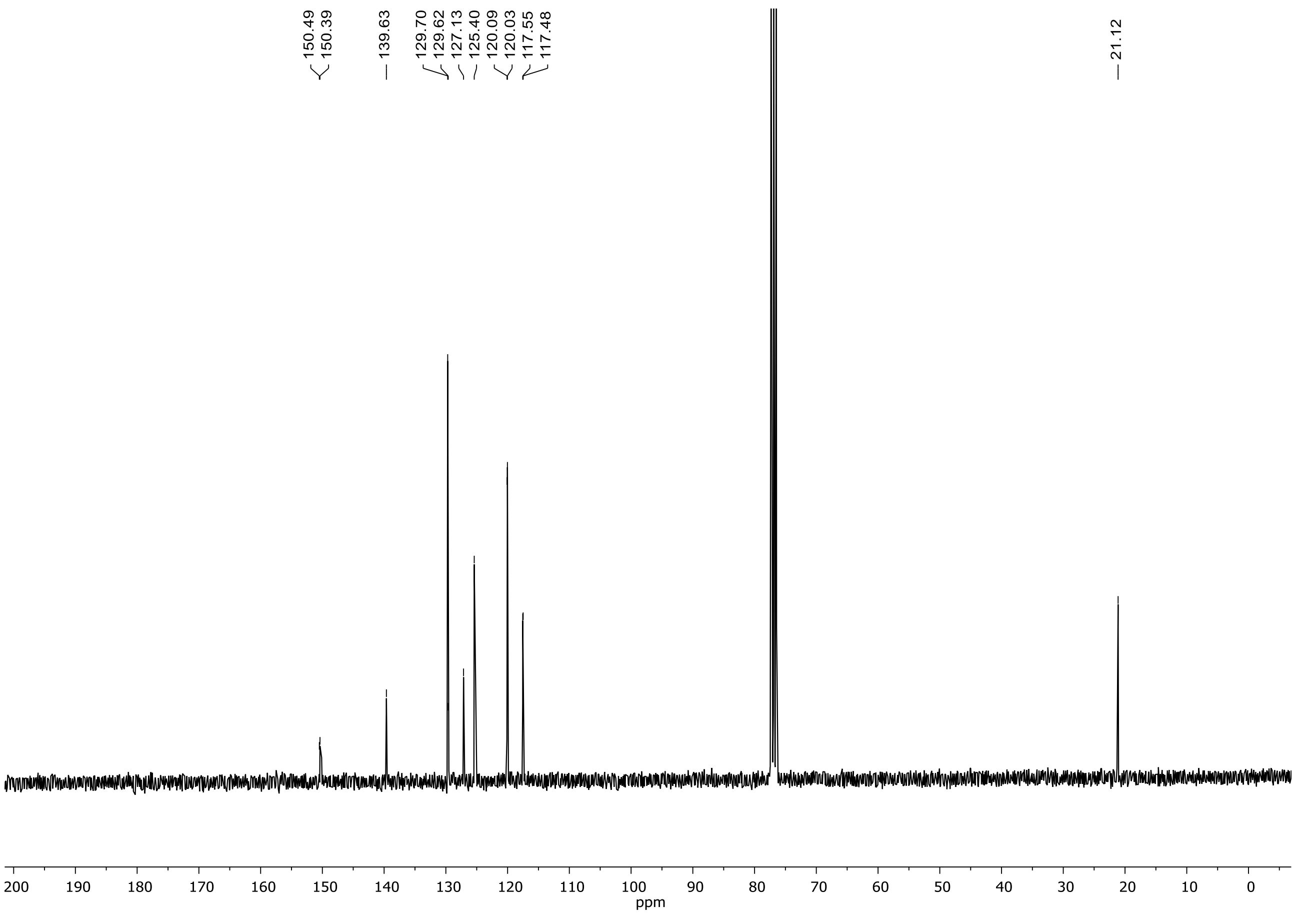

2,3,5-Trimethylphenyl diphenyl phosphate (1k). (^1H NMR 300 MHz, $CDCl_3$)

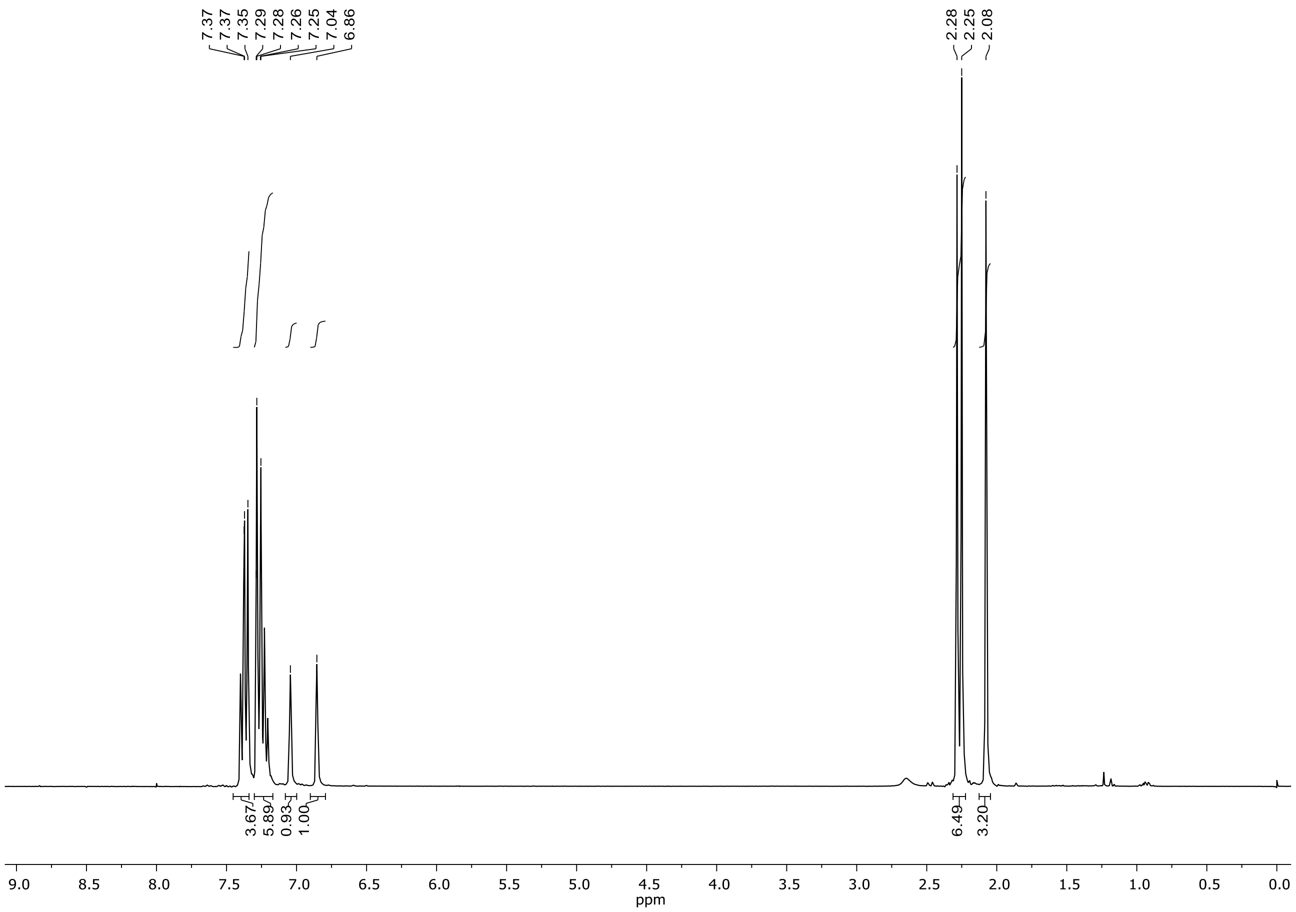

2,3,5-Trimethylphenyl diphenyl phosphate (1k). (^{13}C NMR 75 MHz, $CDCl_3$)

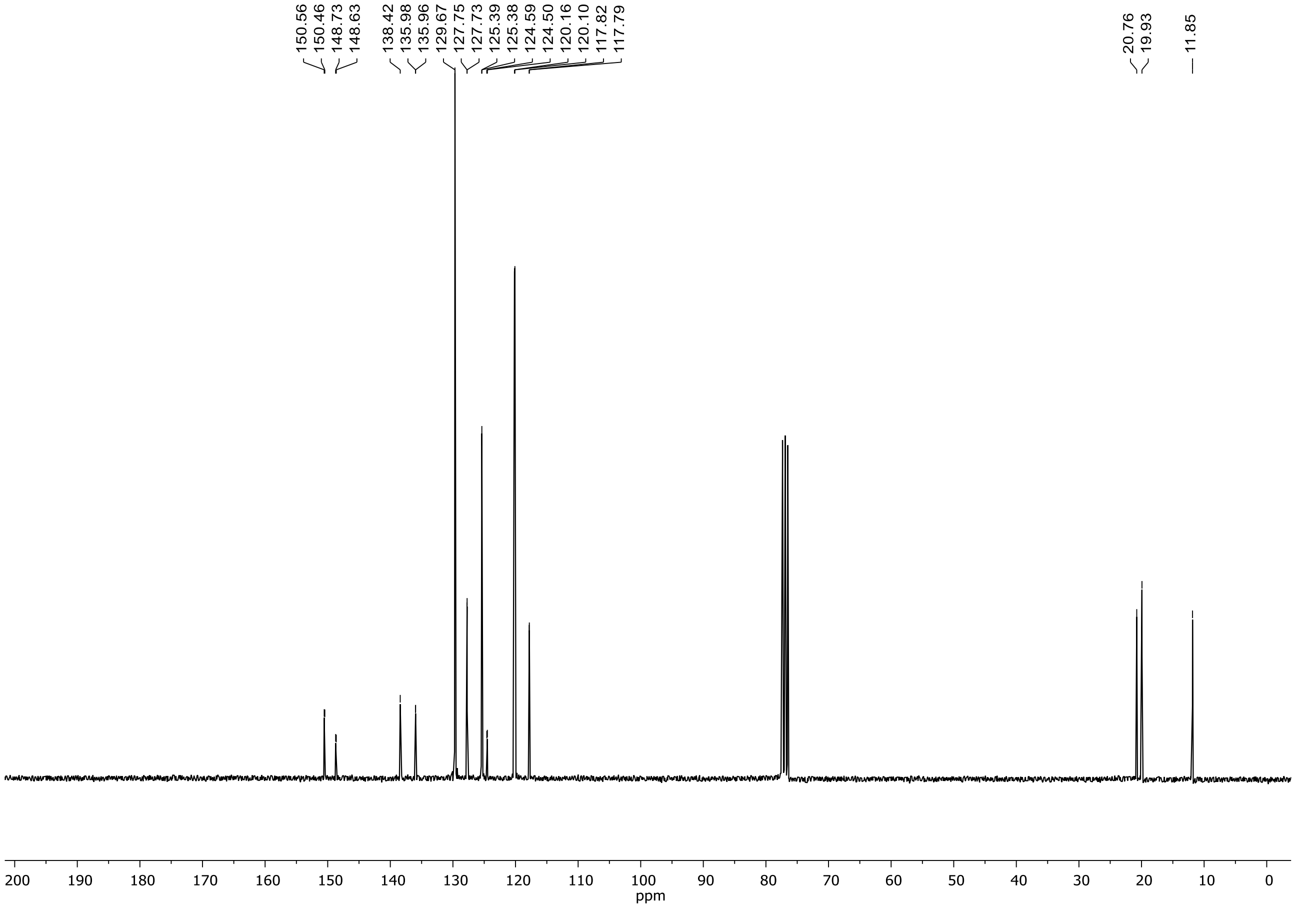

3,5-Dimethyl-4-chlorophenyl diphenyl phosphate (1l). (^{1}H NMR 300 Hz, $CDCl_3$)

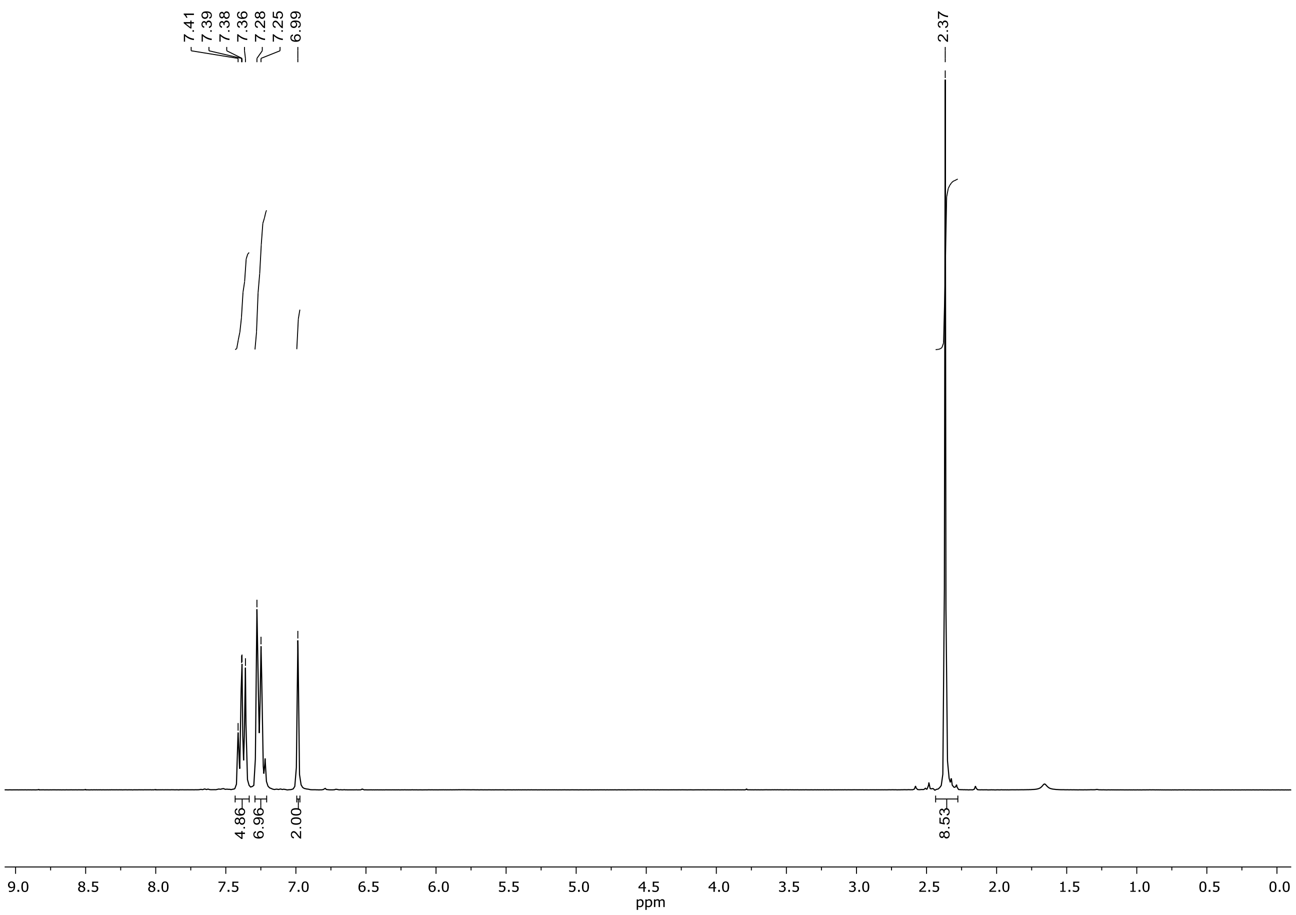

3,5-Dimethyl-4-chlorophenyl diphenyl phosphate (1l). (^{13}C NMR 75 Hz, $CDCl_3$)

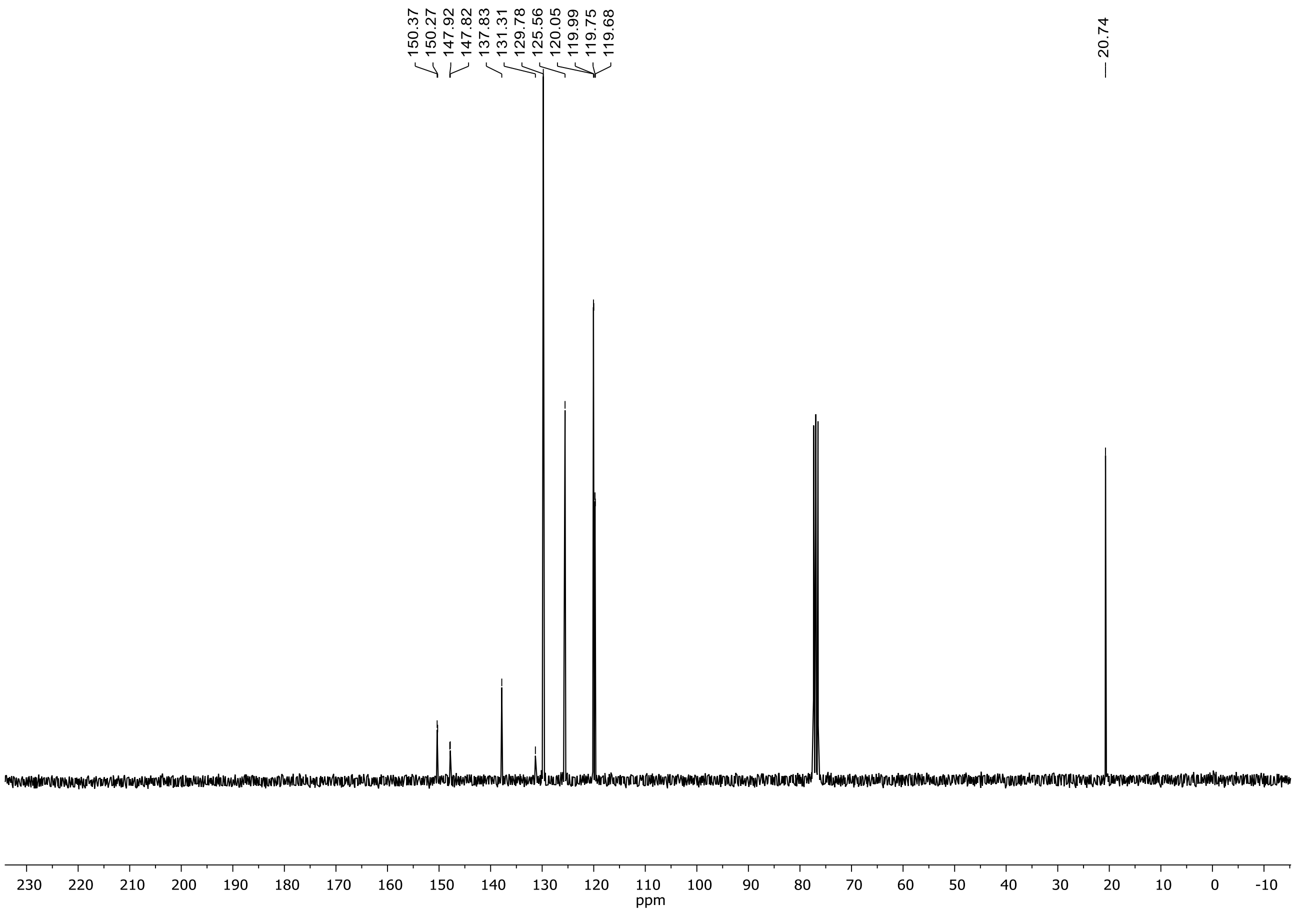

4-Chlorobiphenyl (**2a,** ^{1}H NMR, 300 MHz, $CDCl_3$)

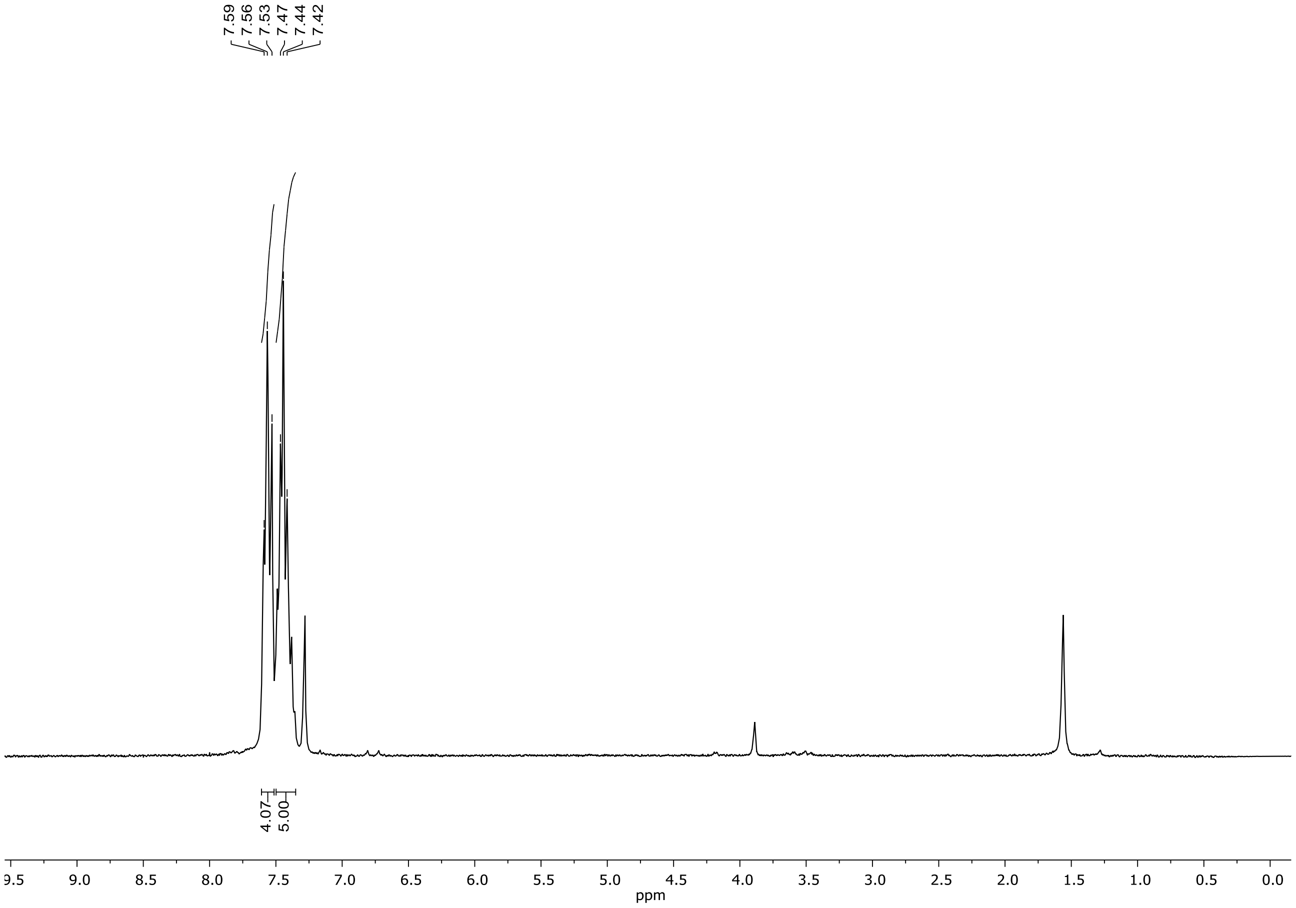

4-Chlorobiphenyl (**2a,** ^{13}C NMR, 75 MHz, $CDCl_3$)

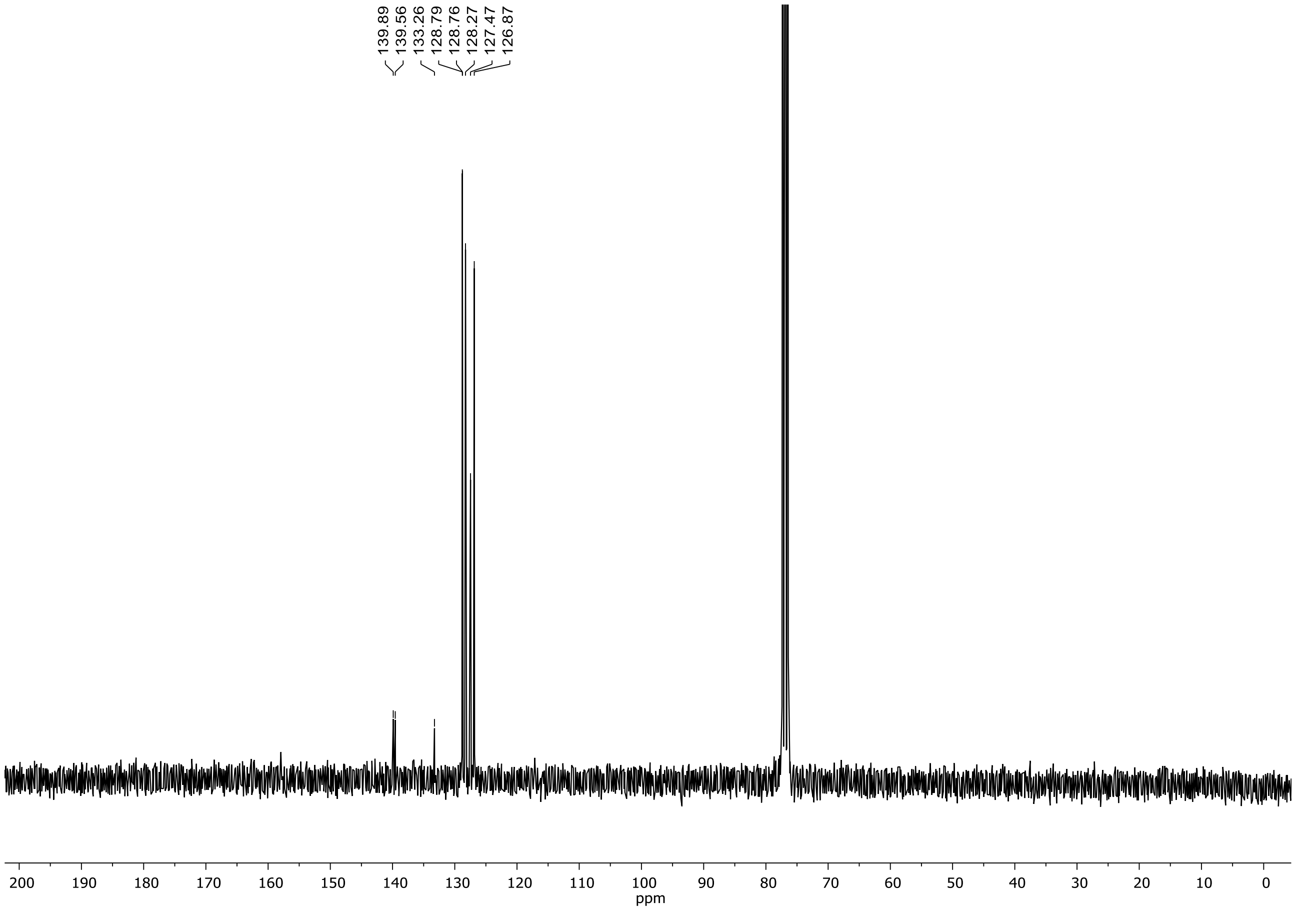

4-Methylbiphenyl (**2c,** ^{1}H NMR, 300 MHz, $CDCl_3$)

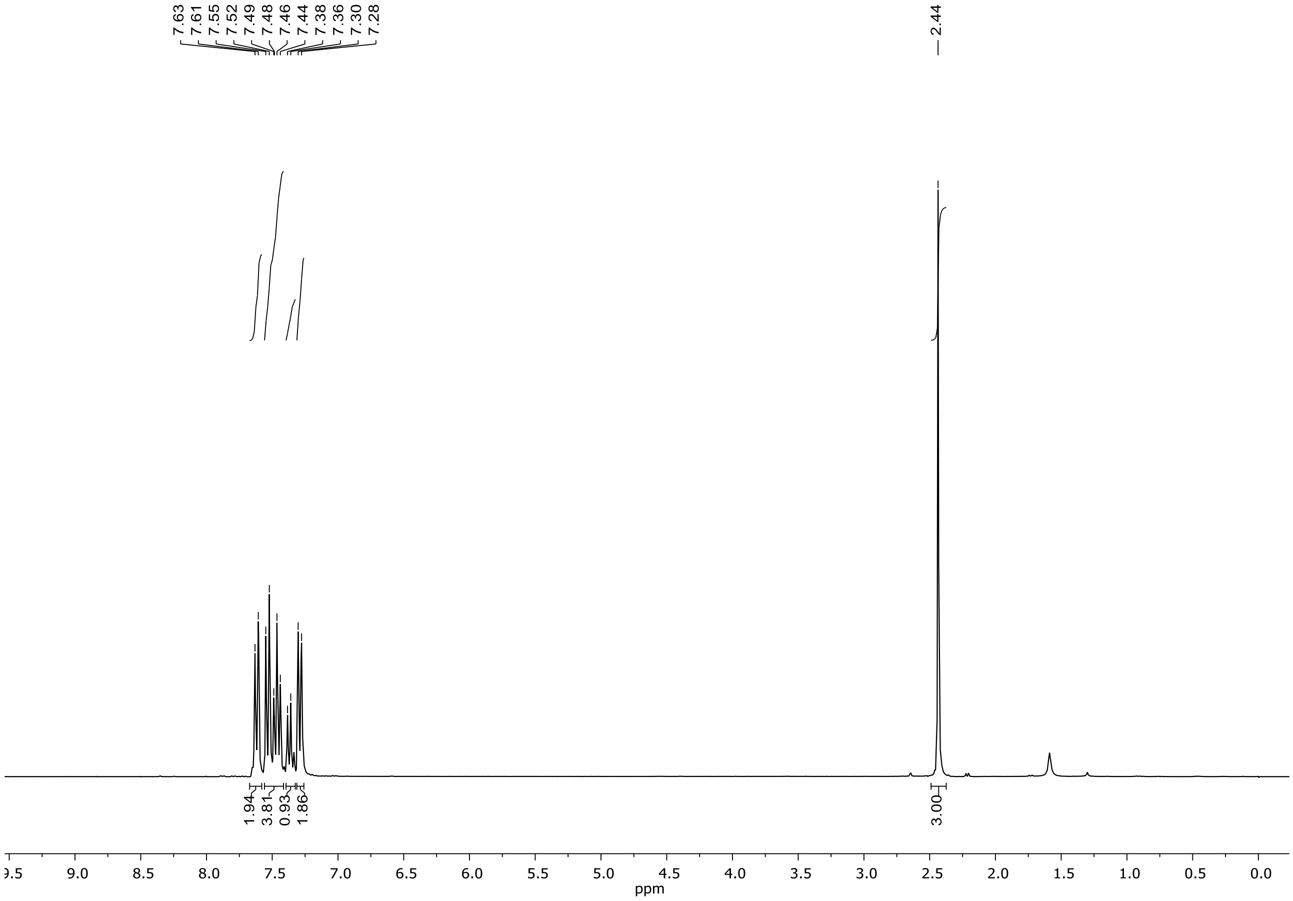

4-Methylbiphenyl (**2c,** ^{13}C NMR, 75 MHz, $CDCl_3$)

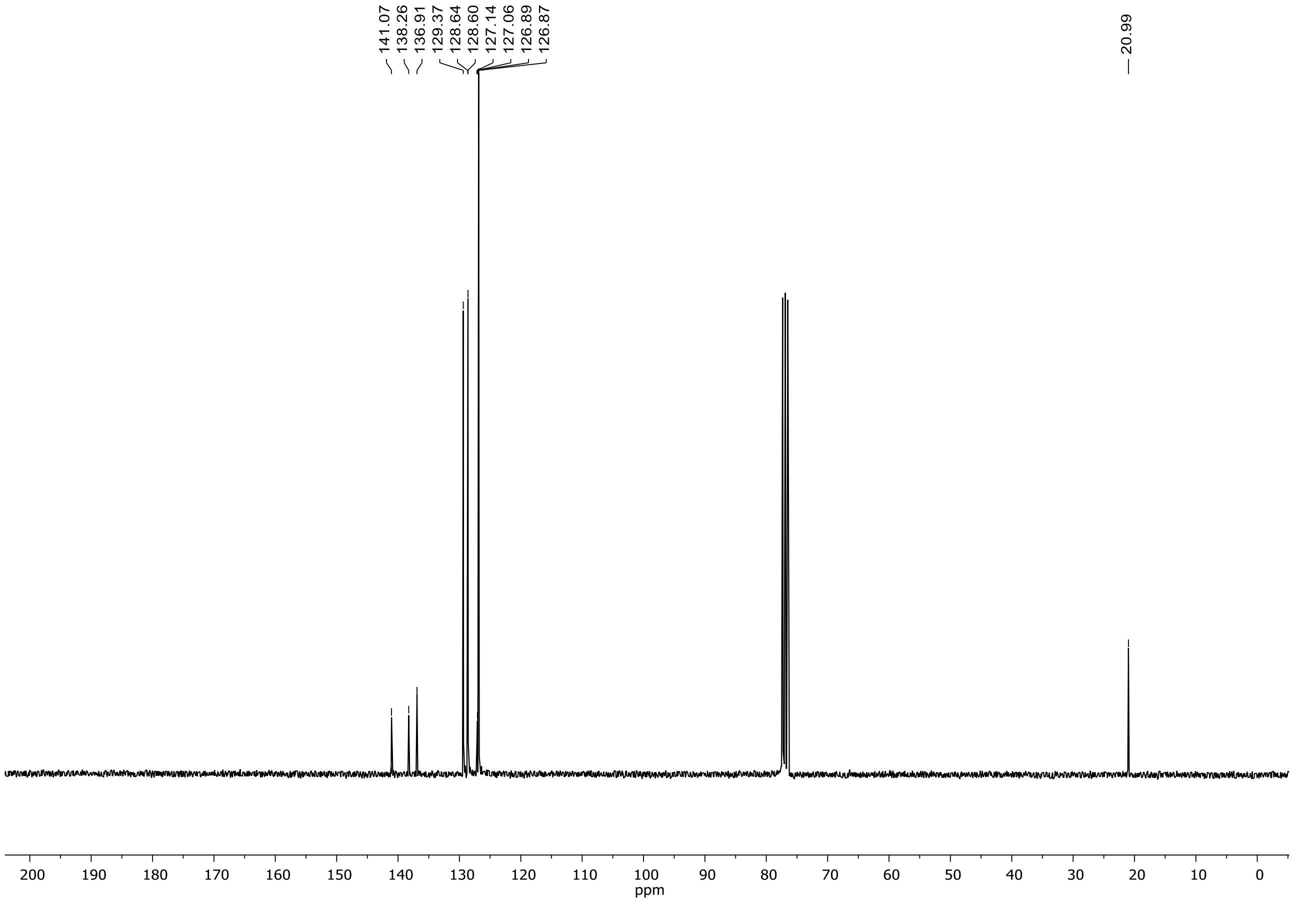

4-Isopropylbiphenyl (**2d,** ^{1}H NMR, 300 MHz, $CDCl_3$)

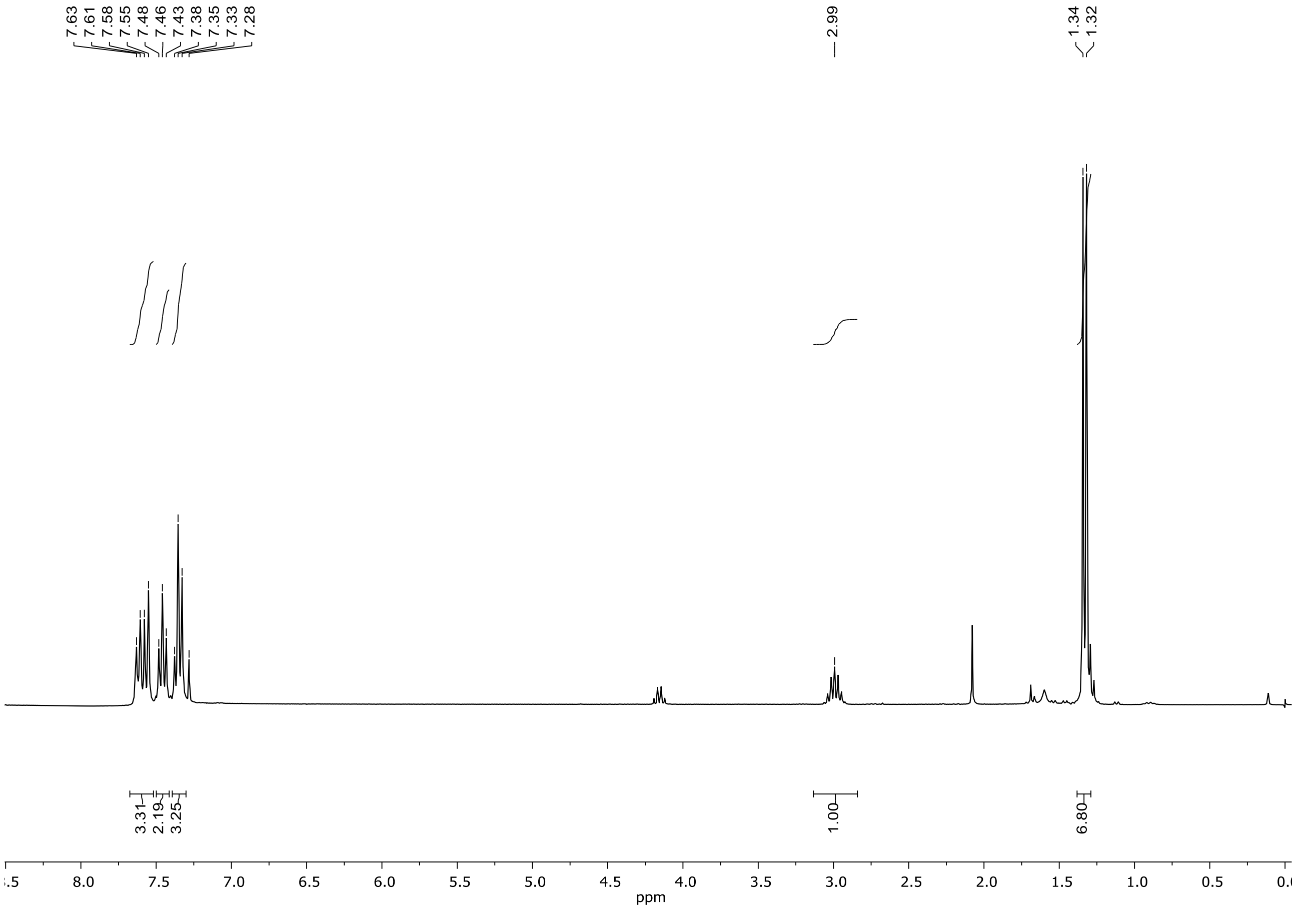

4-Isopropylbiphenyl (**2d,** ^{13}C NMR, 75 MHz, $CDCl_3$)

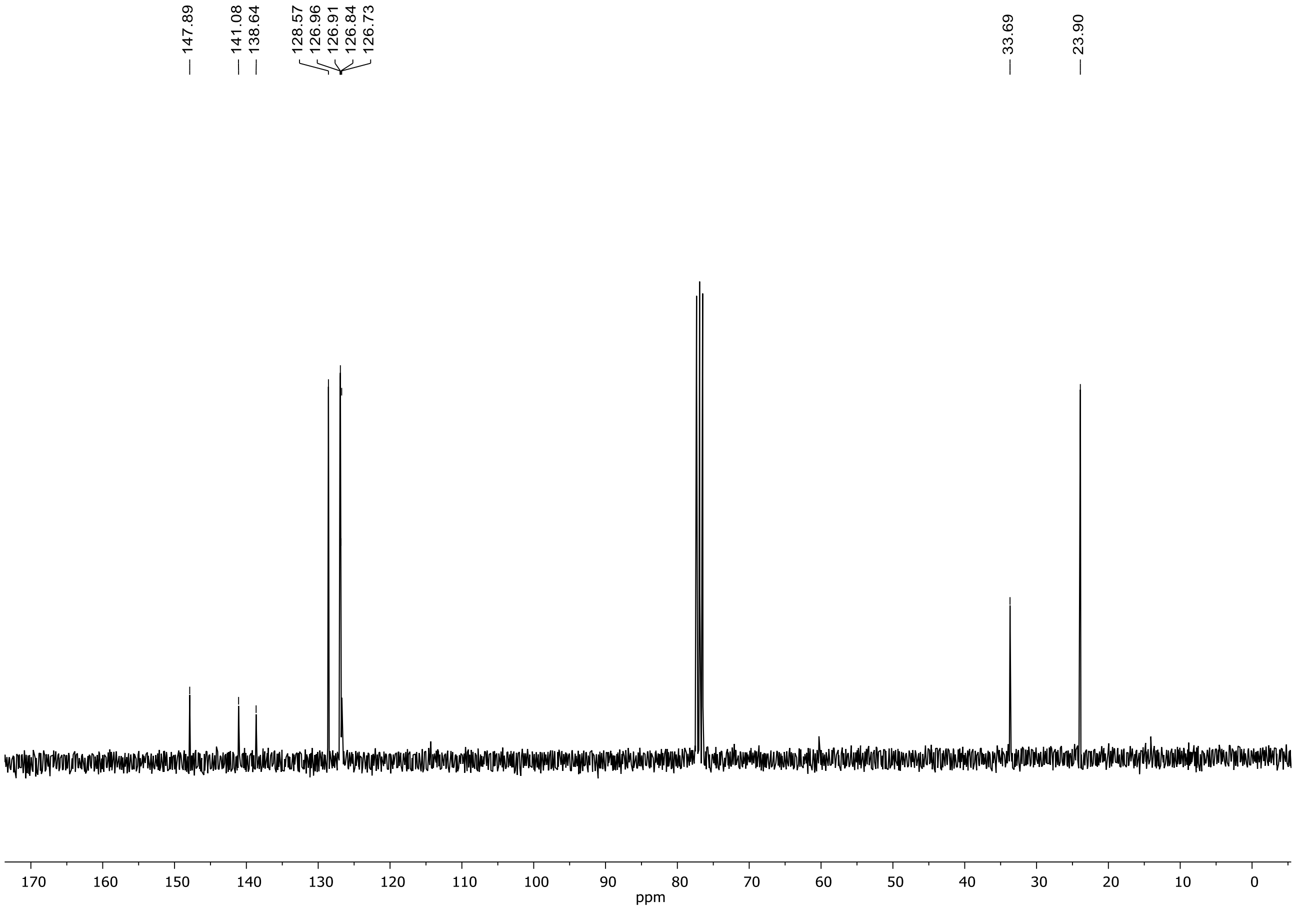

4-*tert*-Butylbiphenyl (2e, ^{1}H NMR, 300 MHz, $CDCl_3$).

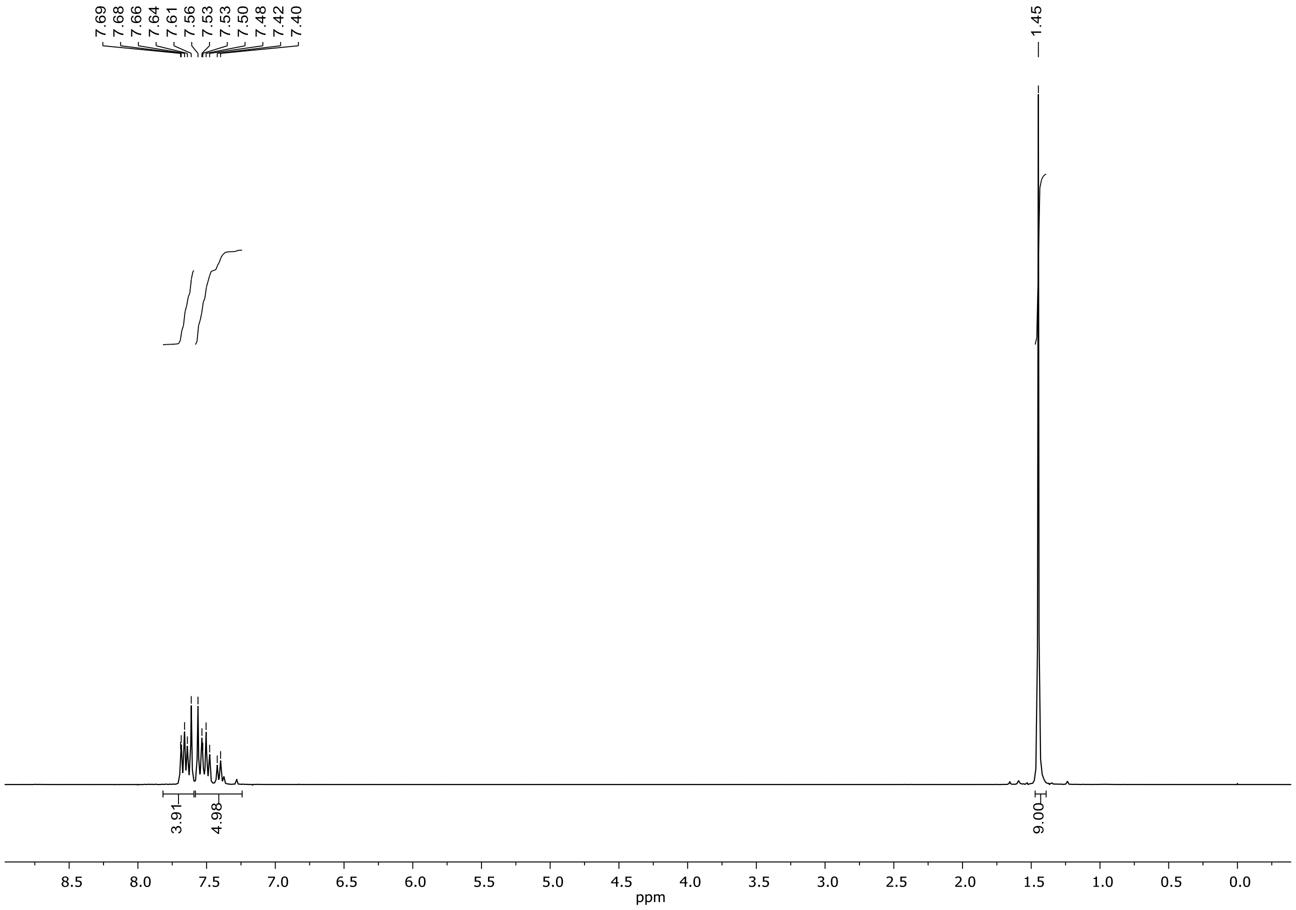

4-*tert*-Butylbiphenyl (**2e,** ^{13}C NMR, 75 MHz, $CDCl_3$).

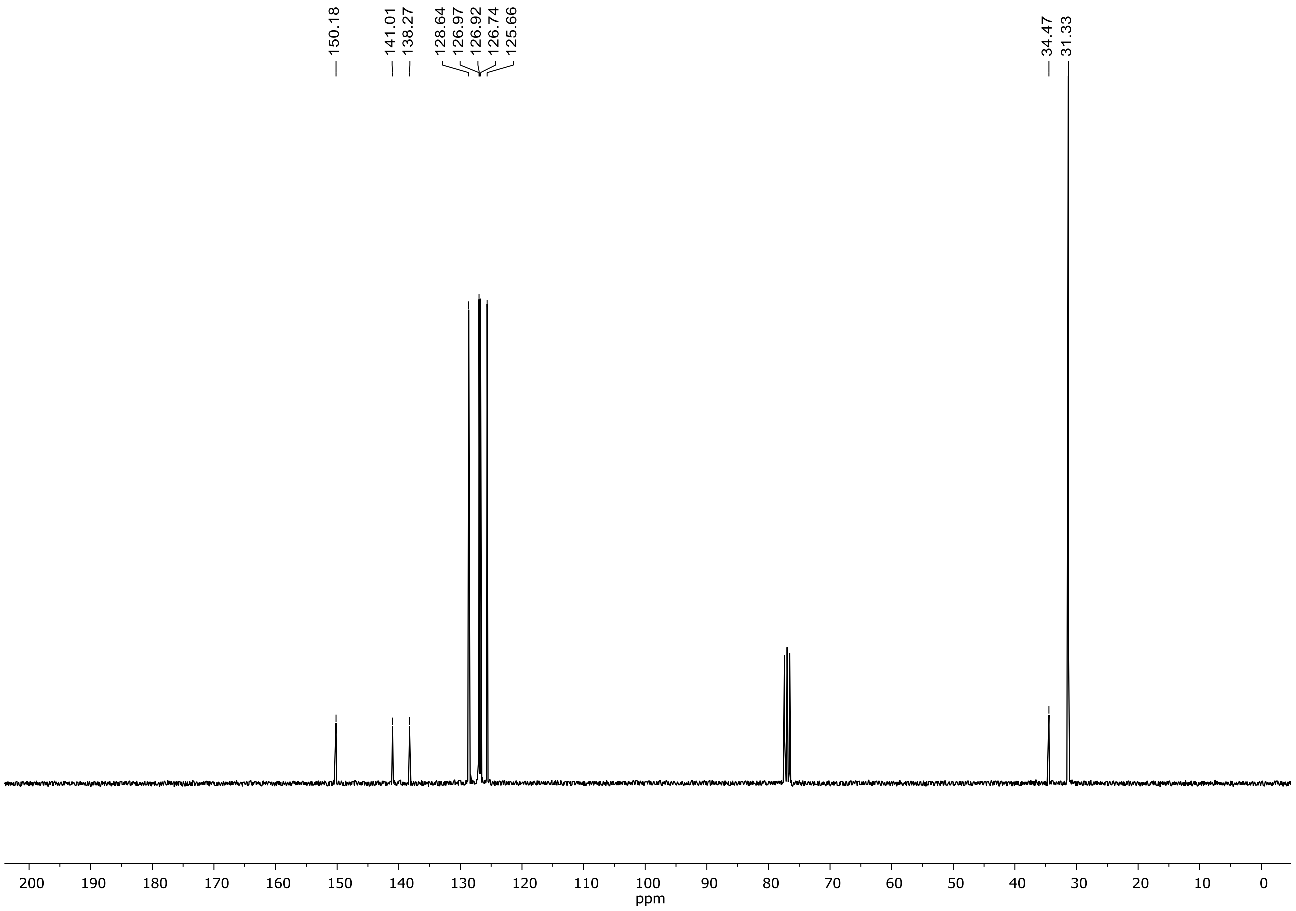

4-Methoxybiphenyl (**2f,** ^{1}H NMR, 300 MHz, $CDCl_3$).

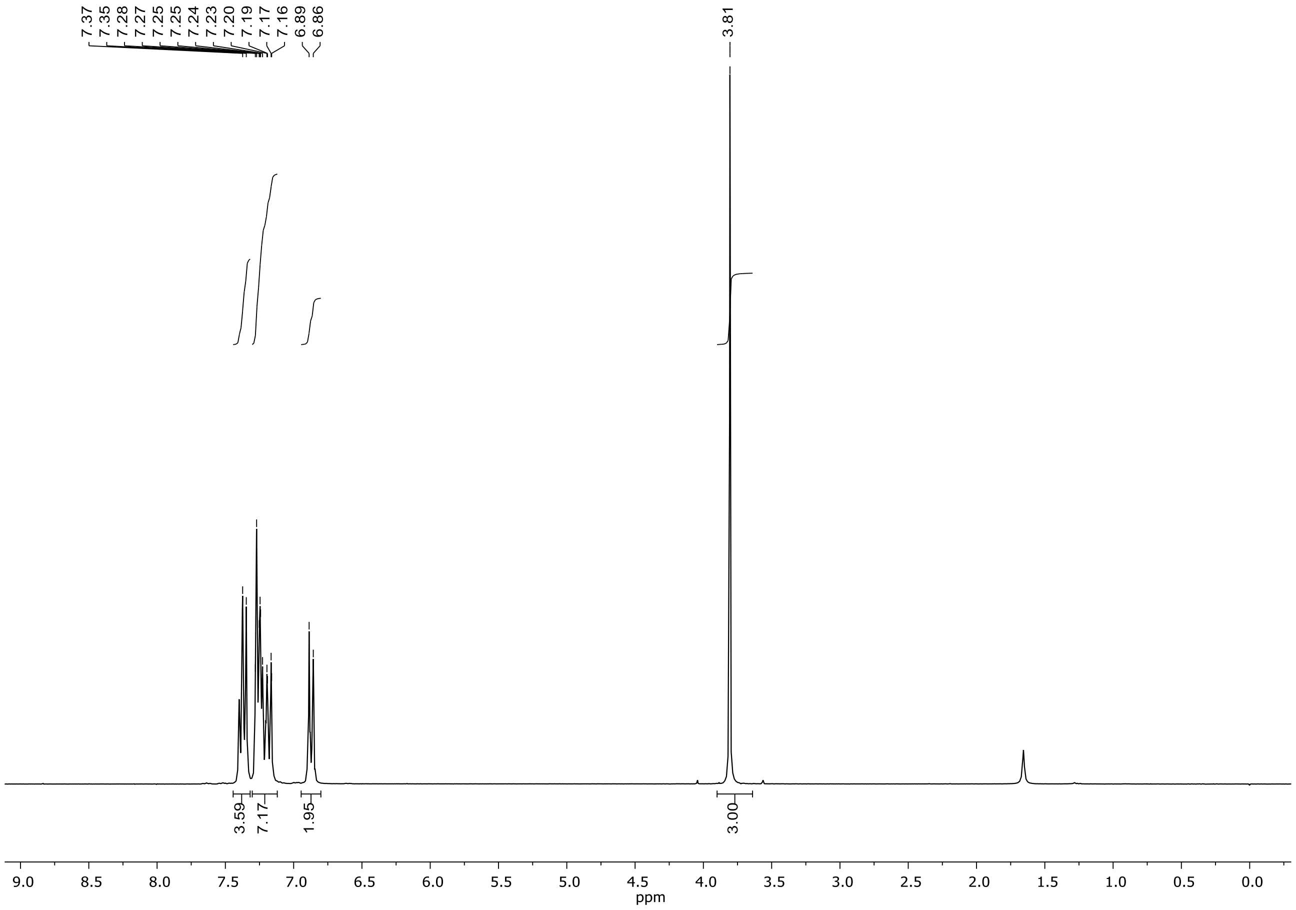

4-Methoxybiphenyl (**2f,** ^{13}C NMR, 75 MHz, $CDCl_3$).

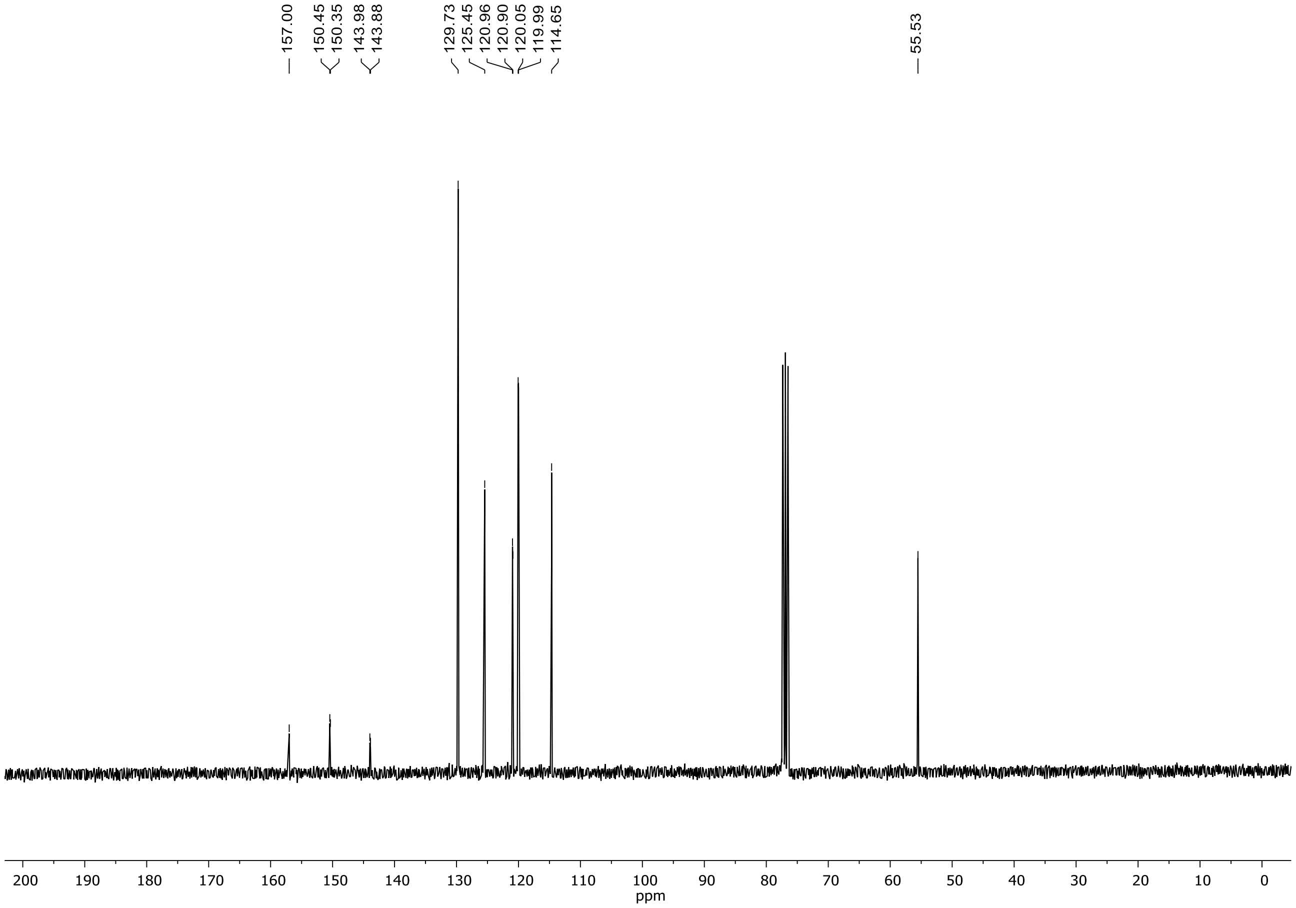

4-Phenoxybiphenyl (**2g,** ^{1}H NMR, 300 MHz, $CDCl_3$).

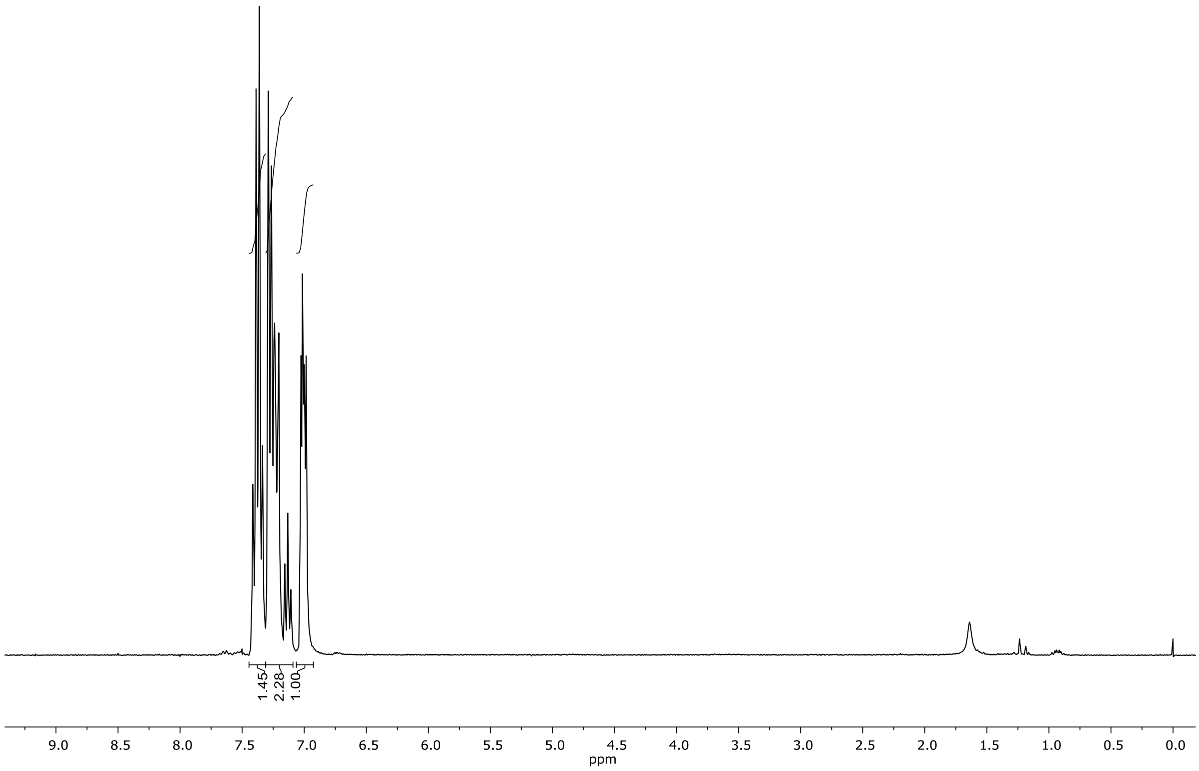

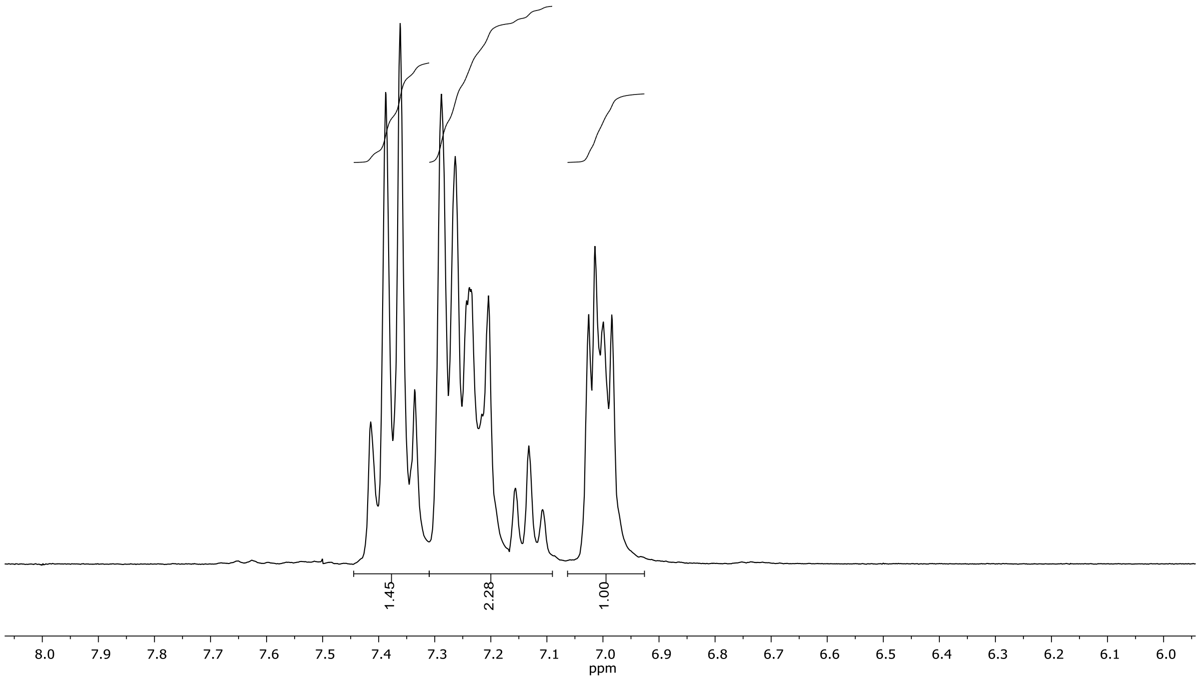

4-Phenoxybiphenyl (**2g,** ^{13}C NMR, 75 MHz, $CDCl_3$).

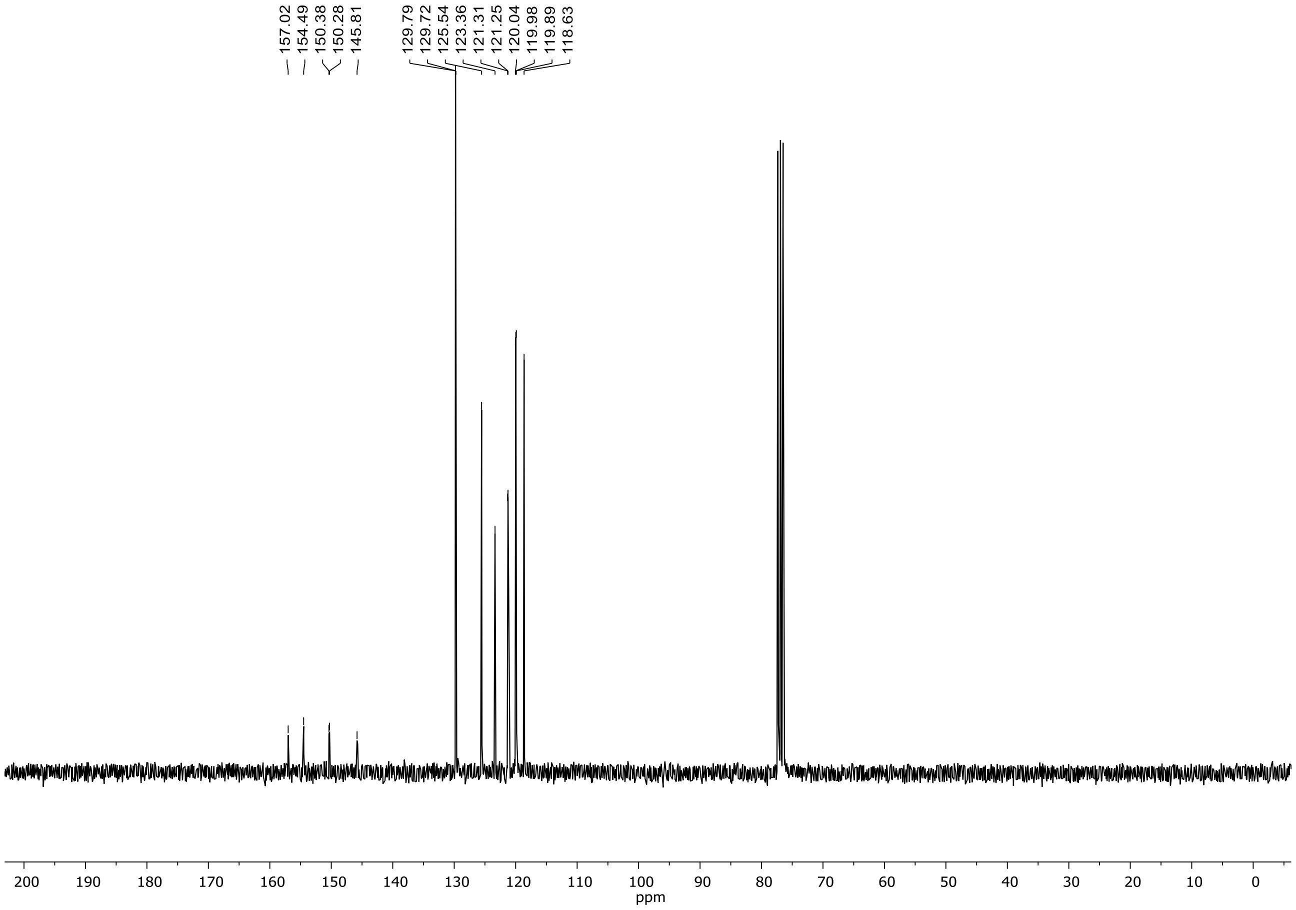

3-Methoxybiphenyl (**2i,** ^{1}H NMR, 300 MHz, $CDCl_3$).

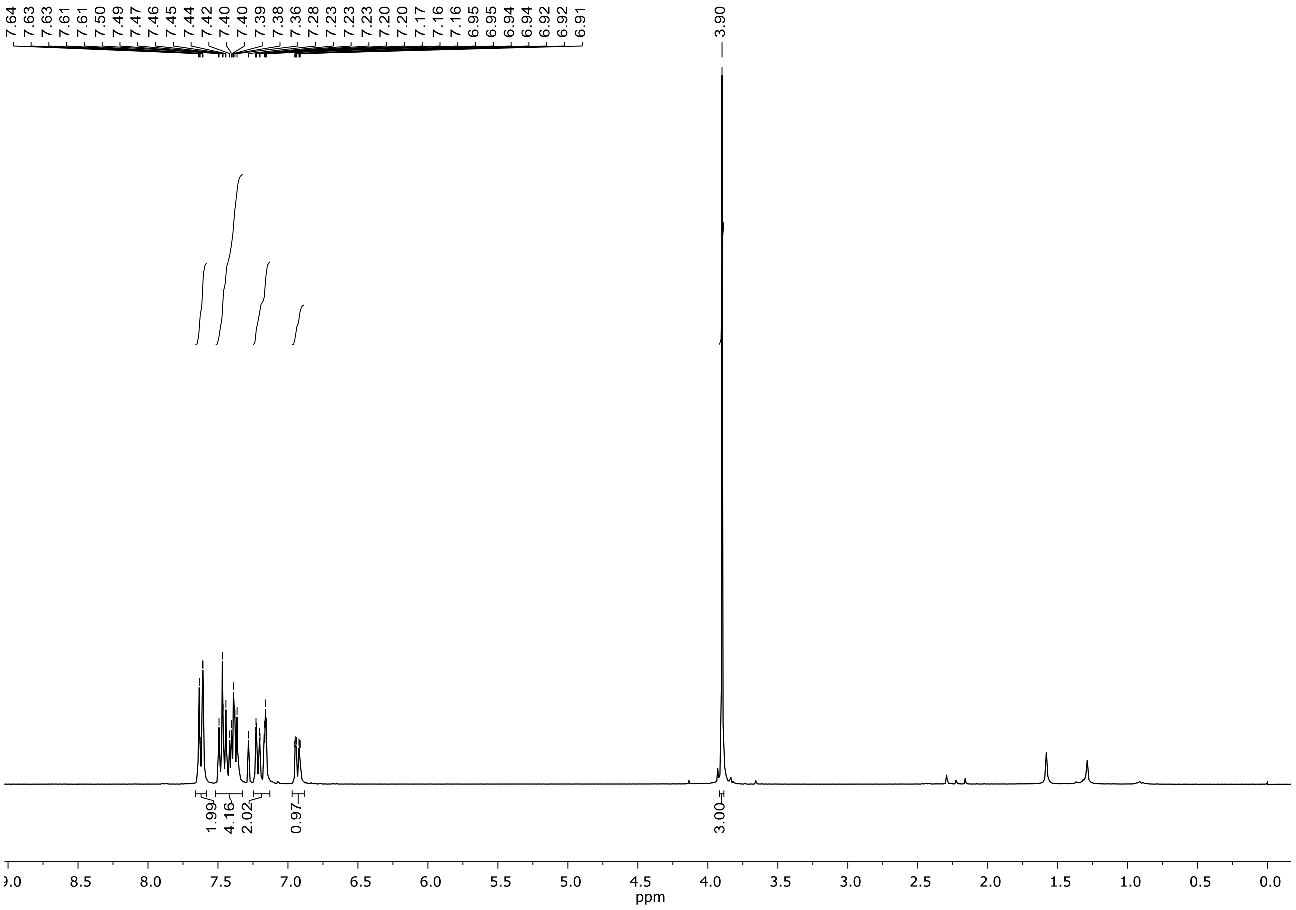

3-Methoxybiphenyl (**2i,** ^{13}C NMR, 75 MHz, $CDCl_3$).

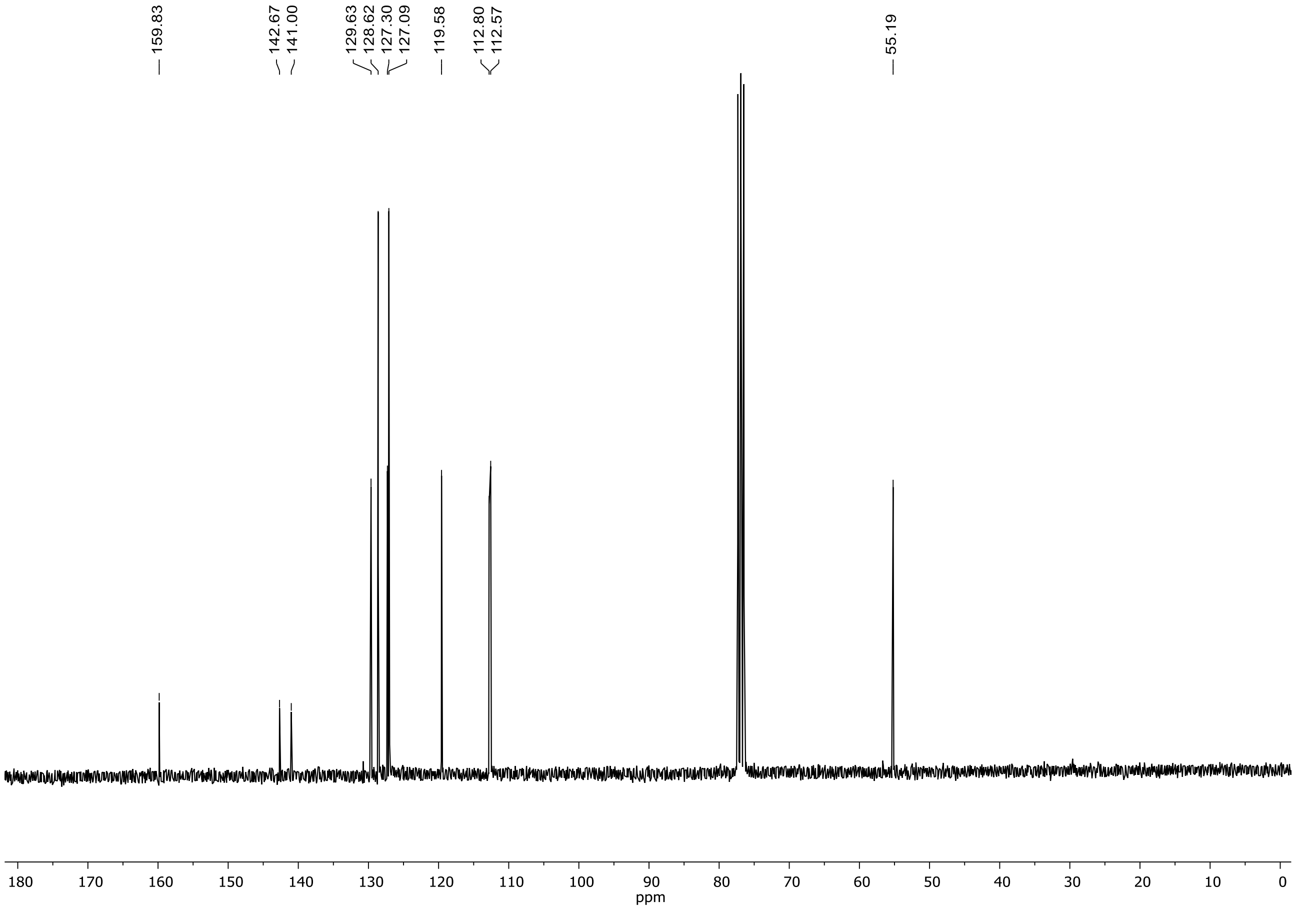

3,5-Dimethylbiphenyl (**2j,** ^{1}H NMR, 300 MHz, $CDCl_3$).

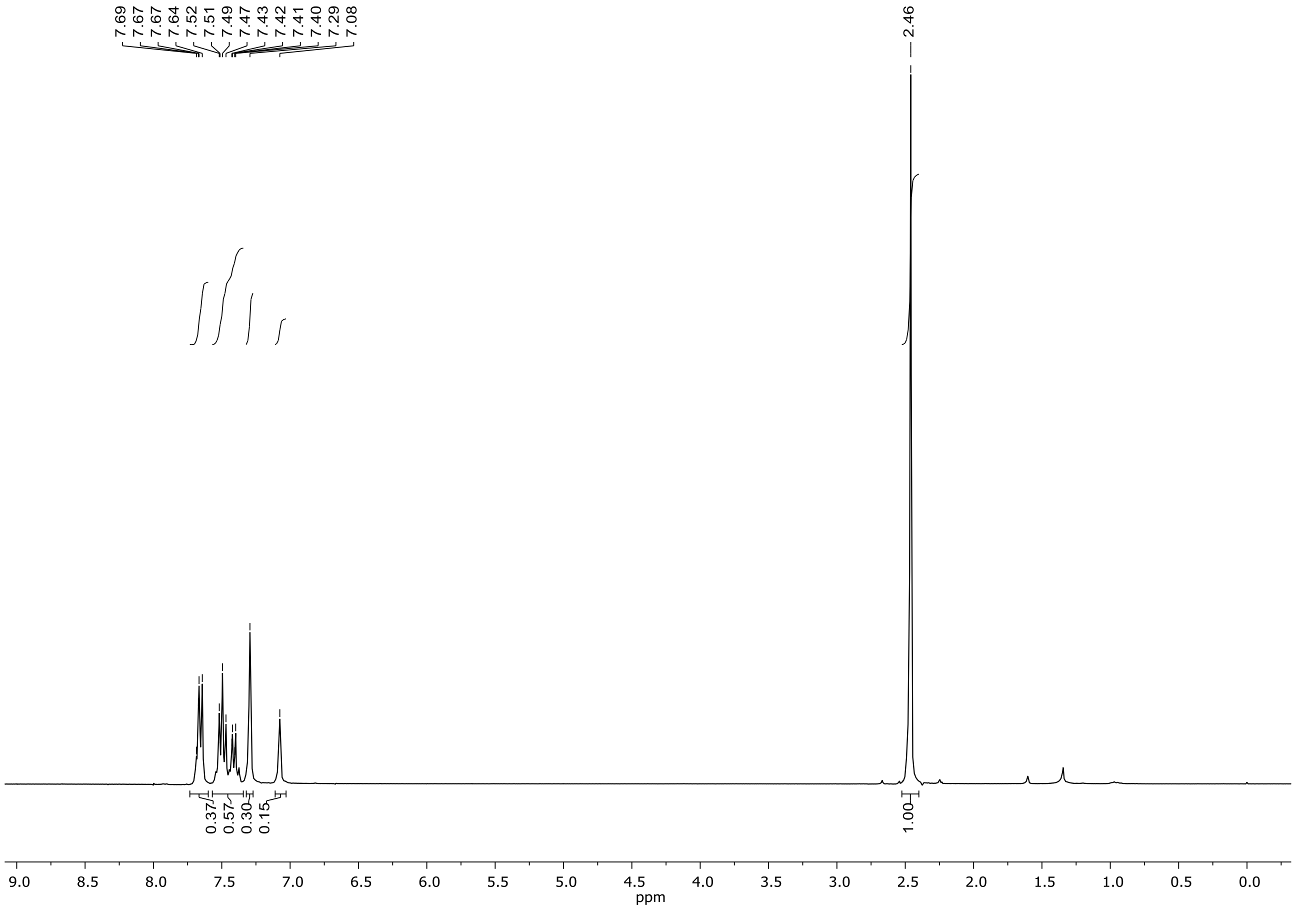

3,5-Dimethylbiphenyl (**2j,** ^{13}C NMR, 75 MHz, $CDCl_3$).

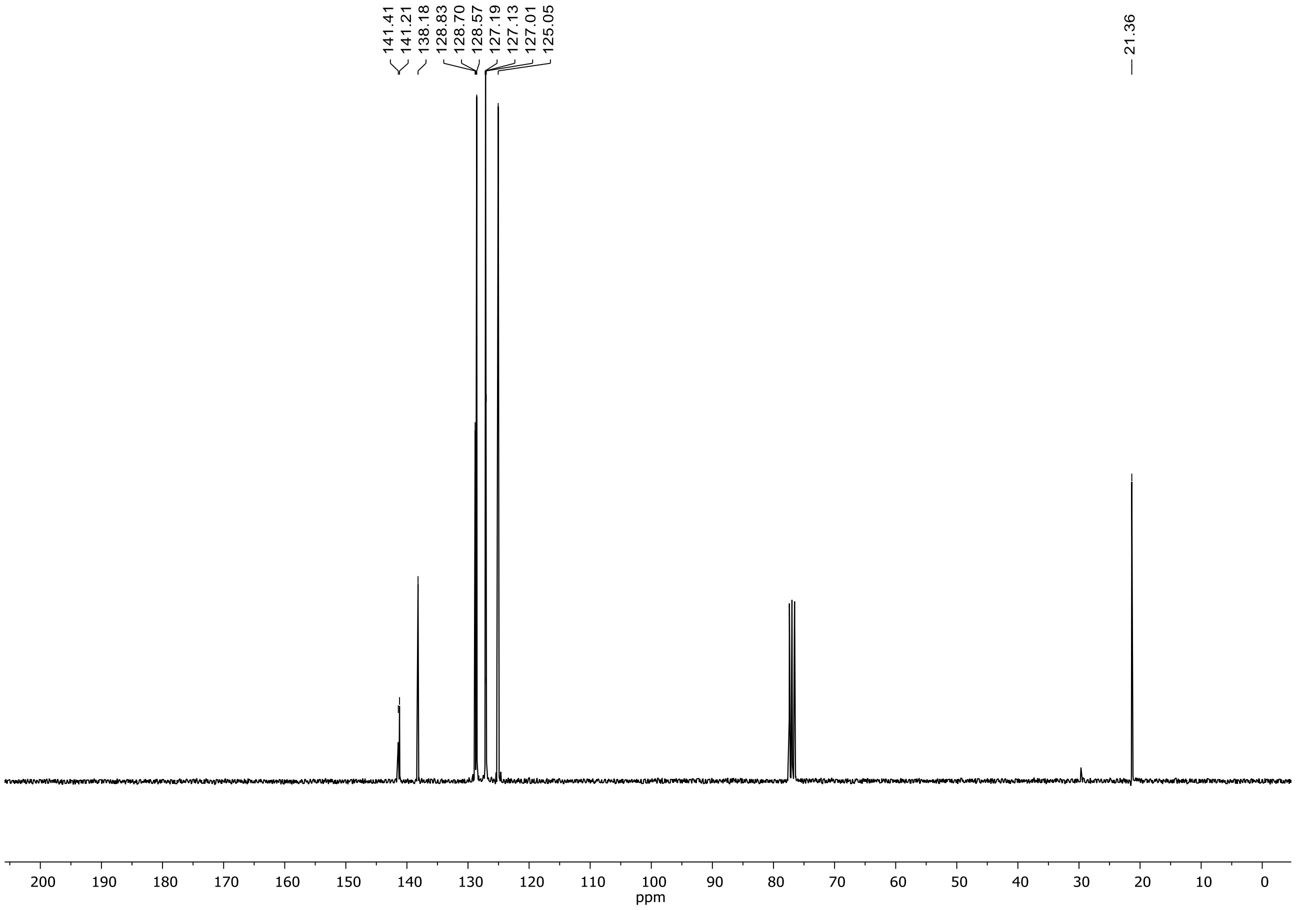

2,3,5-Trimethylbiphenyl (**2k,** ^{1}H NMR, 300 MHz, $CDCl_3$).

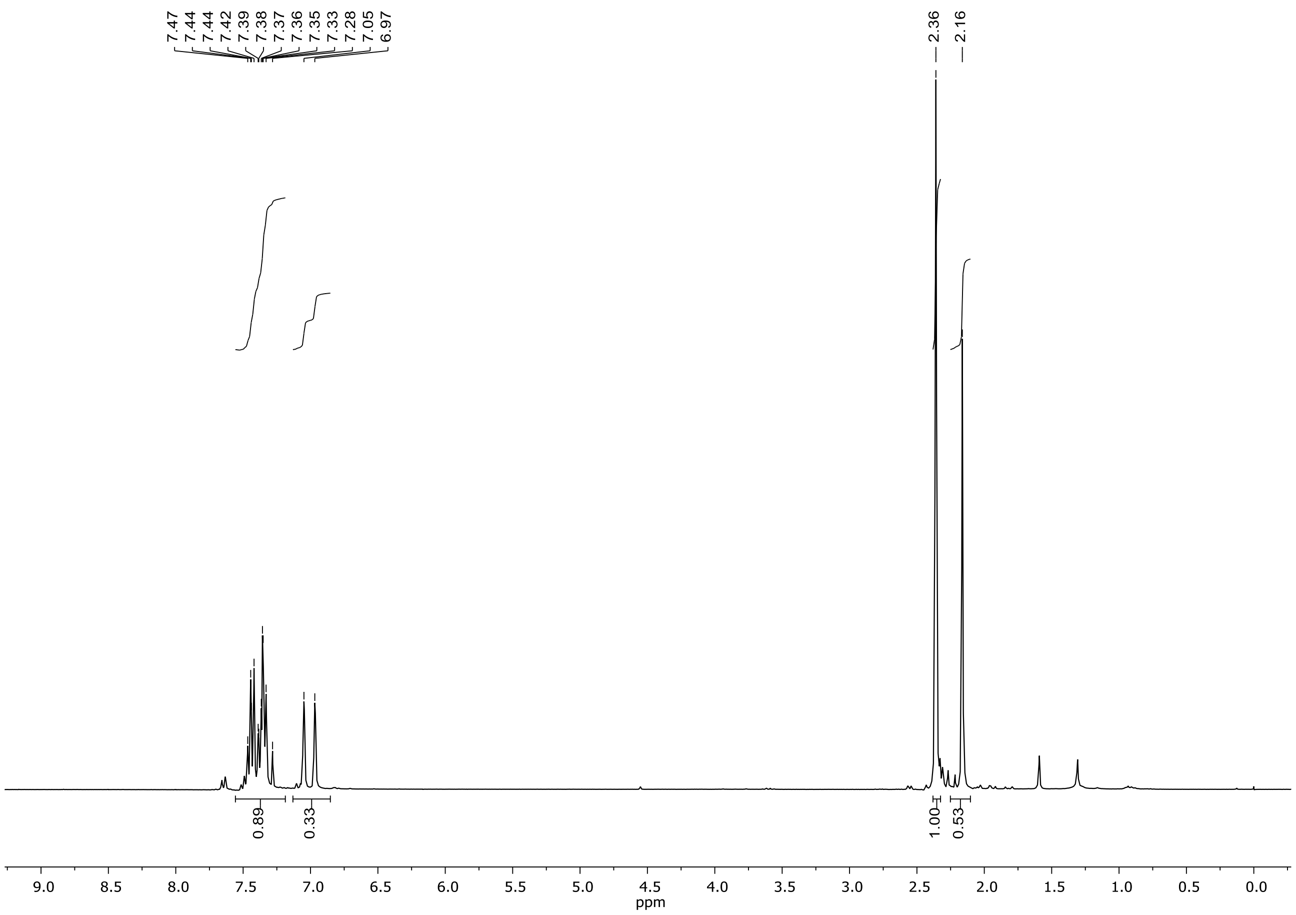

2,3,5-Trimethylbiphenyl (**2k,** ^{13}C NMR, 75 MHz, $CDCl_3$).

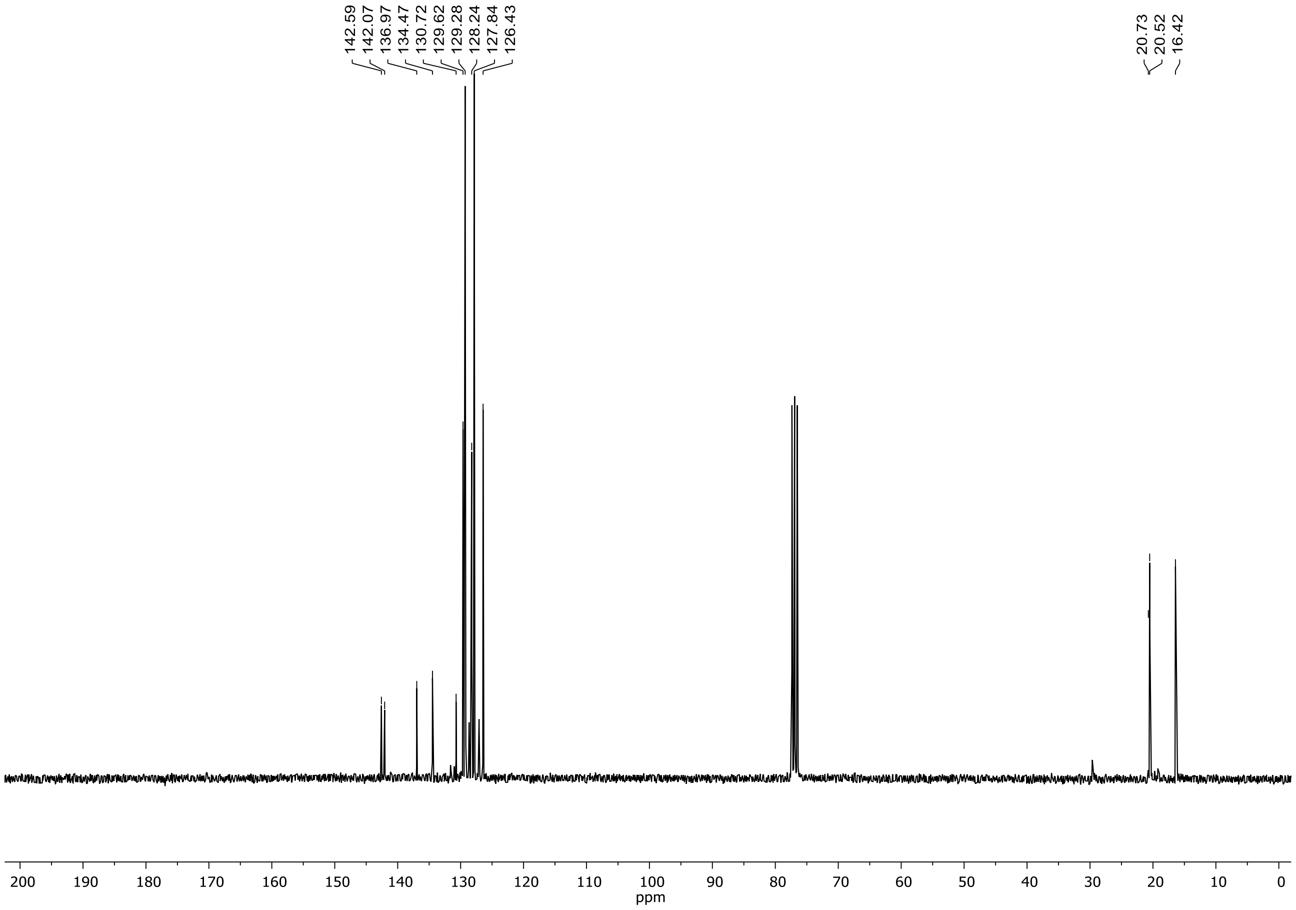

3,5-Dimethyl-4-chlorobiphenyl (**2l,** ^{1}H NMR, 300 MHz, $CDCl_3$).

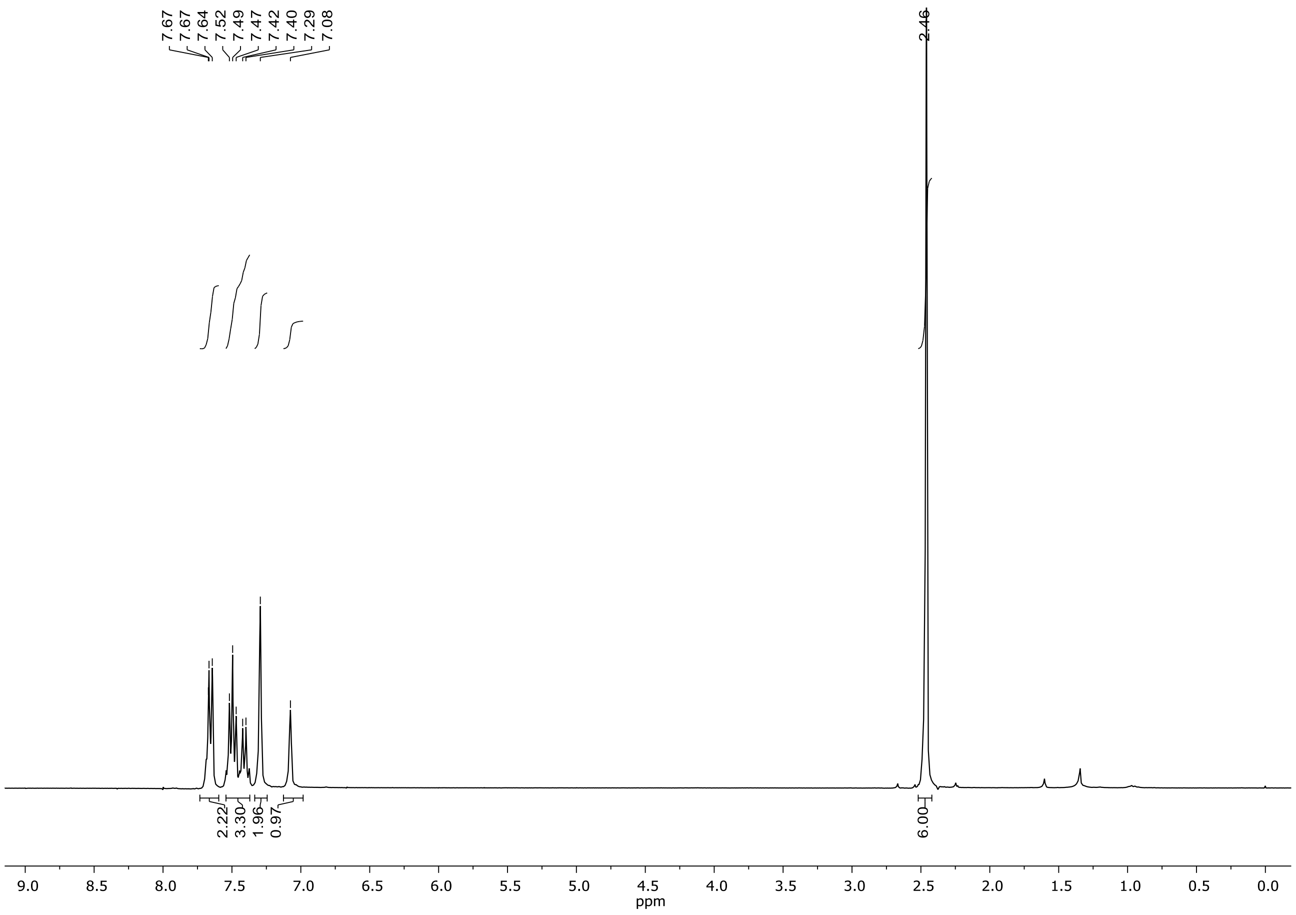

3,5-Dimethyl-4-chlorobiphenyl (**2l,** ^{13}C NMR, 75 MHz, $CDCl_3$).

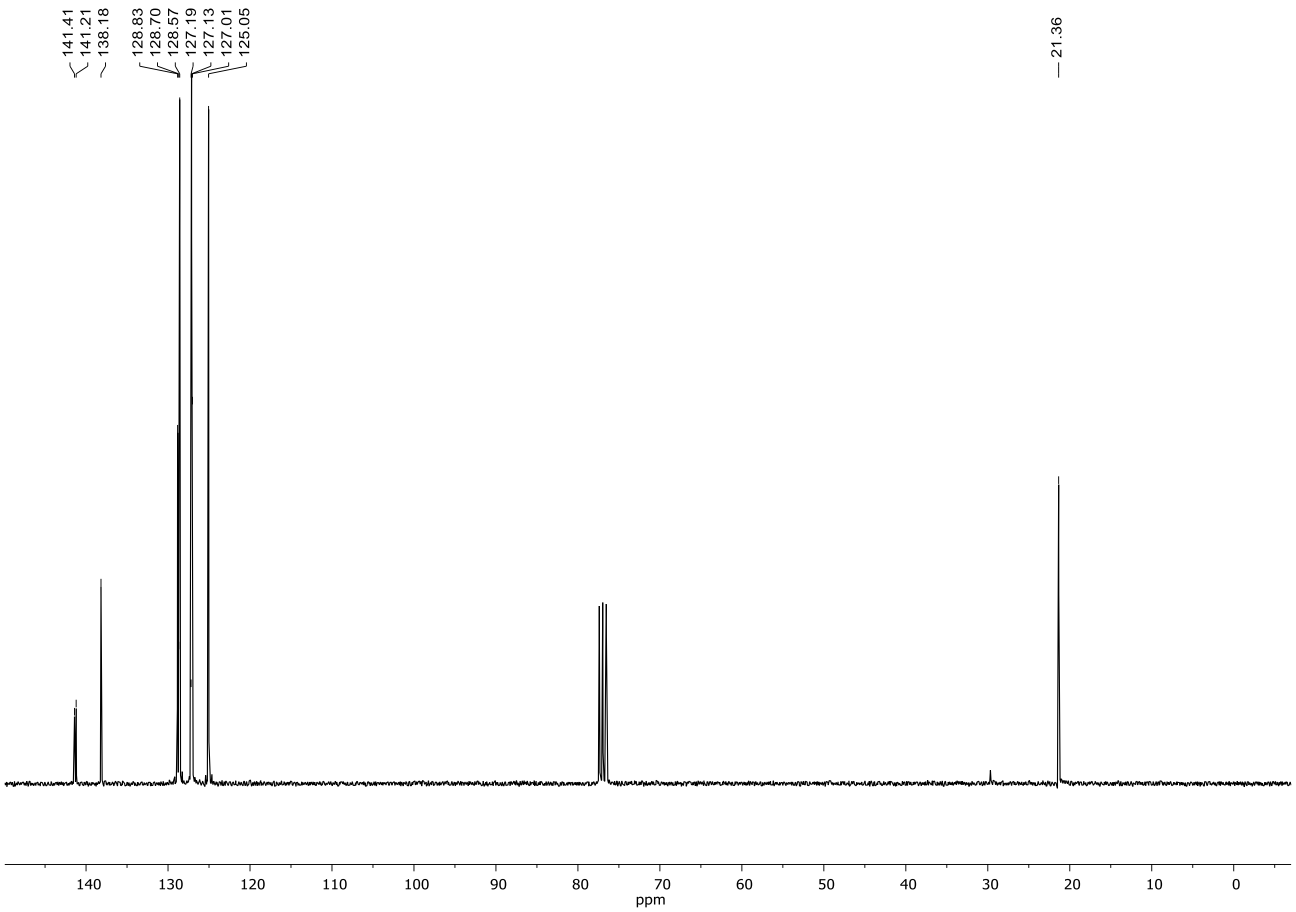

Ethyl bis(4-methoxyphenyl) phosphate (**3a,** ^{1}H NMR, 300 MHz, $CDCl_3$).

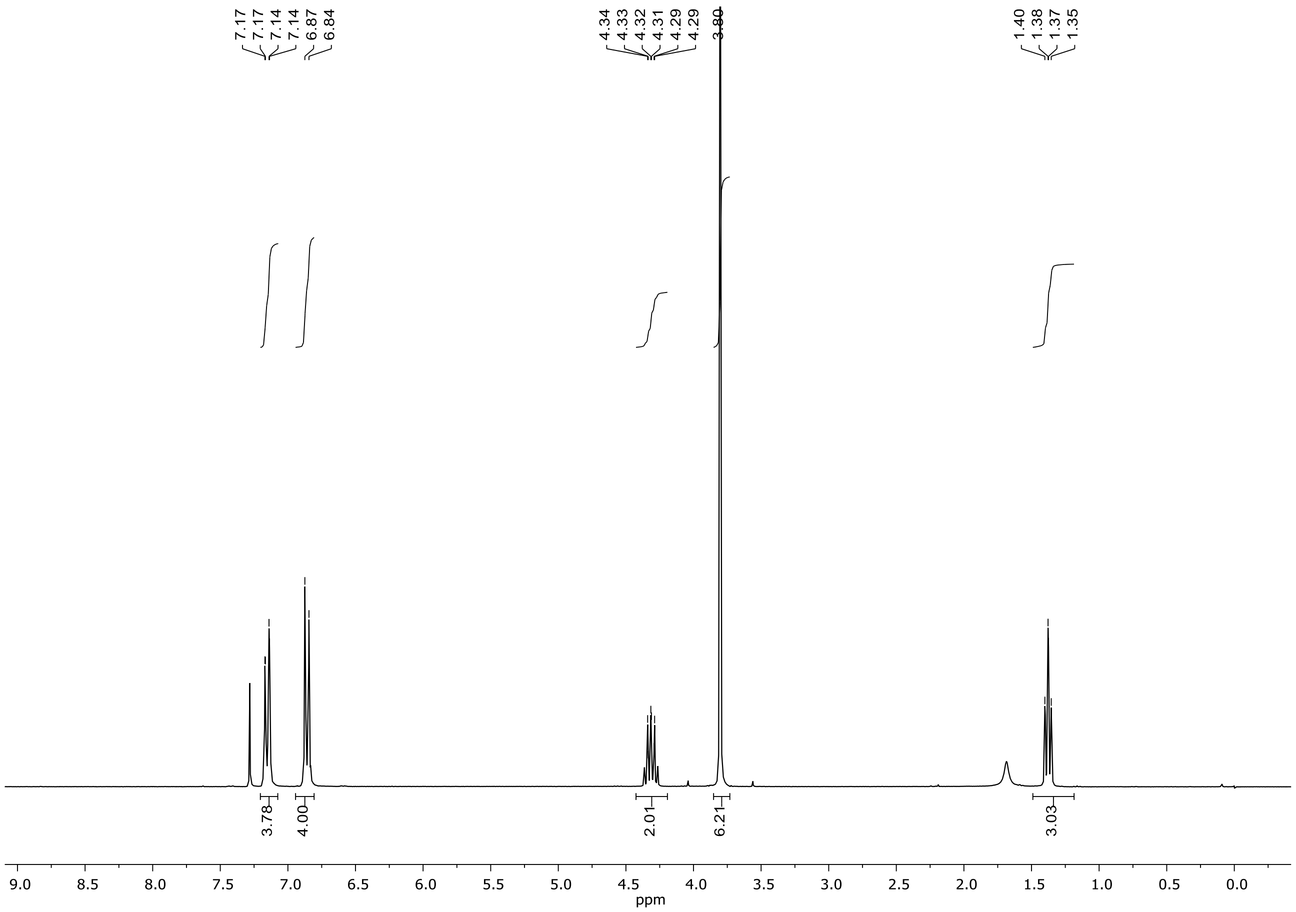

Ethyl bis(4-methoxyphenyl) phosphate (**3a,** [1]C NMR, 75 MHz, $CDCl_3$).

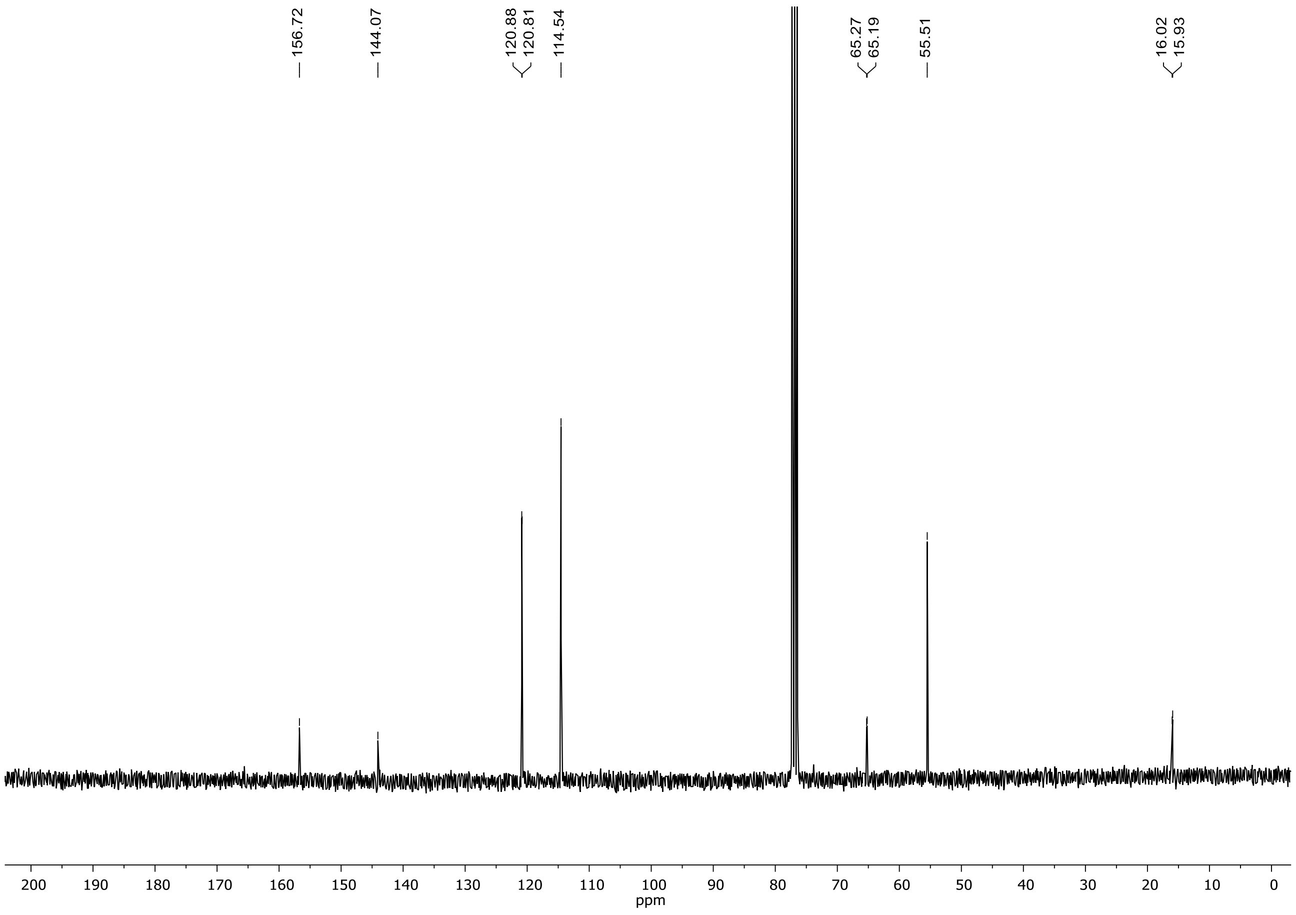

Ethyl bis(4-phenoxyphenyl) phosphate (**3b,** ^{1}H NMR, 300 MHz, $CDCl_3$).

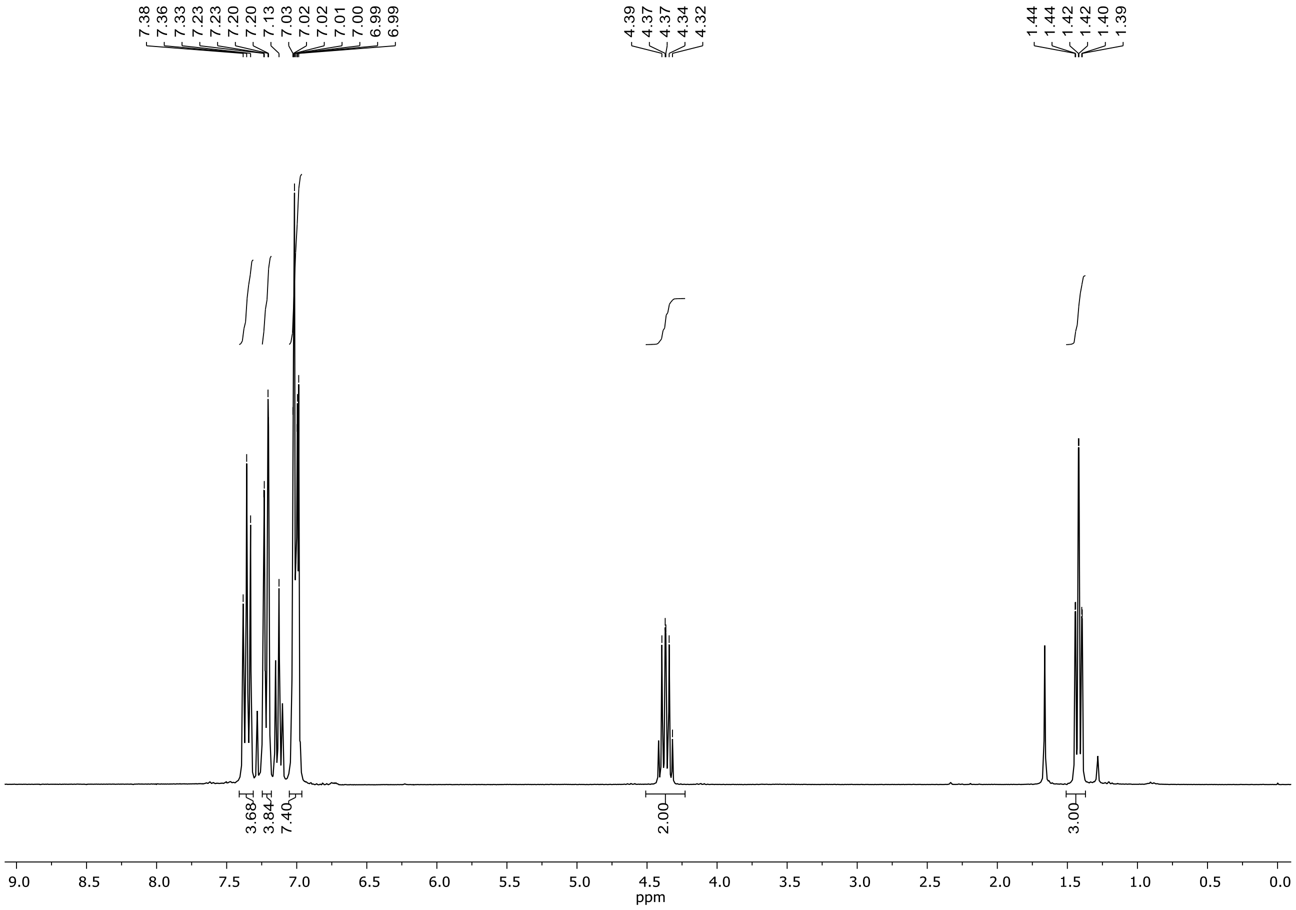

Ethyl bis(4-phenoxyphenyl) phosphate (**3b,** ^{13}C NMR, 75 MHz, $CDCl_3$).

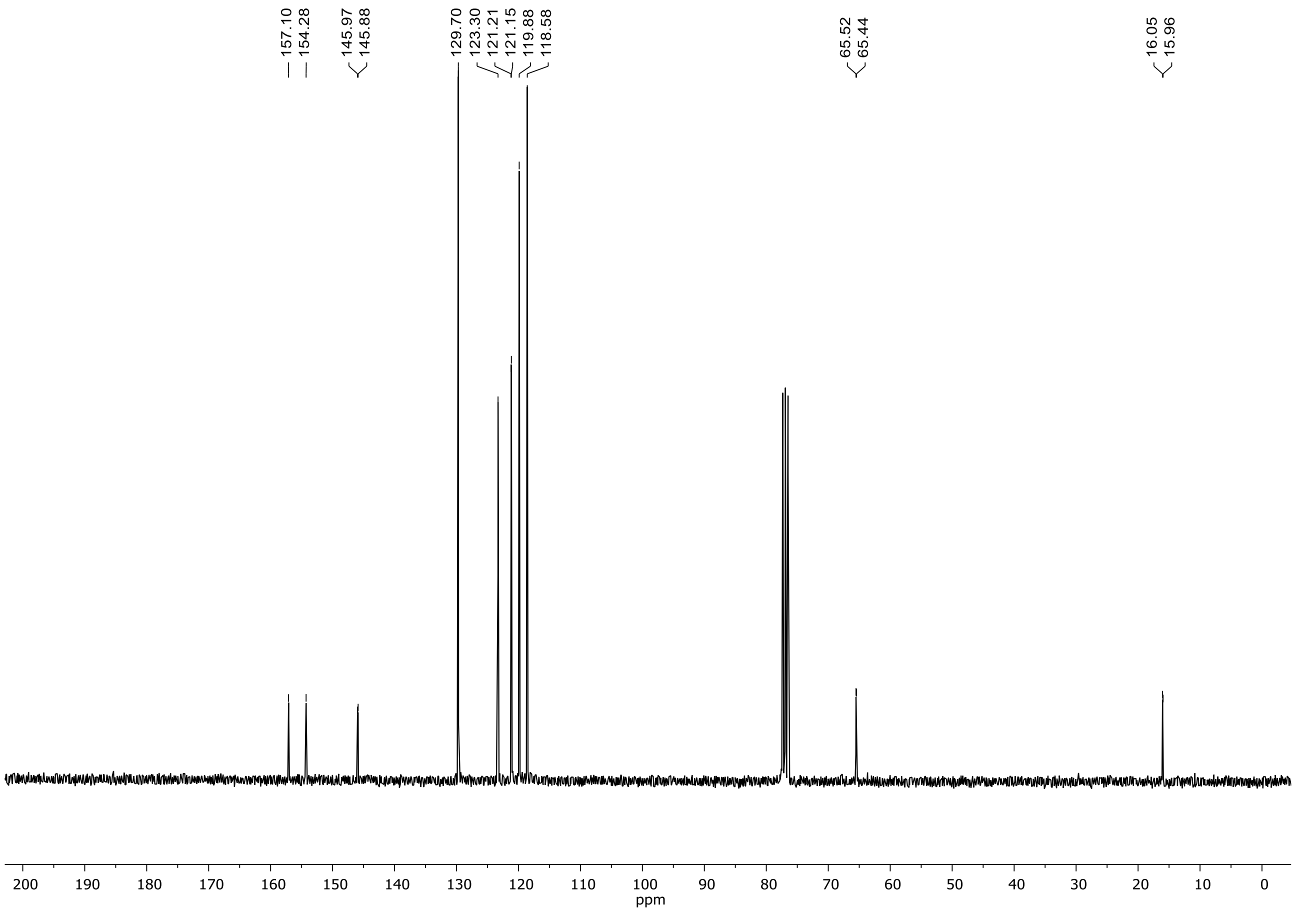

Ethyl bis(4-*tert*-butylphenyl) phosphate (**3c,** ^{1}H NMR, 300 MHz, $CDCl_3$).

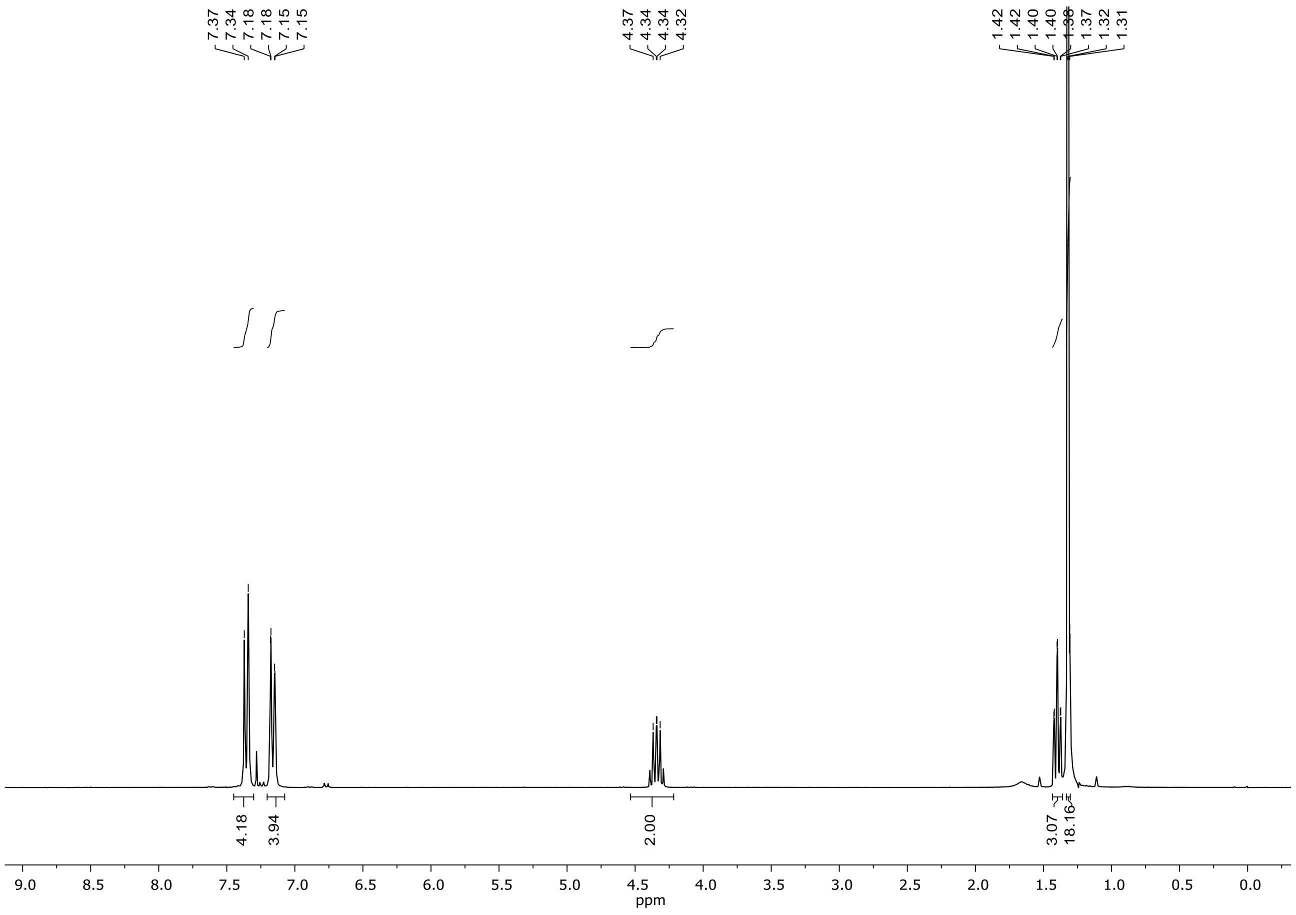

Ethyl bis(4-*tert*-butylphenyl) phosphate (**3c,** ^{13}C NMR, 75 MHz, $CDCl_3$).

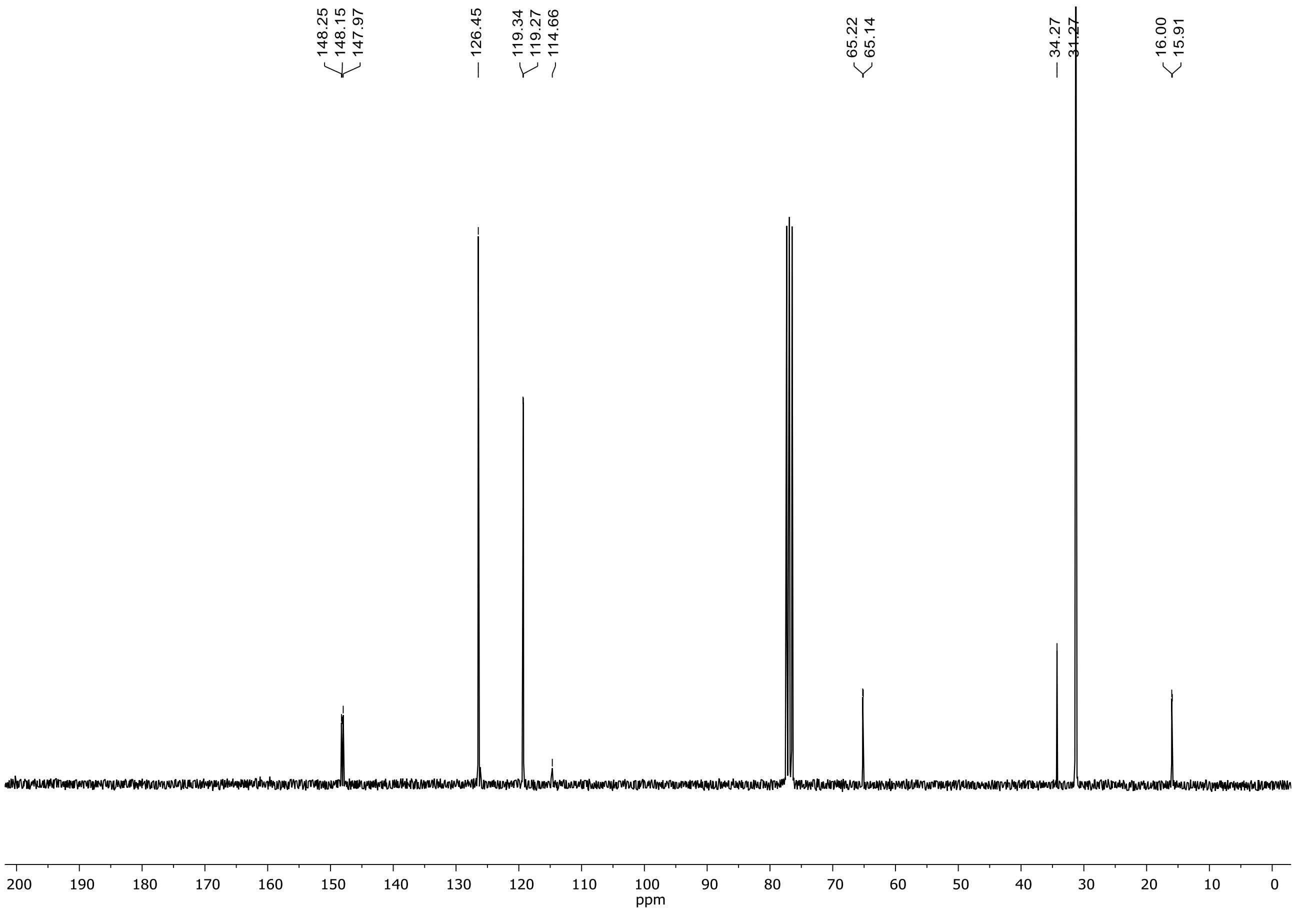

Ethyl bis(3-methoxyphenyl) phosphate (**3d,** ^{1}H NMR, 300 MHz, $CDCl_3$).

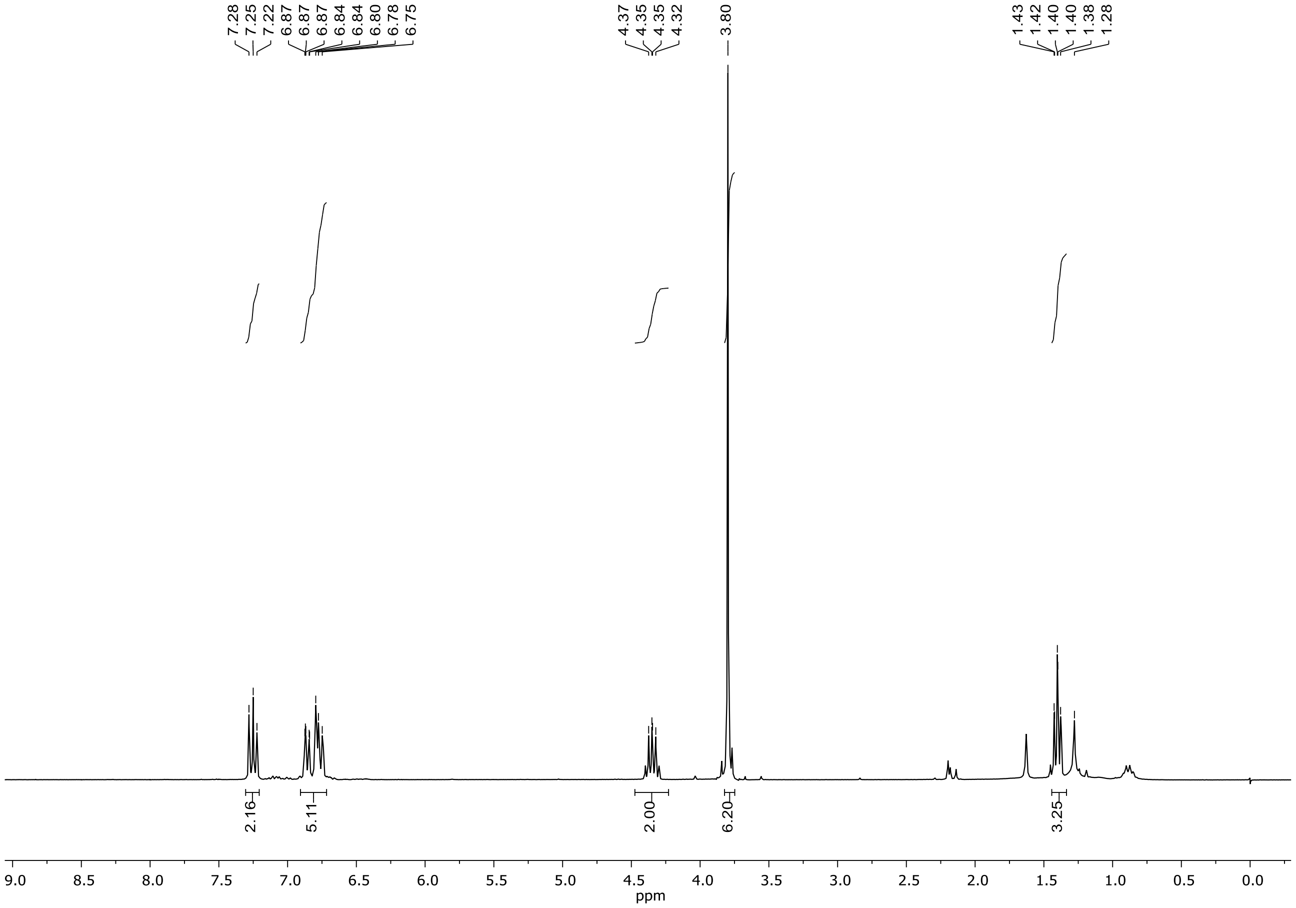

Ethyl bis(3-methoxyphenyl) phosphate (**3d,** ^{13}C NMR, 75 MHz, $CDCl_3$).

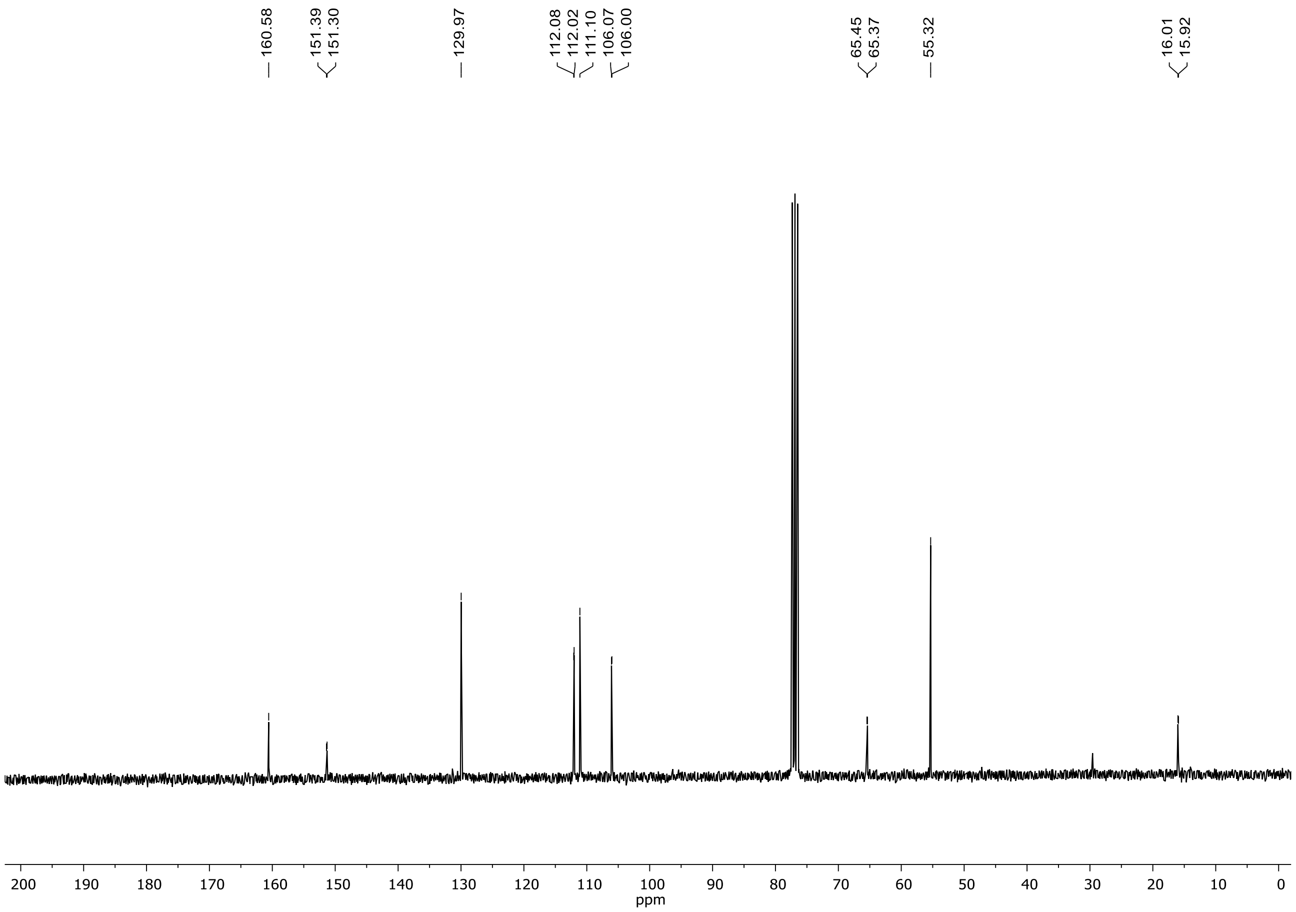

Ethyl bis-(2,4,6-trimethylphenyl) phosphate (**3e,** ^{1}H NMR, 300 MHz, $CDCl_3$).

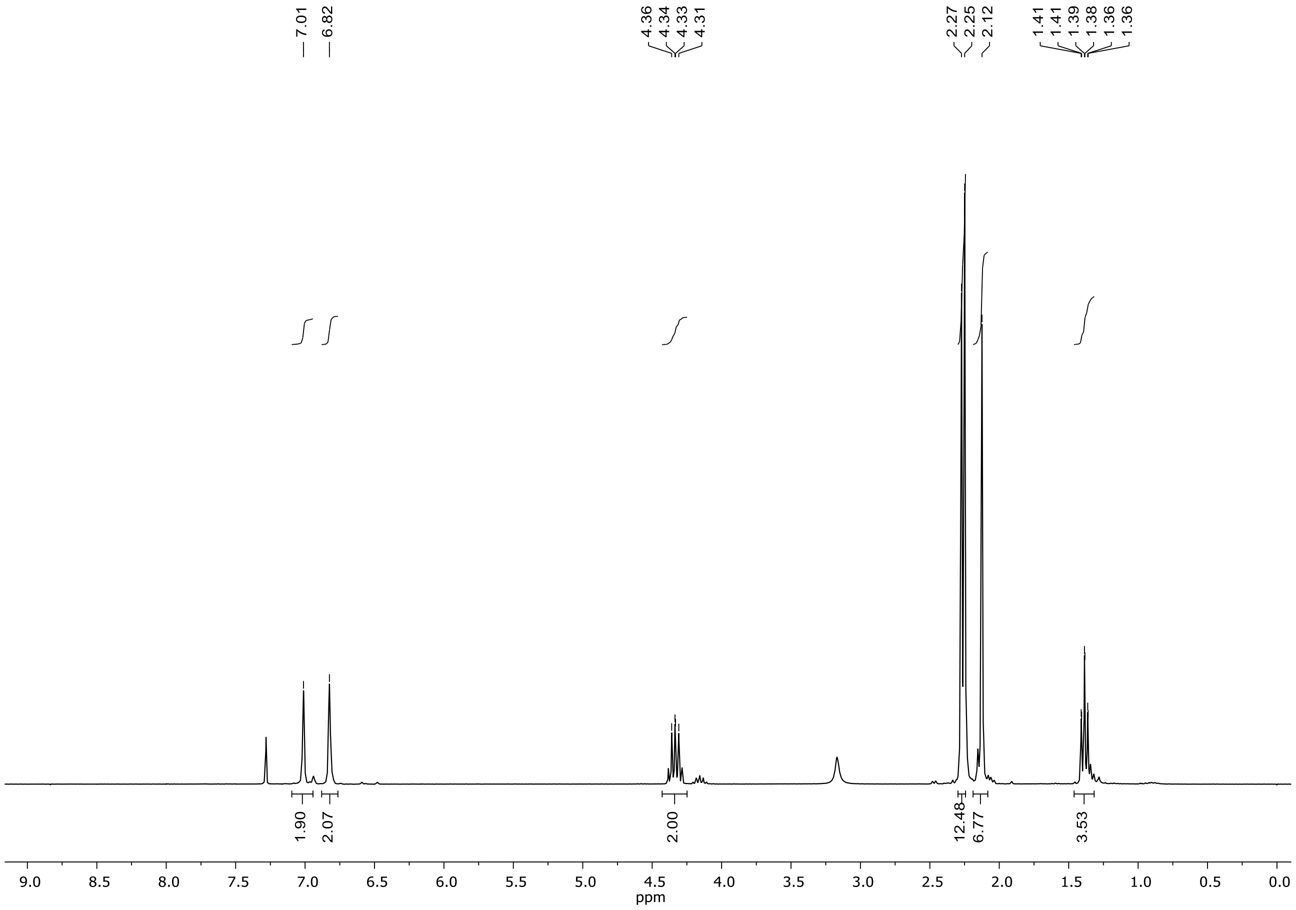

Ethyl bis-(2,4,6-trimethylphenyl) phosphate (**3e,** ^{13}C NMR, 75 MHz, $CDCl_3$).

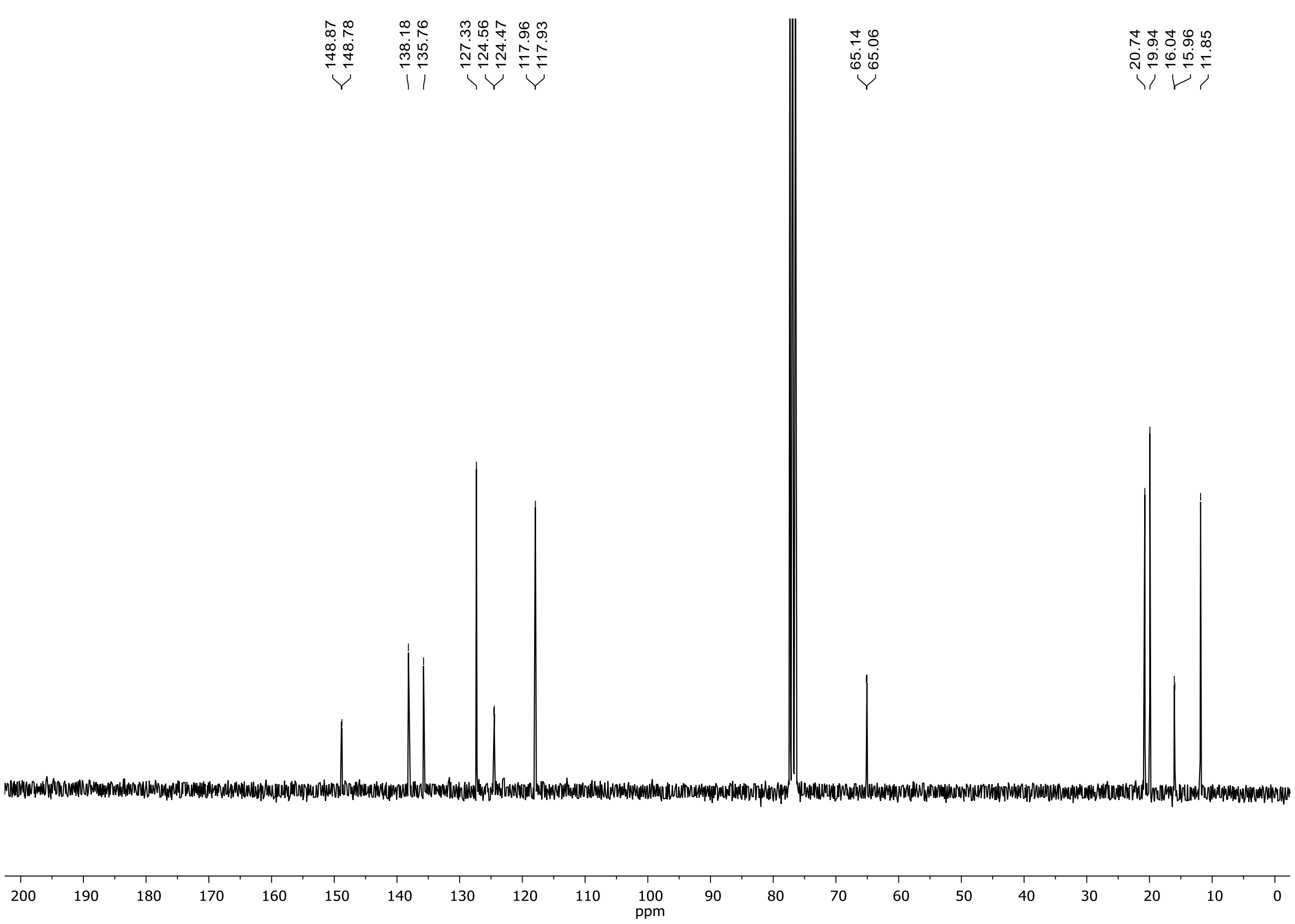

4,4’-Diphenoxybiphenyl (**4b,** ^{1}H NMR, 300 MHz, $CDCl_3$).

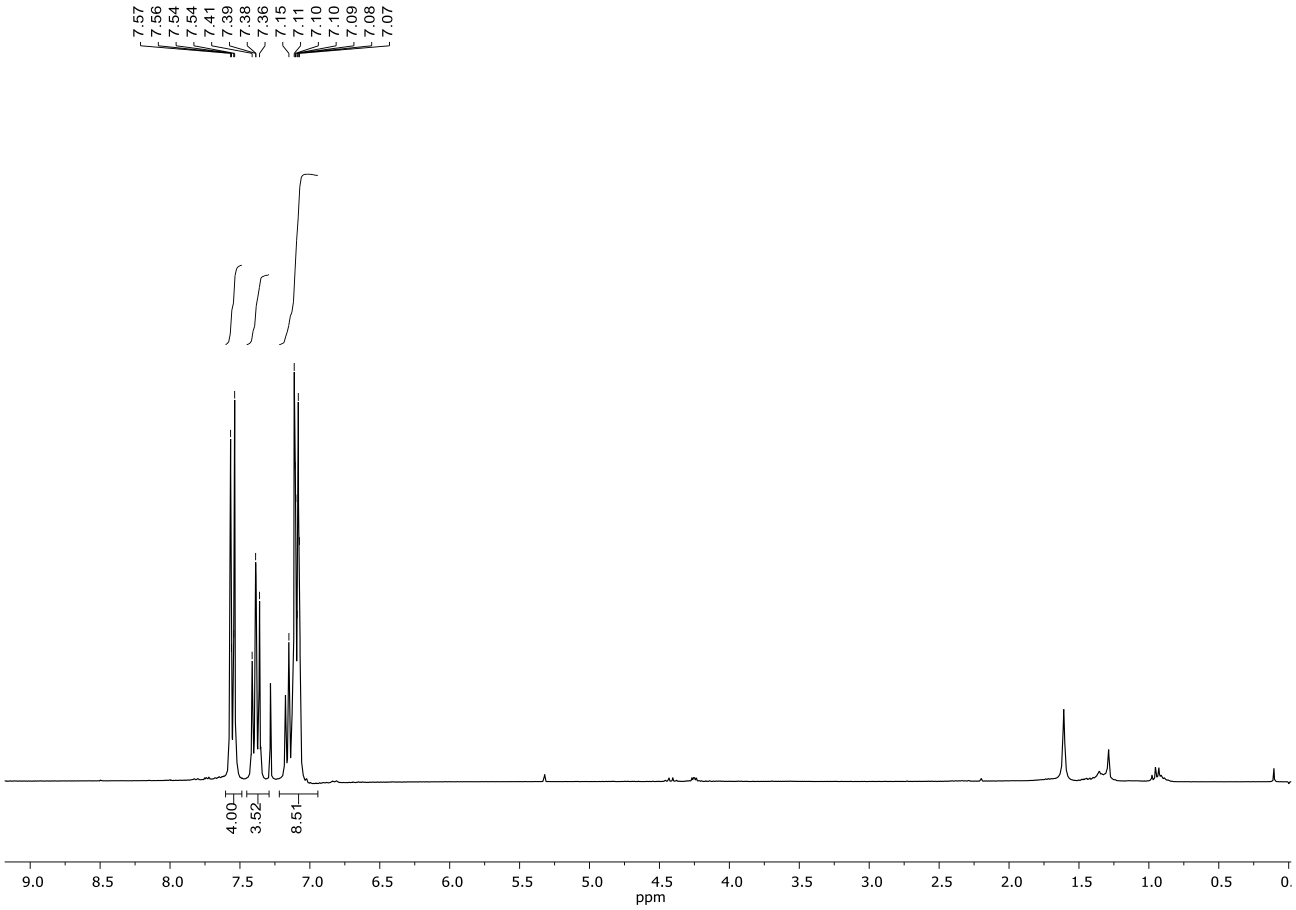

4,4’-Diphenoxybiphenyl (**4b,** ^{13}C NMR, 75 MHz, $CDCl_3$).

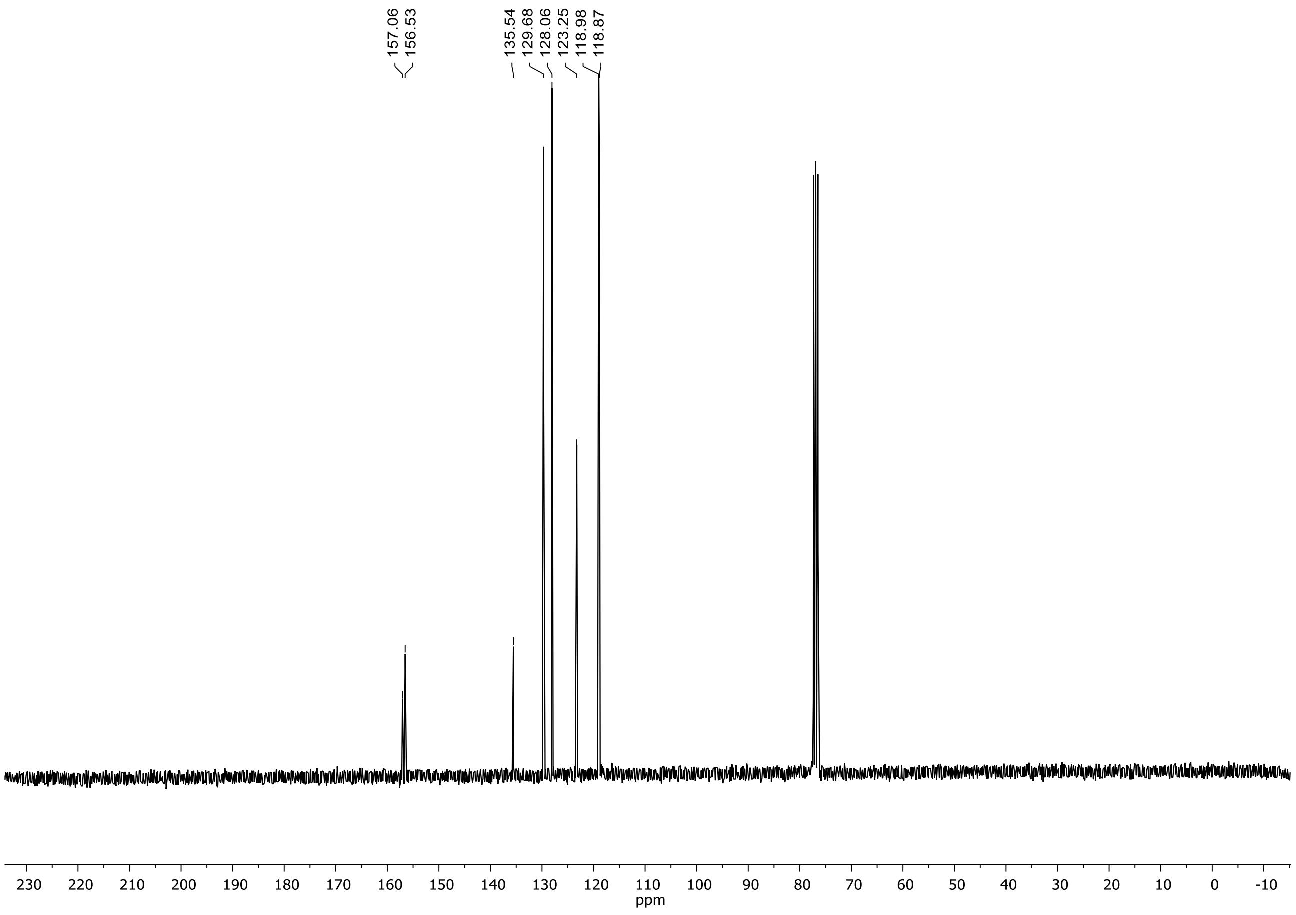

4,4’-Di-*tert*-butylbiphenyl (**4c,** ^{1}H NMR, 300 MHz, $CDCl_3$).

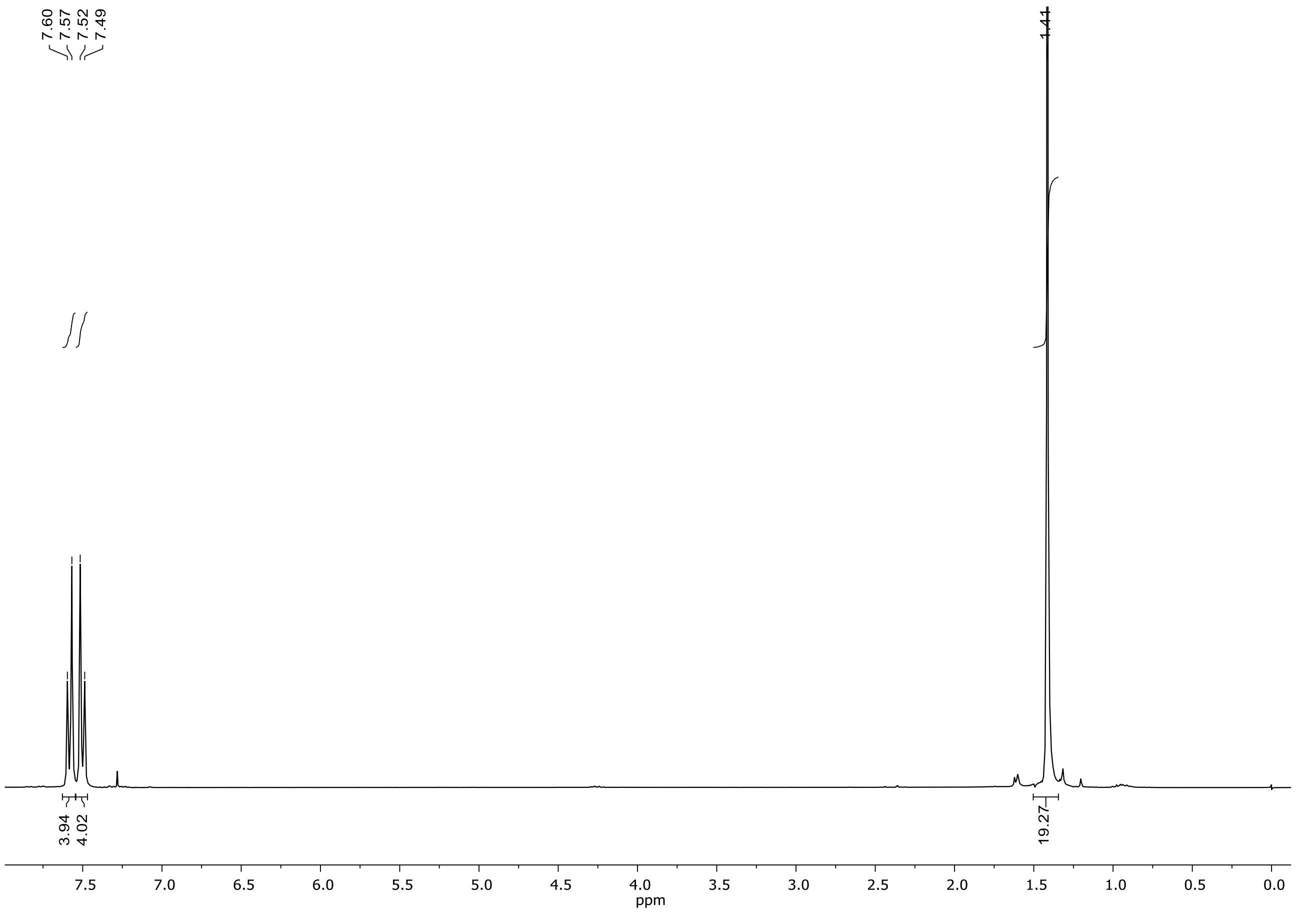

4,4'-Di-*tert*-butylbiphenyl (**4c,** ^{13}C NMR, 75 MHz, $CDCl_3$).

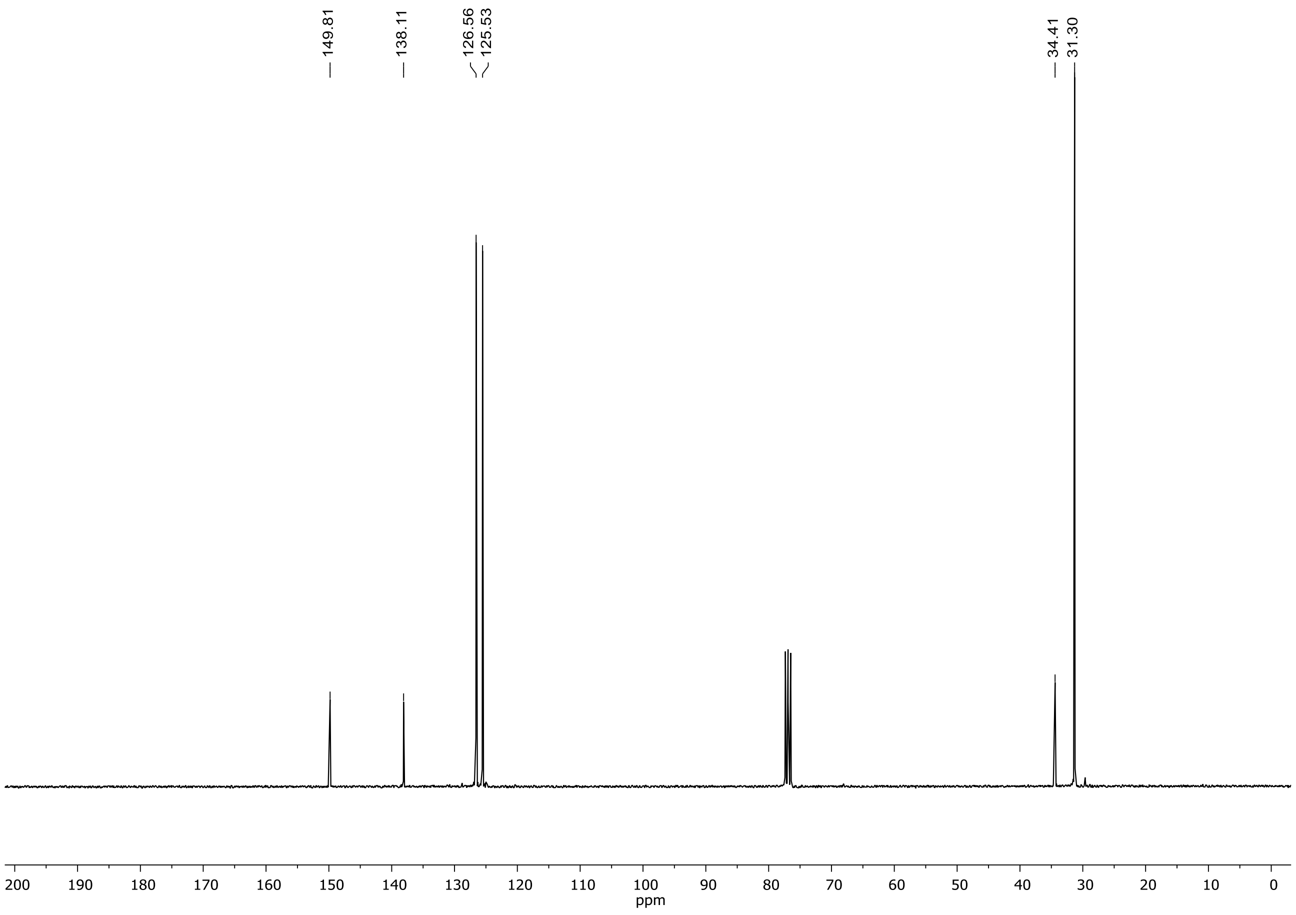

3,3’-Dimethoxybiphenyl (**4d,** ^{1}H NMR, 300 MHz, $CDCl_3$).

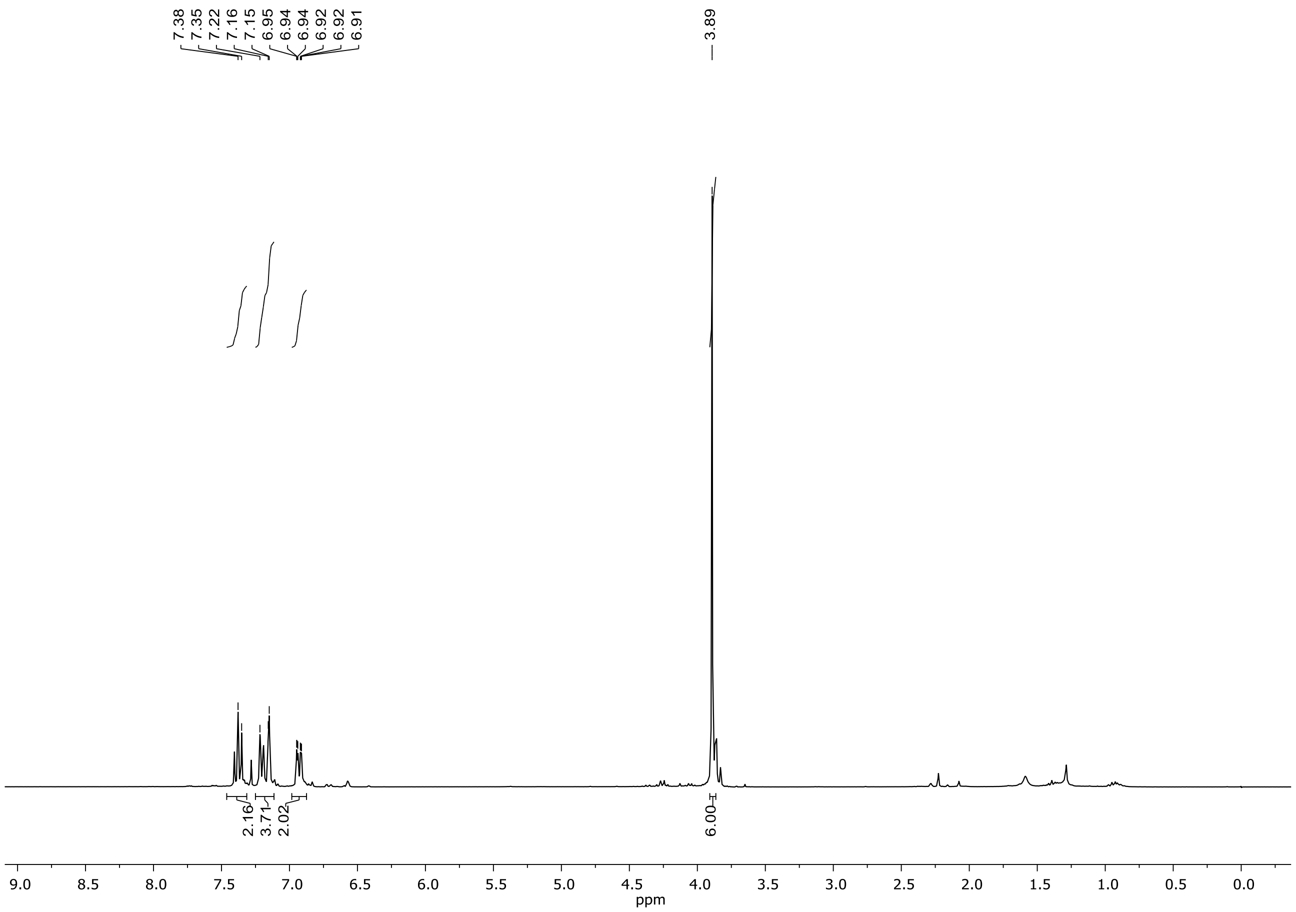

3,3'-Dimethoxybiphenyl (**4d,** ^{13}C NMR, 75 MHz, CDCl$_3$).

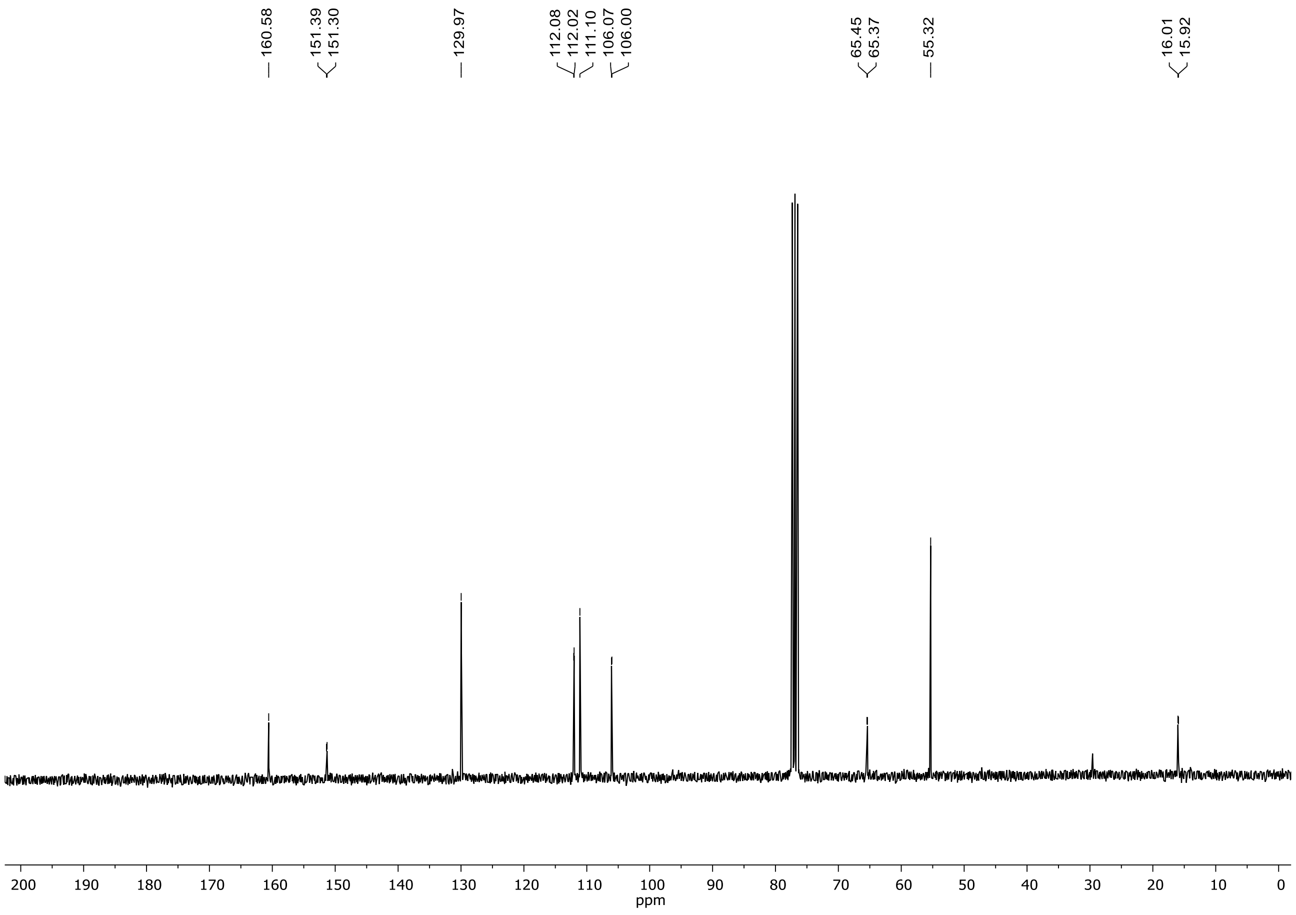

Supplement: File 1 — Experimental section, fluorescence, and 1H and 13C NMR spectra. [file Beilstein_J_Org_Chem-16-3008-s001.pdf]
